# Supplementary material for: Dexfenfluramine and Pergolide Cause Heart Valve Disease via Valve Metabolic Reprogramming and Ongoing Matrix Remodeling
Source: Int J Mol Sci. 2020 Jun 3;21(11):4003. doi: 10.3390/ijms21114003 (PMC7312197; doi:10.3390/ijms21114003)
Supplement: Supplementary file 1 [file ijms-21-04003-s001.pdf]

SUPPLEMENTARY MATERIAL

Dexfenfluramine and Pergolide Cause Heart Valve Disease via Valve Metabolic Reprogramming and Ongoing Matrix Remodeling

Cécile Oury [1*], Patrick Maréchal [1], Nathalie Donis [1], Alexia Hulin [1], Julien Tridetti [1], Mai-Linh Nguyen [1], Raluca Dulgheru [1], Marianne Fillet [2], Alain Nchimi [1] and Patrizio Lancellotti [1,3*]

Content:

Figure S1
Table S1
Table S2

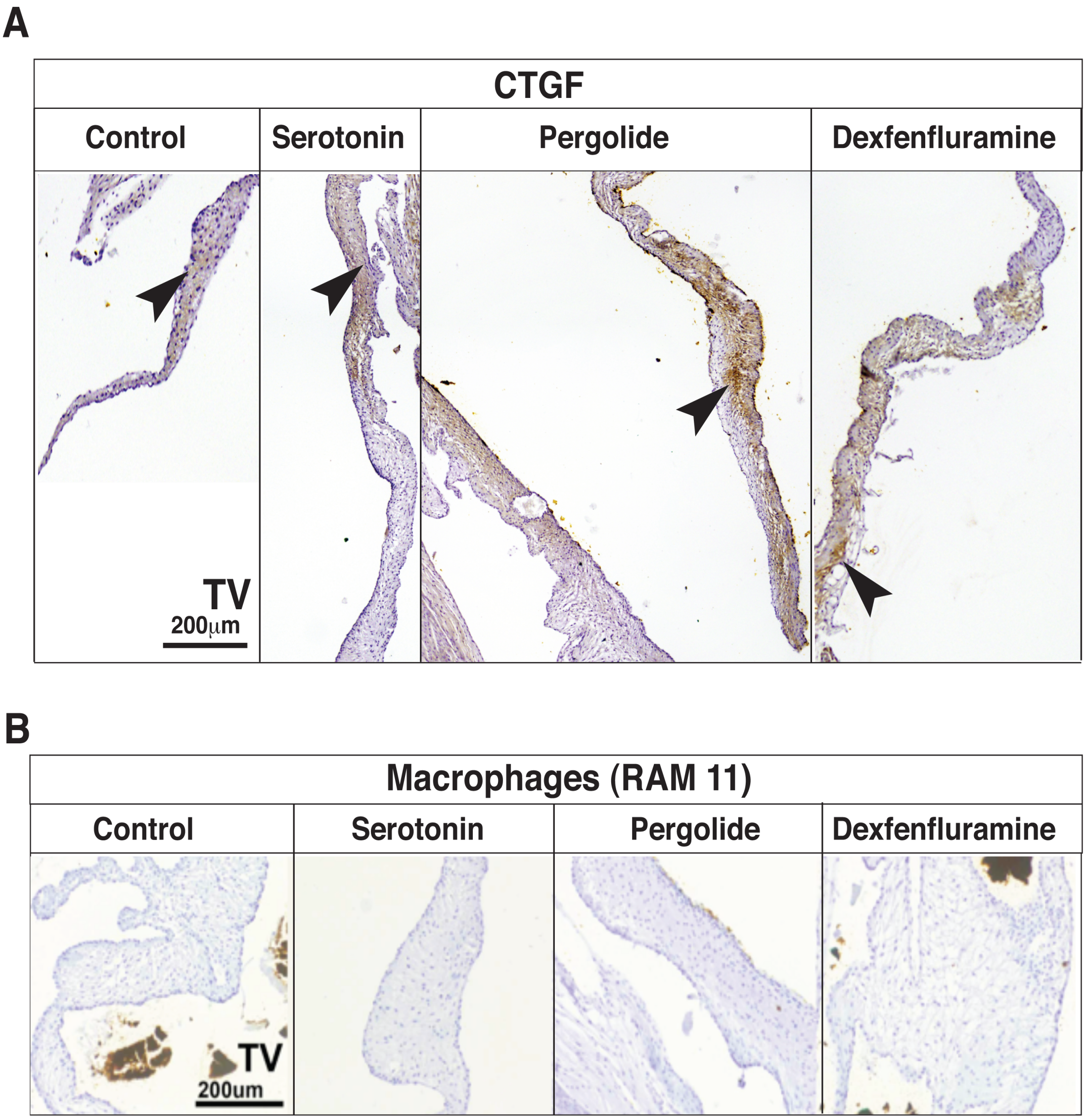

Figure S1. A. CTGF-rich areas in tricuspid valve leaflets of drug-treated rabbits. Representative anti-CTGF staining performed on sections of tricuspid valve (TV) leaflet from control, serotonin, pergolide and dexfenfluramine-treated rabbits, as indicated. Scale bar = 200◎m. **B. Drug treatments do not promote macrophage infiltration in TV leaflet.** Representative macrophage (RAM11) staining performed on sections of tricuspid valve (TV) leaflet from control, serotonin, pergolide and dexfenfluramine-treated rabbits, as indicated. Scale bar = 200◎m.

Table S1. Full list of DEGs common and specific to the different treatment groups as compared to untreated controls

**COMMON DEGs pergolide, dexfenfluramine, serotonin**		**Log FC**		
ID	GENE ID	SEROTONIN	PERGOLIDE	DEXFENFLURAMINE
ENSOCUG00000009905	RPL37A	1,575	1,23	1,12
ENSOCUG00000000658	BMP3	0,968	0,85	0,97
ENSOCUG00000009329	NR1D2	0,905	1,09	0,94
ENSOCUG00000002898	FBLN7	0,905	1,35	0,68
ENSOCUG00000016945	ITIH5	0,858	0,92	0,52
ENSOCUG00000016964	MGP	0,814	0,77	0,68
ENSOCUG00000013396	EDN1	0,813	0,59	0,60
ENSOCUG00000012043	CAMKK1	0,811	0,96	0,98
ENSOCUG00000017302	SLIT3	0,802	0,70	0,56
ENSOCUG00000015234	COL14A1	0,738	0,78	0,60
ENSOCUG00000024041	LXN	0,663	0,70	0,59
ENSOCUG00000003155	PLAU	0,661	0,82	0,82
ENSOCUG00000026419	C1QTNF3	0,643	0,87	0,53
ENSOCUG00000016227	SVEP1	0,630	0,78	0,55
ENSOCUG00000005778	CPED1	0,629	0,83	0,57
ENSOCUG00000010718	TIMP1	0,613	0,50	0,64
ENSOCUG00000006145	CHRDL1	0,605	0,79	0,63
ENSOCUG00000008037	FAM13A	0,590	0,64	0,34
ENSOCUG00000016659	EMB	0,577	0,45	0,47
ENSOCUG00000014803	RPF1	0,555	0,58	0,47
ENSOCUG00000000338	ATP1B3	0,545	1,03	1,15
ENSOCUG00000025358	PROS1	0,521	0,44	0,78
ENSOCUG00000000713	TMEM135	0,518	0,84	0,65
ENSOCUG00000005115	FAP	0,483	0,42	0,40
ENSOCUG00000010772	DCHS2	0,481	0,96	0,38
ENSOCUG00000004137	PRSS23	0,479	0,51	0,48
ENSOCUG00000029492	SF3B1	0,475	0,298	0,41
ENSOCUG00000024081	CD63	0,464	0,355	0,28
ENSOCUG00000011150	TNFRSF11B	0,449	0,67	0,52
ENSOCUG00000012264	COL1A2	0,439	0,91	0,95
ENSOCUG00000003706	RPL8	0,437	0,38	0,47
ENSOCUG00000011090	CTSK	0,418	0,54	0,59
ENSOCUG00000029387	ADD3	0,418	0,32	0,22
ENSOCUG00000015749	RPS4X	0,416	1,25	1,33
ENSOCUG00000029144	ZNF260	0,413	0,46	0,55
ENSOCUG00000002392	RPL31	0,406	1,60	1,66
ENSOCUG00000014975	OLFML1	0,405	0,53	0,31

ENSOCUG00000013509	SKIL	0,392	0,41	0,49
ENSOCUG00000013281	SFMBT2	0,391	0,45	0,61
ENSOCUG00000016361	CD302	0,382	0,45	0,38
ENSOCUG00000014455	ENPP1	0,364	0,38	0,40
ENSOCUG00000017949	HAO2	0,350	0,98	1,05
ENSOCUG00000026336	RPS19	0,343	0,62	0,62
ENSOCUG00000013687	IL13RA1	0,338	0,62	0,49
ENSOCUG00000024991	ALPK1	0,330	0,47	0,55
ENSOCUG00000025870	C5orf42	0,315	0,48	0,51
ENSOCUG00000017627	UGGT2	0,297	0,59	0,44
ENSOCUG00000008254	SEC24A	0,284	0,22	0,26
ENSOCUG00000029127	CD164	0,281	0,53	0,46
ENSOCUG00000006321	CNN3	0,271	0,40	0,46
ENSOCUG00000012361	ATF7IP	0,199	0,19	0,22
ENSOCUG00000006646	UBR3	-0,187	-0,24	-0,39
ENSOCUG00000012842	HSP90AB1	-0,204	-0,45	-0,57
ENSOCUG00000004753	KANK1	-0,243	-0,53	-0,35
ENSOCUG00000021353	HECA	-0,247	-0,33	-0,36
ENSOCUG00000029143	DSC3	-0,263	-0,28	-0,30
ENSOCUG00000017057	SRPRA	-0,267	-0,22	-0,22
ENSOCUG00000029112	ND5	-0,267	-0,97	-1,63
ENSOCUG00000000156	TRAK2	-0,269	-0,49	-0,76
ENSOCUG00000007809	ATP5F1	-0,274	-0,35	-0,56
ENSOCUG00000001430	AHSA1	-0,288	-0,54	-0,59
ENSOCUG00000000706	FHOD3	-0,303	-0,47	-0,75
ENSOCUG00000029103	COX3	-0,306	-0,65	-1,15
ENSOCUG00000005218	MTUS1	-0,306	-0,44	-0,67
ENSOCUG00000013064	TLN2	-0,312	-0,22	-0,31
ENSOCUG00000015705	MAST4	-0,315	-0,30	-0,37
ENSOCUG00000001629	CWC15	-0,316	-0,28	-0,42
ENSOCUG00000003452	PPM1K	-0,327	-0,73	-0,94
ENSOCUG00000010178	IMMT	-0,327	-0,50	-0,77
ENSOCUG00000007441	USP28	-0,328	-0,38	-0,63
ENSOCUG00000005926	USP4	-0,328	-0,43	-0,47
ENSOCUG00000020976	DRAP1	-0,334	-1,56	-1,57
ENSOCUG00000015632	PDHB	-0,336	-0,25	-0,72
ENSOCUG00000004329	PSMC5	-0,344	-0,57	-0,63
ENSOCUG00000009660	OSBPL11	-0,348	-0,73	-0,61
ENSOCUG00000025649	TPI1	-0,350	-0,24	-0,70
ENSOCUG00000029115	CYTB	-0,357	-1,13	-1,72
ENSOCUG00000022350	CLOCK	-0,358	-0,31	-0,33
ENSOCUG00000014344	MPP7	-0,362	-0,50	-0,82

ENSOCUG00000029101	ATP8	-0,365	-0,98	-1,41
ENSOCUG00000015845	RHOBTB3	-0,367	-0,28	-0,32
ENSOCUG00000009465	AGL	-0,383	-0,28	-0,58
ENSOCUG00000016510	RUVBL1	-0,385	-0,39	-0,72
ENSOCUG00000014610	ABCC4	-0,387	-0,25	-0,47
ENSOCUG00000029113	ND6	-0,397	-1,96	-2,32
ENSOCUG00000003263	SLCO5A1	-0,405	-0,31	-0,77
ENSOCUG00000013647	SORBS1	-0,408	-0,39	-0,52
ENSOCUG00000015590	DLAT	-0,411	-0,37	-0,70
ENSOCUG00000001847	TMBIM6	-0,414	-0,92	-0,96
ENSOCUG00000010895	SVIL	-0,418	-0,61	-0,70
ENSOCUG00000006697	HK1	-0,423	-0,62	-0,73
ENSOCUG00000013750	WWTR1	-0,427	-0,64	-0,25
ENSOCUG00000012426	PRKAR2A	-0,430	-0,25	-0,52
ENSOCUG00000015606	DLST	-0,433	-0,33	-0,67
ENSOCUG00000014603	FKBP4	-0,439	-0,69	-0,77
ENSOCUG00000006793	LMO7	-0,444	-0,50	-0,80
ENSOCUG00000008971	CPEB4	-0,448	-0,39	-0,41
ENSOCUG00000013561	GFM1	-0,449	-0,51	-0,71
ENSOCUG00000003773	DTNA	-0,454	-0,44	-0,79
ENSOCUG00000000245	ABCF2	-0,454	-0,47	-0,61
ENSOCUG00000014175	RAF1	-0,460	-0,35	-0,35
ENSOCUG00000000525	TIMM23B	-0,461	-0,92	-1,08
ENSOCUG00000029096	COX1	-0,466	-0,75	-1,25
ENSOCUG00000001697	PTPRK	-0,466	-0,45	-0,58
ENSOCUG00000003236	PPP1R12B	-0,477	-0,47	-0,68
ENSOCUG00000007880	NIPSNAP3A	-0,479	-0,63	-0,82
ENSOCUG00000003521	ANK3	-0,485	-0,57	-1,05
ENSOCUG00000016777	TTN	-0,489	-0,47	-1,05
ENSOCUG00000000928	MFN1	-0,489	-0,26	-0,34
ENSOCUG00000005112	FBXO32	-0,490	-0,39	-0,46
ENSOCUG00000009939	MYPN	-0,493	-0,55	-1,24
ENSOCUG00000011228	PDE4DIP	-0,500	-0,41	-0,89
ENSOCUG00000011508	PDZD2	-0,509	-0,36	-0,44
ENSOCUG00000013549	MLF1	-0,510	-1,05	-1,64
ENSOCUG00000016916	CEP85	-0,522	-0,68	-0,78
ENSOCUG00000014032	CHCHD3	-0,522	-0,71	-1,10
ENSOCUG00000000324	PSTPIP2	-0,540	-0,40	-0,51
ENSOCUG00000014668	PPARGC1A	-0,545	-0,77	-1,21
ENSOCUG00000016429	ABT1	-0,549	-0,73	-0,52
ENSOCUG00000000477	RALY	-0,552	-0,43	-0,45
ENSOCUG00000010297	SIPA1L2	-0,553	-0,49	-0,83

ENSOCUG00000015112	CLUH	-0,557	-0,53	-0,62
ENSOCUG00000006966	ACADVL	-0,573	-0,66	-0,72
ENSOCUG00000016158	PMVK	-0,577	-0,49	-0,54
ENSOCUG00000025023	GAPDH	-0,585	-1,03	-1,44
ENSOCUG00000022357	PDK2	-0,596	-0,87	-0,86
ENSOCUG00000013564	TRIM63	-0,599	-0,97	-1,32
ENSOCUG00000009096	LIAS	-0,602	-0,41	-1,07
ENSOCUG00000016643	ITGB1BP2	-0,605	-0,40	-1,04
ENSOCUG00000010062	DYSF	-0,616	-0,34	-0,80
ENSOCUG00000012333	GOT1	-0,622	-0,56	-1,07
ENSOCUG00000002041	UQCRC1	-0,627	-0,44	-0,67
ENSOCUG00000008004	PDHX	-0,657	-0,47	-0,64
ENSOCUG00000011599	CACNA1C	-0,664	-0,35	-0,68
ENSOCUG00000014122	TPM1	-0,665	-0,66	-1,21
ENSOCUG00000011935	PPTC7	-0,672	-0,73	-0,82
ENSOCUG00000009225	MYOCD	-0,677	-0,90	-1,09
ENSOCUG00000011610	VEGFA	-0,679	-0,81	-0,74
ENSOCUG00000014974	COQ9	-0,695	-0,45	-0,67
ENSOCUG00000002272	MYH7B	-0,701	-1,17	-1,29
ENSOCUG00000010393	FXR2	-0,734	-0,52	-0,55
ENSOCUG00000006728	MYOM3	-0,751	-0,81	-1,24
ENSOCUG00000012191	KIAA1191	-0,751	-1,01	-1,10
ENSOCUG00000011879	KLHL31	-0,759	-0,57	-1,13
ENSOCUG00000026781	F5	-0,764	-0,85	-1,02
ENSOCUG00000014136	ATP6V1F	-0,767	-0,69	-1,03
ENSOCUG00000022015	LDHB	-0,774	-0,64	-1,14
ENSOCUG00000028008	APOBEC2	-0,804	-0,61	-1,03
ENSOCUG00000002852	EMC9	-0,813	-0,72	-1,10
ENSOCUG00000016944	BVES	-0,817	-0,58	-0,85
ENSOCUG00000012286	ASB14	-0,837	-1,53	-2,04
ENSOCUG00000014608	FABP3	-0,852	-0,96	-1,14
ENSOCUG00000003829	EZR	-0,879	-0,90	-0,63
ENSOCUG00000022337	SOD2	-0,898	-1,03	-1,39
ENSOCUG00000010416	KCNJ2	-0,929	-0,81	-1,12
ENSOCUG00000007837	LRRC39	-0,934	-0,84	-1,33
ENSOCUG00000007967	FHL2	-0,936	-0,87	-1,29
ENSOCUG00000011978	FAM124B	-1,004	-0,71	-0,92
ENSOCUG00000026994	TUBA4A	-1,042	-1,28	-1,74
ENSOCUG00000008337	PCYOX1L	-1,059	-0,67	-0,86
ENSOCUG00000021380	SMCO1	-1,146	-1,27	-1,17
ENSOCUG00000007214	TMEM116	-1,641	-1,76	-2,05
ENSOCUG00000000145	NFIL3	-1,889	-1,45	-0,75

ENSOCUG00000016839	CSRNP3	-1,970	-3,92	-2,26
ENSOCUG00000005940	ARNTL	-2,482	-2,54	-1,35

**DEGs pergolide and dexfenfluramine**		**LogFC**	
**ID**	**GENE ID**	**PERGOLIDE**	**DEXFENFLURAMINE**
ENSOCUG00000007765	PSAT1	3,020	2,481
ENSOCUG00000024190	YWHAZ	2,772	2,671
ENSOCUG00000000725	MC5R	2,474	2,178
ENSOCUG00000018543	SNORA73B	2,185	2,052
ENSOCUG00000009058	HP	1,924	1,488
ENSOCUG00000022125	IL34	1,882	1,553
ENSOCUG00000024096	S100A6	1,881	1,827
ENSOCUG00000023610	CPNE5	1,866	1,939
ENSOCUG00000016072	RPS7	1,824	1,871
ENSOCUG00000015394	LRRC32	1,748	1,722
ENSOCUG00000013134	ST8SIA1	1,707	1,485
ENSOCUG00000005145	EEPD1	1,688	1,009
ENSOCUG00000025329	MARVELD1	1,658	1,880
ENSOCUG00000006135	FAU	1,647	1,591
ENSOCUG00000024506	PHKB	1,632	1,524
ENSOCUG00000027232	ATP8A2	1,600	1,333
ENSOCUG00000027115	TPRA1	1,572	1,567
ENSOCUG00000020087	SNORA76	1,515	1,403
ENSOCUG00000027459	RPS26	1,511	1,470
ENSOCUG00000010496	OSGIN1	1,474	1,720
ENSOCUG00000006890	TMEM64	1,472	1,714
ENSOCUG00000025006	RPL9	1,436	1,467
ENSOCUG00000005175	QSOX1	1,413	1,747
ENSOCUG00000018956	mir-29C	1,373	1,638
ENSOCUG00000000983	PLSCR3	1,365	1,656
ENSOCUG00000029359	UBE2E3	1,364	1,486
ENSOCUG00000029169	ZFP36L2	1,359	1,500
ENSOCUG00000027353	SONRA62	1,357	1,559
ENSOCUG00000004372	Dgcr6	1,342	1,440
ENSOCUG00000014962	RPL22L1	1,316	1,202
ENSOCUG00000012615	BMPER	1,315	1,402
ENSOCUG00000008644	IRS3	1,310	1,278
ENSOCUG00000019496	SNORA29	1,299	1,97
ENSOCUG00000023567	TOR1AIP2	1,276	0,956
ENSOCUG00000004979	KDELR3	1,273	1,332
ENSOCUG00000002836	PCK2	1,246	0,884
ENSOCUG00000012631	PPIA	1,235	1,128
ENSOCUG00000014661	NAAA	1,234	1,465
ENSOCUG00000013567	GRN	1,234	1,209
ENSOCUG00000004795	UFM1	1,228	1,103

ENSOCUG00000022712	PIK3R5	1,228	1,550
ENSOCUG00000026029	SERPINH1	1,226	1,220
ENSOCUG00000006229	TYRO3	1,224	1,279
ENSOCUG00000014420	CD53	1,221	1,207
ENSOCUG00000006801	MCM10	1,219	1,090
ENSOCUG00000004138	SERINC5	1,217	1,104
ENSOCUG00000010950	TMED7	1,215	1,112
ENSOCUG00000002902	DUSP6	1,214	0,630
ENSOCUG00000014907	WIF1	1,211	1,131
ENSOCUG00000007508	NID1	1,206	0,824
ENSOCUG00000024510	ARL5A	1,194	0,899
ENSOCUG00000006887	CYP8B1	1,186	1,074
ENSOCUG00000010549	MMD	1,174	0,796
ENSOCUG00000003250	NDN	1,155	1,423
ENSOCUG00000004167	UROD	1,152	0,924
ENSOCUG00000015952	TIMP4	1,151	0,906
ENSOCUG00000007606	MAP2K6	1,142	0,973
ENSOCUG00000012562	WDCP	1,135	1,242
ENSOCUG00000005946	SLC44A1	1,117	1,012
ENSOCUG00000000427	PM20D1	1,114	1,005
ENSOCUG00000014225	SLCO2B1	1,113	1,440
ENSOCUG00000009424	LAMA1	1,102	0,724
ENSOCUG00000008234	ZADH2	1,100	0,774
ENSOCUG00000017509	SERPINI1	1,099	1,215
ENSOCUG00000015433	ZKSCAN1	1,091	1,430
ENSOCUG00000004954	AMACR	1,087	0,695
ENSOCUG00000014239	TMEM56-RWDD3	1,085	1,184
ENSOCUG00000014730	FZD2	1,079	1,280
ENSOCUG00000022366	snoU85	1,078	1,283
ENSOCUG00000027359	TSPAN11	1,076	0,945
ENSOCUG00000009326	TMEM167A	1,075	1,205
ENSOCUG00000029357	RPL34	1,068	0,991
ENSOCUG00000013610	ASB3	1,055	0,964
ENSOCUG00000005900	FBLN2	1,054	0,805
ENSOCUG00000013017	ELOVL2	1,050	1,119
ENSOCUG00000010292	EFHC2	1,049	1,064
ENSOCUG00000012881	COL1A1	1,046	1,154
ENSOCUG00000025474	RPLP0	1,041	1,065
ENSOCUG00000006055	RNF13	1,040	0,824
ENSOCUG00000001726	WNT11	1,029	0,976
ENSOCUG00000013931	LEPROTL1	1,027	1,352
ENSOCUG00000011735	C1QTNF2	1,022	1,008
ENSOCUG00000018960	SNORA3A	1,014	0,938
ENSOCUG00000014988	COL3A1	1,013	0,951

ENSOCUG00000013992	TSPAN15	1,009	1,007
ENSOCUG00000023265	SNORD2	1,003	0,872
ENSOCUG00000015863	C5orf63	1,000	1,080
ENSOCUG00000027003	Hist1h2bo	0,994	1,047
ENSOCUG00000014194	NDUFA4	0,990	0,700
ENSOCUG00000024792	HDAC7	0,987	0,933
ENSOCUG00000024365	ARF5	0,986	1,162
ENSOCUG00000023327	RPL39	0,984	1,046
ENSOCUG00000003059	CTBS	0,977	0,692
ENSOCUG00000012637	EPHB3	0,977	1,017
ENSOCUG00000005504	POLR1C	0,974	0,984
ENSOCUG00000016685	NOX4	0,973	0,742
ENSOCUG00000001802	COPZ2	0,973	0,637
ENSOCUG00000014315	MCTS1	0,964	0,747
ENSOCUG00000007808	CAVIN3	0,962	0,987
ENSOCUG00000024110	PALD1	0,953	0,699
ENSOCUG00000028053	ASPN	0,952	0,702
ENSOCUG00000007418	PDK3	0,952	0,920
ENSOCUG00000025470	XBP1	0,947	1,028
ENSOCUG00000024298	NINJ1	0,947	1,298
ENSOCUG00000026159	KIAA1755	0,943	1,124
ENSOCUG00000008227	MOB1A	0,942	0,708
ENSOCUG00000024954	LAMP2	0,942	0,936
ENSOCUG00000007039	C4A	0,942	1,232
ENSOCUG00000023076	SFT2D2	0,938	0,958
ENSOCUG00000000754	CYP39A1	0,937	1,048
ENSOCUG00000029701	TMEM120B	0,936	1,162
ENSOCUG00000011161	LRRC17	0,931	0,981
ENSOCUG00000010588	EIF6	0,928	0,976
ENSOCUG00000008914	AHDC1	0,924	0,879
ENSOCUG00000004456	MAGED2	0,922	0,829
ENSOCUG00000005086	PSMG2	0,922	0,723
ENSOCUG00000016704	GXYLT2	0,922	1,087
ENSOCUG00000021605	ARAP1	0,914	0,935
ENSOCUG00000009771	C1D	0,914	0,665
ENSOCUG00000005147	TMEM161B	0,914	0,876
ENSOCUG00000007824	G2E3	0,910	0,753
ENSOCUG00000006315	RFFL	0,906	0,891
ENSOCUG00000000092	SERPINF1	0,905	0,601
ENSOCUG00000014407	NUP35	0,903	0,659
ENSOCUG00000002919	TINF2	0,903	0,876
ENSOCUG00000002375	VAMP8	0,902	0,959
ENSOCUG00000025005	ARSI	0,900	0,741
ENSOCUG00000000440	CYP7B1	0,899	0,828
ENSOCUG00000002874	PTK7	0,899	1,187

ENSOCUG00000010233	HNRNPLL	0,890	0,791
ENSOCUG00000012681	TP53I13	0,890	0,915
ENSOCUG00000003680	GSTA4	0,885	1,027
ENSOCUG00000026403	RAB32	0,884	0,729
ENSOCUG00000024314	CDC42SE1	0,884	0,662
ENSOCUG00000010690	FAM126B	0,880	0,814
ENSOCUG00000014740	SLC37A2	0,880	0,931
ENSOCUG00000001888	H2AFZ	0,878	0,753
ENSOCUG00000005740	ARL6IP1	0,870	0,934
ENSOCUG00000017494	MRC2	0,869	1,132
ENSOCUG00000004359	SERPINE2	0,863	0,933
ENSOCUG00000004889	VPS18	0,858	0,894
ENSOCUG00000029427	MANEA	0,858	0,639
ENSOCUG00000010517	SLC30A7	0,857	0,873
ENSOCUG00000009050	RELN	0,857	0,727
ENSOCUG00000021304	MYL12B	0,855	0,518
ENSOCUG00000002466	STAT4	0,854	0,865
ENSOCUG00000012561	SLC43A3	0,853	0,891
ENSOCUG00000012397	TSC22D3	0,847	0,964
ENSOCUG00000001317	DPY19L4	0,847	0,920
ENSOCUG00000014385	CPQ	0,843	0,821
ENSOCUG00000012985	TMEM181	0,842	1,211
ENSOCUG00000011774	FBXL5	0,841	0,786
ENSOCUG00000007773	IFT20	0,839	0,616
ENSOCUG00000008584	ERI3	0,838	0,821
ENSOCUG00000011908	ARPC3	0,838	0,656
ENSOCUG00000017711	LANCL1	0,838	0,663
ENSOCUG00000003358	ACTR10	0,833	0,781
ENSOCUG00000016243	MT3	0,832	0,808
ENSOCUG00000005689	MAP3K12	0,828	0,892
ENSOCUG00000000581	NAGLU	0,827	1,150
ENSOCUG00000011860	APAF1	0,826	0,820
ENSOCUG00000003152	SCAP	0,824	1,062
ENSOCUG00000026081	NIT2	0,824	0,640
ENSOCUG00000027290	PTCD3	0,824	0,44
ENSOCUG00000005019	CAND1	0,824	0,806
ENSOCUG00000011284	SLC7A2	0,819	0,674
ENSOCUG00000000721	PAMR1	0,817	0,802
ENSOCUG00000004060	AP2B1	0,815	0,746
ENSOCUG00000013715	C5orf34	0,815	0,768
ENSOCUG00000019650	SNORA42	0,809	2,44
ENSOCUG00000027106	HAS2	0,808	0,777
ENSOCUG00000008470	DCTN5	0,807	0,847
ENSOCUG00000011789	ARMC2	0,807	0,930
ENSOCUG00000017029	BET1	0,802	0,889

ENSOCUG00000004831	DIP2A	0,799	0,786
ENSOCUG00000008044	DCAF12	0,798	0,705
ENSOCUG00000006353	KBTBD4	0,797	0,756
ENSOCUG00000007801	WDR77	0,797	0,730
ENSOCUG00000005571	CA5B	0,797	0,727
ENSOCUG00000016783	DTWD1	0,797	0,613
ENSOCUG00000006445	FZD4	0,796	0,522
ENSOCUG00000017049	FAM118B	0,794	0,817
ENSOCUG00000010547	HLF	0,794	0,826
ENSOCUG00000005647	RP2	0,789	0,904
ENSOCUG00000009190	HEXA	0,787	1,008
ENSOCUG00000027478	SGIP1	0,787	0,738
ENSOCUG00000007892	FBXL4	0,785	0,857
ENSOCUG00000014366	UGCG	0,785	0,674
ENSOCUG00000009140	RPS2	0,785	0,711
ENSOCUG00000004396	NQO1	0,783	0,505
ENSOCUG00000026678	SP3	0,783	0,675
ENSOCUG00000013648	SLC16A9	0,781	0,961
ENSOCUG00000012539	TMEM19	0,780	0,770
ENSOCUG00000004336	FAR1	0,778	0,640
ENSOCUG00000004786	PRDX4	0,777	0,932
ENSOCUG00000001072	BCO1	0,777	0,743
ENSOCUG00000006473	ATP6V1A	0,776	0,484
ENSOCUG00000007898	SSR3	0,776	0,811
ENSOCUG00000005945	AKR1A1	0,771	0,785
ENSOCUG00000023883	VAMP3	0,770	0,726
ENSOCUG00000002368	GGCX	0,770	0,887
ENSOCUG00000014665	REPS2	0,770	0,764
ENSOCUG00000013866	SELT	0,769	0,895
ENSOCUG00000006005	PIGC	0,764	0,707
ENSOCUG00000009242	C5orf51	0,762	0,800
ENSOCUG00000024967	ID1	0,761	0,704
ENSOCUG00000000532	WLS	0,758	0,951
ENSOCUG00000006953	SLC29A3	0,757	0,687
ENSOCUG00000026130	GSTZ1	0,755	0,591
ENSOCUG00000006994	F11R	0,747	0,877
ENSOCUG00000001432	RARG	0,745	0,908
ENSOCUG00000002025	UBAC2	0,743	0,700
ENSOCUG00000004921	DCLRE1A	0,743	0,744
ENSOCUG00000013894	DERA	0,741	0,556
ENSOCUG00000001265	DCK	0,740	0,634
ENSOCUG00000006943	UNC5B	0,739	0,679
ENSOCUG00000012160	PRRC1	0,737	0,878
ENSOCUG00000026379	TRMT12	0,737	1,102
ENSOCUG00000016897	SLC22A17	0,736	0,600

ENSOCUG00000003662	PLXNA1	0,734	0,787
ENSOCUG00000001725	PDGFRB	0,731	0,765
ENSOCUG00000001732	TMEM184C	0,731	0,725
ENSOCUG00000000727	NECTIN3	0,730	0,542
ENSOCUG00000010476	FANCC	0,727	0,660
ENSOCUG00000002026	TBX18	0,726	0,750
ENSOCUG00000004042	PTPRF	0,725	0,881
ENSOCUG00000015482	TMEM241	0,723	0,696
ENSOCUG00000010360	HSPE1	0,722	0,477
ENSOCUG00000003401	SLC35B3	0,721	0,802
ENSOCUG00000006777	FAM102B	0,720	0,678
ENSOCUG00000024551	ATRAID	0,720	0,721
ENSOCUG00000001288	UBB	0,718	0,498
ENSOCUG00000008275	ANO10	0,718	0,601
ENSOCUG00000001517	BTF3L4	0,716	0,635
ENSOCUG00000012411	UHMK1	0,715	0,611
ENSOCUG00000016487	DDR1	0,714	0,921
ENSOCUG00000002011	EXTL2	0,711	0,798
ENSOCUG00000009444	ALDH1A1	0,711	0,640
ENSOCUG00000009838	FIGN	0,711	0,583
ENSOCUG00000006606	DCHS1	0,706	0,890
ENSOCUG00000011779	TNFRSF19	0,706	0,655
ENSOCUG00000028026	STAB1	0,706	0,830
ENSOCUG00000004299	SLF1	0,704	0,552
ENSOCUG00000017709	TRPC1	0,704	0,641
ENSOCUG00000010376	MGST3	0,701	0,539
ENSOCUG00000003860	PEX6	0,699	0,491
ENSOCUG00000013813	IL33	0,697	0,669
ENSOCUG00000005958	ATRNL1	0,697	0,635
ENSOCUG00000008364	PTPN4	0,696	0,638
ENSOCUG00000001979	SRR	0,694	0,630
ENSOCUG00000006453	NPR2	0,694	0,674
ENSOCUG00000004667	FUT8	0,693	0,464
ENSOCUG00000024309	SMAD7	0,691	0,935
ENSOCUG00000012416	STON2	0,691	0,547
ENSOCUG00000012682	CCNI	0,690	0,849
ENSOCUG00000002653	PIGO	0,689	0,692
ENSOCUG00000009000	RNF145	0,685	0,688
ENSOCUG00000008610	DPYD	0,681	0,610
ENSOCUG00000016989	CAD	0,680	0,589
ENSOCUG00000007056	TNXB	0,680	0,895
ENSOCUG00000014453	ACACA	0,679	0,499
ENSOCUG00000009849	GLT8D1	0,675	0,704
ENSOCUG00000001859	NUDT7	0,674	0,598
ENSOCUG00000014492	IRAK3	0,674	0,786

ENSOCUG00000005174	DPT	0,673	0,506
ENSOCUG00000000653	NDRG2	0,673	0,587
ENSOCUG00000000673	GEMIN2	0,673	0,586
ENSOCUG00000000696	SMTNL2	0,672	0,912
ENSOCUG00000004776	SERAC1	0,670	0,527
ENSOCUG00000006591	SCOC	0,670	0,803
ENSOCUG00000000791	RPL24	0,668	0,689
ENSOCUG00000012930	PPP2R1B	0,668	0,436
ENSOCUG00000014480	C1R	0,667	0,658
ENSOCUG00000003866	FBN1	0,666	0,709
ENSOCUG00000023727	LCAT	0,666	0,828
ENSOCUG00000022296	TAGLN2	0,665	0,987
ENSOCUG00000016501	SEC61A1	0,664	0,726
ENSOCUG00000003519	ARHGAP44	0,663	0,529
ENSOCUG00000016477	SELP	0,663	0,469
ENSOCUG00000005680	AMD1	0,663	0,638
ENSOCUG00000002828	IGFBP4	0,662	0,761
ENSOCUG00000015793	SERINC1	0,662	0,699
ENSOCUG00000007417	SGPP1	0,660	0,665
ENSOCUG00000007460	SOD3	0,659	0,921
ENSOCUG00000016292	TGFBR1	0,658	0,496
ENSOCUG00000006156	WDPCP	0,657	0,994
ENSOCUG00000011204	TIA1	0,654	0,620
ENSOCUG00000016600	GABARAPL2	0,654	0,440
ENSOCUG00000002519	ARMT1	0,653	0,679
ENSOCUG00000000127	GALNT1	0,652	0,509
ENSOCUG00000008261	NXT2	0,652	0,974
ENSOCUG00000005956	ALDH18A1	0,652	0,668
ENSOCUG00000006968	NEU1	0,651	0,575
ENSOCUG00000012575	PTAR1	0,649	0,599
ENSOCUG00000006658	BORA	0,649	0,860
ENSOCUG00000003220	GPR34	0,644	0,813
ENSOCUG00000009598	LTBP1	0,643	0,670
ENSOCUG00000016796	SPINT2	0,641	0,806
ENSOCUG00000004001	CNDP2	0,641	0,644
ENSOCUG00000003378	UNC50	0,640	0,454
ENSOCUG00000002137	UGP2	0,640	0,383
ENSOCUG00000014920	SPRY1	0,638	0,678
ENSOCUG00000014237	GK5	0,638	0,463
ENSOCUG00000000764	CMTM6	0,635	0,763
ENSOCUG00000021230	RNFT1	0,634	0,646
ENSOCUG00000002295	Naca	0,632	0,562
ENSOCUG00000008797	GOLT1B	0,631	0,604
ENSOCUG00000000867	MRPS28	0,631	0,452
ENSOCUG00000004334	TMEM255A	0,631	0,801

ENSOCUG00000013232	COL4A1	0,631	0,417
ENSOCUG00000010431	RAB3GAP2	0,629	0,559
ENSOCUG00000005251	INSIG2	0,627	0,637
ENSOCUG00000009775	TEX14	0,626	0,615
ENSOCUG00000000392	ARMC1	0,626	0,677
ENSOCUG00000026336	RSPS19	0,624	0,624
ENSOCUG00000015874	DCAF7	0,622	0,690
ENSOCUG00000004627	APH1B	0,621	0,887
ENSOCUG00000005964	TCTN3	0,621	0,546
ENSOCUG00000008204	SOAT1	0,620	0,589
ENSOCUG00000010317	MANF	0,619	0,365
ENSOCUG00000003766	DPYSL3	0,618	0,679
ENSOCUG00000011850	ATP6V0D1	0,615	0,540
ENSOCUG00000026333	PER1	0,615	0,941
ENSOCUG00000012845	SERTAD4	0,614	0,656
ENSOCUG00000021770	MFF	0,611	1,052
ENSOCUG00000015135	WDSUB1	0,611	0,871
ENSOCUG00000007893	GPAM	0,611	0,548
ENSOCUG00000017936	SLC39A10	0,611	0,697
ENSOCUG00000007070	ADAM15	0,610	0,612
ENSOCUG00000004199	CYP2J2	0,608	0,411
ENSOCUG00000007069	FGFR2	0,608	0,510
ENSOCUG00000003979	MANSC1	0,608	0,644
ENSOCUG00000000272	EMC1	0,607	0,696
ENSOCUG00000006668	KLHL15	0,599	0,569
ENSOCUG00000015427	DBF4	0,599	0,684
ENSOCUG00000000200	RAP1GAP2	0,598	0,498
ENSOCUG00000000857	FUT10	0,598	0,651
ENSOCUG00000002494	FBXO33	0,598	0,714
ENSOCUG00000029252	HFM1	0,596	0,563
ENSOCUG00000010086	ENO1	0,596	0,548
ENSOCUG00000026120	FOLH1	0,595	0,371
ENSOCUG00000013088	HAUS4	0,594	0,600
ENSOCUG00000012407	MID2	0,593	0,581
ENSOCUG00000010647	BZW1	0,593	0,576
ENSOCUG00000026383	Akr1b10	0,593	0,707
ENSOCUG00000013895	RAB5B	0,592	0,424
ENSOCUG00000006345	GNS	0,591	0,578
ENSOCUG00000010978	SHPRH	0,588	0,642
ENSOCUG00000000518	UHRF2	0,587	0,609
ENSOCUG00000006010	ARHGDIB	0,587	0,840
ENSOCUG00000011056	OSTC	0,585	0,567
ENSOCUG00000002150	RPS16	0,585	0,531
ENSOCUG00000001908	ATG7	0,585	0,566
ENSOCUG00000015882	EPDR1	0,583	0,583

ENSOCUG00000010695	BABAM2	0,582	0,481
ENSOCUG00000008124	MMADHC	0,581	0,397
ENSOCUG00000000049	MASP1	0,580	0,673
ENSOCUG00000000010	GNE	0,578	0,693
ENSOCUG00000025830	CAPN5	0,576	0,868
ENSOCUG00000005431	METTL21A	0,575	0,869
ENSOCUG00000002722	ANAPC5	0,574	0,669
ENSOCUG00000015851	DYM	0,573	0,293
ENSOCUG00000017670	PTBP3	0,573	0,711
ENSOCUG00000011941	HVCN1	0,572	0,699
ENSOCUG00000005642	MTPN	0,571	0,707
ENSOCUG00000003129	WDR36	0,568	0,650
ENSOCUG00000006444	CD109	0,568	0,604
ENSOCUG00000009946	AP3M1	0,568	0,662
ENSOCUG00000004928	TGFBI	0,567	0,880
ENSOCUG00000011317	GALNT11	0,565	0,526
ENSOCUG00000001412	IGFBP6	0,565	0,555
ENSOCUG00000006650	ETF1	0,565	0,445
ENSOCUG00000016994	TXNDC11	0,564	0,557
ENSOCUG00000002657	GNB2	0,563	0,936
ENSOCUG00000007990	OSBPL3	0,561	0,542
ENSOCUG00000010481	EGFR	0,561	0,424
ENSOCUG00000000203	MAGT1	0,560	0,592
ENSOCUG00000005265	NRIP1	0,560	0,259
ENSOCUG00000001887	TMEM245	0,559	0,535
ENSOCUG00000016565	NOTCH2	0,559	0,638
ENSOCUG00000017726	ITGA11	0,559	0,507
ENSOCUG00000016730	CXorf56	0,557	0,555
ENSOCUG00000007776	MMS19	0,555	0,600
ENSOCUG00000010620	TTC21B	0,555	0,547
ENSOCUG00000003027	TBRG1	0,550	0,636
ENSOCUG00000004616	MTMR11	0,550	0,873
ENSOCUG00000008635	MAN1C1	0,549	0,711
ENSOCUG00000003414	IFT88	0,549	0,675
ENSOCUG00000004862	IPO11	0,546	0,535
ENSOCUG00000009483	TMEM39A	0,545	0,749
ENSOCUG00000016001	PPA1	0,545	0,410
ENSOCUG00000007237	HSD17B11	0,545	0,570
ENSOCUG00000021110	UBE3D	0,543	0,604
ENSOCUG00000014137	NAALAD2	0,543	0,665
ENSOCUG00000013901	IMPACT	0,543	0,449
ENSOCUG00000022399	RPL13	0,542	0,29
ENSOCUG00000003181	ZYG11B	0,542	0,286
ENSOCUG00000012915	SIK2	0,542	0,408
ENSOCUG00000013276	COL4A2	0,541	0,645

ENSOCUG00000016056	SGSM2	0,541	0,426
ENSOCUG00000014780	KLHL7	0,540	0,707
ENSOCUG00000001343	IQSEC1	0,539	0,665
ENSOCUG00000000608	PTGFRN	0,538	0,617
ENSOCUG00000001256	GANAB	0,537	0,511
ENSOCUG00000015979	DPY19L1	0,537	0,582
ENSOCUG00000013769	SH3PXD2A	0,536	0,569
ENSOCUG00000007582	RNF11	0,534	0,573
ENSOCUG00000003348	ATP5S	0,533	0,379
ENSOCUG00000006502	SEPT10	0,533	0,418
ENSOCUG00000011201	CYP4B1	0,531	0,730
ENSOCUG00000004990	APEH	0,529	0,594
ENSOCUG00000000763	PLA2G7	0,528	0,561
ENSOCUG00000015052	XRCC4	0,528	0,560
ENSOCUG00000001874	ATR	0,528	0,482
ENSOCUG00000009419	APPBP2	0,528	0,555
ENSOCUG00000013811	SCYL3	0,526	0,494
ENSOCUG00000004731	RBM14-RBM4	0,526	0,681
ENSOCUG00000005295	GARS	0,525	0,476
ENSOCUG00000004258	METAP1	0,525	0,638
ENSOCUG00000007519	CDK7	0,524	0,471
ENSOCUG00000010801	PPEF1	0,523	0,709
ENSOCUG00000008426	GGA2	0,522	0,511
ENSOCUG00000014143	INTS6L	0,522	0,649
ENSOCUG00000002102	GTDC1	0,522	0,467
ENSOCUG00000009165	TTI1	0,521	0,611
ENSOCUG00000014174	ZNF395	0,521	0,743
ENSOCUG00000003711	ELF2	0,521	0,435
ENSOCUG00000000729	TM9SF2	0,519	0,546
ENSOCUG00000017117	B2M	0,519	0,854
ENSOCUG00000005737	LGMN	0,519	0,761
ENSOCUG00000010408	BPNT1	0,519	0,421
ENSOCUG00000009711	CRIPT	0,519	0,762
ENSOCUG00000006802	LCORL	0,517	0,363
ENSOCUG00000001817	MED23	0,517	0,537
ENSOCUG00000014086	RFX3	0,516	0,673
ENSOCUG00000004643	WSB1	0,516	0,701
ENSOCUG00000024465	COL8A1	0,516	0,497
ENSOCUG00000025433	CRY2	0,515	0,552
ENSOCUG00000003369	APOOL	0,515	0,440
ENSOCUG00000006408	DISP1	0,514	0,595
ENSOCUG00000016154	KCTD20	0,514	0,531
ENSOCUG00000001232	RPL35A	0,514	0,674
ENSOCUG00000007901	SEMA6D	0,514	0,737
ENSOCUG00000028126	YIPF5	0,512	0,598

ENSOCUG00000005908	SLC35A5	0,512	0,567
ENSOCUG00000010732	CYFIP1	0,512	0,602
ENSOCUG00000013126	TRPC4AP	0,511	0,610
ENSOCUG00000010700	URB2	0,511	0,476
ENSOCUG00000008774	PTEN	0,509	0,540
ENSOCUG00000008481	EEF1A1	0,507	0,587
ENSOCUG00000016126	PRMT2	0,506	0,575
ENSOCUG00000013167	SLC9A6	0,506	0,441
ENSOCUG00000015255	EIF5A	0,505	0,464
ENSOCUG00000010375	EIF4A1	0,504	0,428
ENSOCUG00000004153	ICE2	0,504	0,388
ENSOCUG00000008734	TRAM1	0,503	0,596
ENSOCUG00000004107	ST3GAL2	0,503	0,839
ENSOCUG00000026606	SCARNA2	0,503	0,421
ENSOCUG00000010371	FRZB	0,502	0,587
ENSOCUG00000005491	CDKN2AIP	0,502	0,745
ENSOCUG00000012254	SNAI2	0,501	0,673
ENSOCUG00000007580	RAB30	0,501	0,515
ENSOCUG00000009234	USP45	0,500	0,479
ENSOCUG00000015652	RECK	0,500	0,477
ENSOCUG00000007206	ANKRD52	0,499	0,926
ENSOCUG00000000432	COPB1	0,499	0,528
ENSOCUG00000014515	CAV2	0,499	0,253
ENSOCUG00000016221	GALNT12	0,499	0,691
ENSOCUG00000013022	RNASE4	0,498	0,536
ENSOCUG00000009925	SEC23A	0,498	0,408
ENSOCUG00000007907	OCIAD2	0,498	0,424
ENSOCUG00000017254	BBS2	0,498	0,462
ENSOCUG00000000231	AASDHPPT	0,497	0,438
ENSOCUG00000001048	THADA	0,497	0,626
ENSOCUG00000012120	SPARC	0,496	0,551
ENSOCUG00000017336	CLCN5	0,495	0,499
ENSOCUG00000017172	HIST4H4	0,492	0,515
ENSOCUG00000005082	QARS	0,491	0,487
ENSOCUG00000008556	NAB1	0,491	0,603
ENSOCUG00000000076	SLC43A2	0,490	0,618
ENSOCUG00000015538	SLC35A3	0,488	0,437
ENSOCUG00000003208	DOCK8	0,488	0,556
ENSOCUG00000008111	LRRC42	0,488	0,518
ENSOCUG00000005734	UTP4	0,487	0,460
ENSOCUG00000011955	SIDT2	0,486	0,621
ENSOCUG00000006059	PFN2	0,485	0,564
ENSOCUG00000017212	AMFR	0,484	0,560
ENSOCUG00000016563	DYNLL2	0,484	0,362
ENSOCUG00000014672	GNG2	0,482	0,600

ENSOCUG00000002610	UBE2L3	0,480	0,529
ENSOCUG00000012192	AASDH	0,480	0,402
ENSOCUG00000011509	RPS14	0,479	0,581
ENSOCUG00000000489	TTC13	0,479	0,439
ENSOCUG00000016381	BROX	0,478	0,410
ENSOCUG00000000907	STK35	0,478	0,535
ENSOCUG00000005471	PREPL	0,478	0,496
ENSOCUG00000014088	ETNK1	0,477	0,595
ENSOCUG00000012468	ASNSD1	0,477	0,758
ENSOCUG00000001542	RIPK2	0,476	0,512
ENSOCUG00000012011	TRAF6	0,476	0,519
ENSOCUG00000010069	RNF41	0,474	0,473
ENSOCUG00000013596	SLC4A7	0,473	0,293
ENSOCUG00000017812	TCF12	0,473	0,566
ENSOCUG00000001418	PSMD14	0,473	0,379
ENSOCUG00000010989	FBXL20	0,472	0,510
ENSOCUG00000011764	LPP	0,472	0,428
ENSOCUG00000011453	PPIH	0,471	0,415
ENSOCUG00000009152	RFWD3	0,471	0,561
ENSOCUG00000009949	CD200	0,470	0,434
ENSOCUG00000016963	GNPDA1	0,470	0,429
ENSOCUG00000011355	SCPEP1	0,469	0,478
ENSOCUG00000016965	OGT	0,469	0,446
ENSOCUG00000000520	CNIH4	0,468	0,472
ENSOCUG00000015354	ITCH	0,467	0,288
ENSOCUG00000007784	TMEM199	0,467	0,395
ENSOCUG00000017089	SNX17	0,466	0,658
ENSOCUG00000004669	TMED10	0,466	0,355
ENSOCUG00000006352	FOXRED2	0,465	0,593
ENSOCUG00000002199	GATA4	0,465	0,618
ENSOCUG00000022874	RPL7A	0,465	0,445
ENSOCUG00000011515	NDST1	0,464	0,612
ENSOCUG00000003142	AR	0,463	0,842
ENSOCUG00000000368	THAP6	0,462	0,347
ENSOCUG00000006714	MMP2	0,462	0,603
ENSOCUG00000000566	XPR1	0,462	0,356
ENSOCUG00000025635	CAMKK2	0,462	0,896
ENSOCUG00000003377	CYP27A1	0,461	0,512
ENSOCUG00000003778	AP4B1	0,461	0,440
ENSOCUG00000012620	WRN	0,460	0,371
ENSOCUG00000024409	MON1B	0,460	0,555
ENSOCUG00000027435	RPS11	0,459	0,549
ENSOCUG00000004827	SFXN1	0,458	0,417
ENSOCUG00000008704	SPATA6	0,457	0,494
ENSOCUG00000013689	LAPTM4B	0,457	0,634

ENSOCUG00000009774	LAPTM5	0,457	0,488
ENSOCUG00000001486	ZNF639	0,456	0,377
ENSOCUG00000010638	INTS4	0,455	0,575
ENSOCUG00000016739	UBE2A	0,454	0,359
ENSOCUG00000003196	CLIC4	0,454	0,370
ENSOCUG00000012528	ST3GAL4	0,453	0,741
ENSOCUG00000010280	PGRMC1	0,453	0,489
ENSOCUG00000002751	SFXN3	0,453	0,536
ENSOCUG00000012017	CHSY1	0,452	0,395
ENSOCUG00000005533	SCN2A	0,451	0,456
ENSOCUG00000010080	LRRC36	0,450	0,482
ENSOCUG00000023595	NDFIP1	0,450	0,389
ENSOCUG00000012610	CDON	0,450	0,729
ENSOCUG00000009521	TCEAL8	0,449	0,370
ENSOCUG00000008604	RAB31	0,448	0,477
ENSOCUG00000015528	C16orf72	0,448	0,402
ENSOCUG00000003648	XPNPEP1	0,448	0,580
ENSOCUG00000016729	PRIM2	0,447	0,559
ENSOCUG00000003504	SLFN13	0,445	0,445
ENSOCUG00000000807	PLEKHA8	0,444	0,419
ENSOCUG00000003333	LIFR	0,444	0,492
ENSOCUG00000017377	MATN2	0,443	0,841
ENSOCUG00000017959	PIGV	0,442	0,770
ENSOCUG00000004130	COG4	0,442	0,287
ENSOCUG00000024795	MNDA	0,441	0,434
ENSOCUG00000007744	CTNNA1	0,441	0,332
ENSOCUG00000014107	AREL1	0,439	0,281
ENSOCUG00000007022	PHF6	0,439	0,552
ENSOCUG00000000649	SLC36A4	0,437	0,339
ENSOCUG00000016136	SPG21	0,436	0,396
ENSOCUG00000015443	ADCY3	0,436	0,431
ENSOCUG00000010569	ERBB2	0,435	0,649
ENSOCUG00000011443	DDR2	0,435	0,409
ENSOCUG00000002312	TEP1	0,434	0,406
ENSOCUG00000026068	LDLRAD3	0,431	0,616
ENSOCUG00000013782	PHKA2	0,431	0,615
ENSOCUG00000008388	CCNG2	0,431	0,554
ENSOCUG00000003313	LOX	0,430	0,487
ENSOCUG00000008071	CST3	0,429	0,619
ENSOCUG00000004811	DERL2	0,428	0,496
ENSOCUG00000013049	PLXND1	0,427	0,385
ENSOCUG00000016199	BCL10	0,426	0,372
ENSOCUG00000000498	TVP23B	0,423	0,407
ENSOCUG00000004488	GMFB	0,423	0,333
ENSOCUG00000006041	PLS3	0,422	0,378

ENSOCUG00000017961	CPNE3	0,422	0,684
ENSOCUG00000015020	COL5A2	0,422	0,542
ENSOCUG00000009778	ANKRD46	0,421	0,436
ENSOCUG00000014711	CAPZB	0,421	0,262
ENSOCUG00000010999	LRRC57	0,420	0,654
ENSOCUG00000000345	C8orf46	0,420	0,698
ENSOCUG00000013478	HTRA1	0,420	0,352
ENSOCUG00000005881	IPO13	0,420	0,329
ENSOCUG00000011934	EPHX1	0,418	0,662
ENSOCUG00000009743	MTMR4	0,418	0,267
ENSOCUG00000027117	LRRC41	0,417	0,628
ENSOCUG00000009236	FAM135A	0,416	0,429
ENSOCUG00000006507	FUCA1	0,416	0,568
ENSOCUG00000008622	TRAPPC9	0,416	0,336
ENSOCUG00000004337	SMARCD2	0,416	0,615
ENSOCUG00000003607	AIG1	0,416	0,497
ENSOCUG00000016677	AEBP2	0,415	0,453
ENSOCUG00000001798	ACAP2	0,414	0,455
ENSOCUG00000026735	PPME1	0,413	0,404
ENSOCUG00000010955	HSPG2	0,412	0,500
ENSOCUG00000016413	CLTC	0,412	0,424
ENSOCUG00000010669	DSEL	0,412	0,368
ENSOCUG00000007681	FAM214A	0,412	0,295
ENSOCUG00000002042	NAALADL2	0,411	0,444
ENSOCUG00000016176	REEP3	0,411	0,471
ENSOCUG00000003028	STN1	0,411	0,476
ENSOCUG00000016722	RAPH1	0,411	0,448
ENSOCUG00000009927	HERC4	0,410	0,288
ENSOCUG00000011223	PLRG1	0,409	0,348
ENSOCUG00000004103	IRAK4	0,409	0,383
ENSOCUG00000005717	GCN1	0,408	0,422
ENSOCUG00000012843	CERS5	0,406	0,518
ENSOCUG00000001425	VIPAS39	0,406	0,448
ENSOCUG00000006317	SPG11	0,405	0,504
ENSOCUG00000007019	SKIV2L	0,405	0,855
ENSOCUG00000005292	TRIM2	0,404	0,461
ENSOCUG00000014843	ANKFY1	0,402	0,402
ENSOCUG00000012508	AVL9	0,401	0,328
ENSOCUG00000015127	SYPL1	0,401	0,240
ENSOCUG00000010426	AGFG1	0,399	0,258
ENSOCUG00000006604	TPP1	0,398	0,299
ENSOCUG00000004233	INVS	0,398	0,449
ENSOCUG00000022412	RPS3	0,398	0,456
ENSOCUG00000004483	EIF3M	0,397	0,388
ENSOCUG00000013853	NCBP1	0,396	0,292

ENSOCUG00000010653	CLK1	0,395	0,427
ENSOCUG00000024126	DDX39B	0,395	0,504
ENSOCUG00000006924	FAM171A1	0,393	0,609
ENSOCUG00000013101	PARVA	0,393	0,371
ENSOCUG00000013114	CHUK	0,392	0,301
ENSOCUG00000014589	CNOT7	0,392	0,263
ENSOCUG00000000824	ARF4	0,391	0,512
ENSOCUG00000008982	BLZF1	0,391	0,365
ENSOCUG00000008764	TGOLN2	0,391	0,408
ENSOCUG00000017608	PTBP2	0,389	0,476
ENSOCUG00000011187	COPS7A	0,389	0,320
ENSOCUG00000026332	SERF2	0,389	0,386
ENSOCUG00000013185	WDR35	0,388	0,625
ENSOCUG00000027397	Uox	0,387	0,496
ENSOCUG00000013319	TCTA	0,386	0,484
ENSOCUG00000000409	COL6A2	0,385	0,460
ENSOCUG00000027987	CSPG4	0,385	0,327
ENSOCUG00000002074	RPL29	0,384	0,381
ENSOCUG00000002727	XPO6	0,383	0,513
ENSOCUG00000017060	GTF3C2	0,383	0,265
ENSOCUG00000013696	BICC1	0,383	0,482
ENSOCUG00000015837	GM2A	0,382	0,436
ENSOCUG00000011074	TMEM43	0,381	0,557
ENSOCUG00000012051	ANKH	0,380	0,377
ENSOCUG00000013510	DCTN4	0,379	0,314
ENSOCUG00000029156	PRELID2	0,378	0,574
ENSOCUG00000010058	ZBTB44	0,378	0,307
ENSOCUG00000027241	MFAP3	0,378	0,420
ENSOCUG00000008660	SRPK1	0,378	0,294
ENSOCUG00000010649	MKX	0,377	0,505
ENSOCUG00000003252	MYNN	0,375	0,296
ENSOCUG00000017760	ANXA5	0,375	0,432
ENSOCUG00000003507	HAUS6	0,375	0,357
ENSOCUG00000008003	KDM5B	0,375	0,345
ENSOCUG00000007344	DSE	0,373	0,450
ENSOCUG00000015267	GPX8	0,371	0,439
ENSOCUG00000011423	LMBRD2	0,371	0,377
ENSOCUG00000026209	NPAT	0,371	0,452
ENSOCUG00000000931	GNB4	0,371	0,397
ENSOCUG00000012771	COG5	0,371	0,229
ENSOCUG00000014970	ACSL3	0,368	0,303
ENSOCUG00000008289	FBXO7	0,366	0,363
ENSOCUG00000015088	RETREG2	0,365	0,536
ENSOCUG00000003553	SH3GLB1	0,364	0,471
ENSOCUG00000005531	UGDH	0,363	0,275

ENSOCUG00000016083	TGFBR3	0,362	0,475
ENSOCUG00000005373	MFSD14B	0,360	0,312
ENSOCUG00000000847	PTPRD	0,360	0,419
ENSOCUG00000003245	GNB2L1	0,359	0,441
ENSOCUG00000014741	KDM6A	0,358	0,386
ENSOCUG00000008567	NF1	0,358	0,384
ENSOCUG00000002432	KDELC1	0,355	0,355
ENSOCUG00000017522	TGS1	0,355	0,346
ENSOCUG00000006944	SPOPL	0,355	0,349
ENSOCUG00000001461	PLOD2	0,355	0,553
ENSOCUG00000000102	BBS9	0,354	0,353
ENSOCUG00000003451	CCNL1	0,353	0,455
ENSOCUG00000000956	ZFYVE9	0,352	0,317
ENSOCUG00000017399	RNF139	0,352	0,373
ENSOCUG00000007383	CCNT1	0,352	0,343
ENSOCUG00000007150	VPS4B	0,350	0,395
ENSOCUG00000005144	STXBP6	0,349	0,389
ENSOCUG00000013890	BIRC2	0,349	0,413
ENSOCUG00000012948	DOCK7	0,348	0,450
ENSOCUG00000014552	TIPRL	0,348	0,276
ENSOCUG00000014007	HEATR1	0,347	0,292
ENSOCUG00000016298	ATP6V1H	0,347	0,336
ENSOCUG00000025834	YAP1	0,347	0,493
ENSOCUG00000000750	PRMT3	0,347	0,381
ENSOCUG00000012309	SGCE	0,346	0,636
ENSOCUG00000002991	CLCC1	0,345	0,334
ENSOCUG00000010702	NUMB	0,345	0,335
ENSOCUG00000007477	IPO9	0,344	0,461
ENSOCUG00000002884	JAG1	0,343	0,326
ENSOCUG00000003710	SPPL2A	0,342	0,314
ENSOCUG00000007228	HEATR5A	0,342	0,359
ENSOCUG00000017086	ZMYM2	0,341	0,364
ENSOCUG00000001311	CELF1	0,341	0,221
ENSOCUG00000011184	TSPAN6	0,341	0,430
ENSOCUG00000029552	RAP1B	0,339	0,360
ENSOCUG00000006277	BNIP2	0,339	0,295
ENSOCUG00000004412	AP1G1	0,339	0,447
ENSOCUG00000002512	WDR7	0,339	0,282
ENSOCUG00000009492	POGLUT1	0,337	0,373
ENSOCUG00000010631	PIK3CA	0,335	0,336
ENSOCUG00000003783	TIAL1	0,334	0,250
ENSOCUG00000016750	SLC41A2	0,334	0,296
ENSOCUG00000010609	DUSP16	0,334	0,446
ENSOCUG00000008195	ZNF207	0,334	0,259
ENSOCUG00000013692	MAPK1	0,333	0,421

ENSOCUG00000011896	FBXL3	0,333	0,372
ENSOCUG00000010815	CDC27	0,333	0,306
ENSOCUG00000000081	PRPF8	0,332	0,350
ENSOCUG00000008403	USP11	0,331	0,404
ENSOCUG00000029416	MAPK14	0,331	0,361
ENSOCUG00000010827	BOC	0,331	0,553
ENSOCUG00000015532	CACHD1	0,331	0,769
ENSOCUG00000023323	LYSMD3	0,330	0,290
ENSOCUG00000013065	KLHL24	0,330	0,261
ENSOCUG00000015785	WASHC5	0,330	0,459
ENSOCUG00000004576	ANKIB1	0,329	0,279
ENSOCUG00000015292	NEDD9	0,327	0,546
ENSOCUG00000007590	SUCO	0,326	0,311
ENSOCUG00000017291	FBXO38	0,325	0,291
ENSOCUG00000014663	ATM	0,325	0,380
ENSOCUG00000012352	DCUN1D4	0,324	0,336
ENSOCUG00000001191	LYRM1	0,323	0,268
ENSOCUG00000001439	FAT1	0,323	0,397
ENSOCUG00000004986	XPO1	0,323	0,527
ENSOCUG00000013953	RPS6KC1	0,323	0,294
ENSOCUG00000007361	EXT2	0,322	0,479
ENSOCUG00000009294	EFR3A	0,322	0,414
ENSOCUG00000023809	PICALM	0,321	0,287
ENSOCUG00000014775	USP3	0,321	0,382
ENSOCUG00000010731	ARHGEF11	0,320	0,397
ENSOCUG00000017003	RAB1A	0,317	0,270
ENSOCUG00000016016	AKAP11	0,316	0,282
ENSOCUG00000004225	EIF3F	0,316	0,565
ENSOCUG00000003929	SMAD4	0,316	0,307
ENSOCUG00000012117	LTN1	0,316	0,316
ENSOCUG00000015742	CEPT1	0,314	0,501
ENSOCUG00000017908	PUM2	0,314	0,300
ENSOCUG00000013639	ADGRL2	0,314	0,291
ENSOCUG00000003404	MAN2A1	0,312	0,400
ENSOCUG00000001554	TANC2	0,311	0,559
ENSOCUG00000023994	CPNE1	0,311	0,577
ENSOCUG00000005332	CDC14B	0,310	0,384
ENSOCUG00000002065	TCAF1	0,310	0,405
ENSOCUG00000000625	VPS50	0,309	0,370
ENSOCUG00000016002	DPYSL2	0,309	0,499
ENSOCUG00000005831	RPN2	0,308	0,281
ENSOCUG00000024483	PTGES3	0,307	0,481
ENSOCUG00000013272	SEC24D	0,306	0,304
ENSOCUG00000006593	ILK	0,305	0,249
ENSOCUG00000001142	SNX13	0,303	0,278

ENSOCUG00000017001	KANSL1L	0,303	0,347
ENSOCUG00000000758	SLC25A27	0,303	0,304
ENSOCUG00000027658	ERGIC3	0,302	0,434
ENSOCUG00000008716	NBEAL1	0,302	0,337
ENSOCUG00000014677	STT3A	0,302	0,226
ENSOCUG00000004871	RARS2	0,301	0,271
ENSOCUG00000012455	MDFIC	0,301	0,330
ENSOCUG00000013495	TM9SF4	0,301	0,361
ENSOCUG00000001468	CTNNB1	0,299	0,178
ENSOCUG00000012692	TMPO	0,299	0,316
ENSOCUG00000002448	GLS	0,297	0,512
ENSOCUG00000002100	PLEKHA1	0,296	0,249
ENSOCUG00000005291	KIAA1468	0,296	0,336
ENSOCUG00000025925	HNRNPK	0,295	0,263
ENSOCUG00000027209	ST6GAL1	0,294	0,677
ENSOCUG00000001784	DNAJC10	0,293	0,19
ENSOCUG00000003388	SCARB2	0,292	0,358
ENSOCUG00000022505	SKP1	0,291	0,229
ENSOCUG00000002439	ACVR1	0,290	0,564
ENSOCUG00000013212	ACLY	0,290	0,344
ENSOCUG00000010432	MBD5	0,290	0,361
ENSOCUG00000005327	TFG	0,287	0,246
ENSOCUG00000013263	FAM208A	0,285	0,302
ENSOCUG00000001578	CUL7	0,284	0,554
ENSOCUG00000014363	PCNX2	0,283	0,292
ENSOCUG00000003759	RSBN1	0,283	0,304
ENSOCUG00000027465	ICK	0,283	0,371
ENSOCUG00000000170	NUP155	0,283	0,278
ENSOCUG00000029667	CUBN	0,282	0,348
ENSOCUG00000010401	ZDHHC17	0,280	0,368
ENSOCUG00000006900	PIAS2	0,279	0,222
ENSOCUG00000014520	CAV1	0,276	0,158
ENSOCUG00000008455	SSR2	0,276	0,298
ENSOCUG00000002669	TCF4	0,273	0,216
ENSOCUG00000002975	STXBP3	0,273	0,211
ENSOCUG00000014277	ABHD4	0,273	0,409
ENSOCUG00000012633	MORC4	0,273	0,407
ENSOCUG00000014160	ATP7A	0,272	0,405
ENSOCUG00000005209	ZNF644	0,270	0,253
ENSOCUG00000002924	LGALS3	0,270	0,270
ENSOCUG00000016580	CTSB	0,268	0,442
ENSOCUG00000017078	TBL1XR1	0,268	0,342
ENSOCUG00000005030	WASHC4	0,268	0,254
ENSOCUG00000016117	AMBRA1	0,266	0,455
ENSOCUG00000009227	CDK14	0,266	0,286

ENSOCUG00000026080	SACM1L	0,265	0,439
ENSOCUG00000005403	SLC30A6	0,265	0,337
ENSOCUG00000016010	LMBRD1	0,265	0,236
ENSOCUG00000014819	ZZEF1	0,265	0,225
ENSOCUG00000012200	RAB5A	0,264	0,206
ENSOCUG00000017283	SMARCAD1	0,262	0,254
ENSOCUG00000014213	CTNND1	0,262	0,229
ENSOCUG00000005266	SENP7	0,262	0,359
ENSOCUG00000022530	ACVRL1	0,260	0,323
ENSOCUG00000011293	YME1L1	0,259	0,185
ENSOCUG00000001420	SYNJ1	0,259	0,329
ENSOCUG00000011302	RPL10	0,258	0,277
ENSOCUG00000003320	DENND4C	0,258	0,326
ENSOCUG00000005092	USP19	0,258	0,460
ENSOCUG00000008421	KDM3B	0,257	0,263
ENSOCUG00000012292	CASD1	0,257	0,367
ENSOCUG00000007601	MBNL1	0,256	0,262
ENSOCUG00000017155	WDFY3	0,256	0,299
ENSOCUG00000012937	PRKACB	0,255	0,307
ENSOCUG00000006378	TOGARAM1	0,255	0,334
ENSOCUG00000006669	CSNK1A1	0,255	0,269
ENSOCUG00000017837	SESTD1	0,253	0,326
ENSOCUG00000003950	EPG5	0,252	0,465
ENSOCUG00000005948	DMXL2	0,251	0,464
ENSOCUG00000017386	RPL30	0,250	0,297
ENSOCUG00000006684	SRSF3	0,249	0,291
ENSOCUG00000006207	BTBD7	0,248	0,213
ENSOCUG00000006354	RBL2	0,247	0,270
ENSOCUG00000006911	CNOT1	0,247	0,259
ENSOCUG00000003338	RNF111	0,246	0,214
ENSOCUG00000002445	HP1BP3	0,246	0,190
ENSOCUG00000008929	SNX14	0,244	0,254
ENSOCUG00000003802	RPL6	0,241	0,282
ENSOCUG00000016281	MKLN1	0,241	0,296
ENSOCUG00000010777	DOPEY1	0,236	0,230
ENSOCUG00000006600	BIRC6	0,236	0,221
ENSOCUG00000008050	EIF3E	0,234	0,282
ENSOCUG00000016495	VIRMA	0,234	0,286
ENSOCUG00000011549	SPATS2L	0,231	0,260
ENSOCUG00000015169	ATP6V0A1	0,231	0,331
ENSOCUG00000002148	OSBP	0,230	0,216
ENSOCUG00000026003	RBBP4	0,228	0,227
ENSOCUG00000017012	ZC3H7A	0,228	0,269
ENSOCUG00000005304	RPS17	0,228	0,210
ENSOCUG00000017056	PKN2	0,228	0,191

ENSOCUG00000012757	NUP205	0,228	0,266
ENSOCUG00000010232	ANTXR1	0,228	0,396
ENSOCUG00000016551	C1QTNF7	0,226	0,247
ENSOCUG00000012525	KBTBD2	0,225	0,222
ENSOCUG00000001480	GMPS	0,225	0,237
ENSOCUG00000013806	SORT1	0,224	0,190
ENSOCUG00000016004	ANAPC4	0,222	0,233
ENSOCUG00000004906	ACTR2	0,219	0,206
ENSOCUG00000016925	BMPR1A	0,213	0,288
ENSOCUG00000007198	USP9X	0,213	0,148
ENSOCUG00000017371	MYL6	0,212	0,295
ENSOCUG00000007534	EIF3H	0,212	0,279
ENSOCUG00000002187	TUBB	0,211	0,264
ENSOCUG00000013912	WDR11	0,208	0,250
ENSOCUG00000011880	SPIN1	0,199	0,345
ENSOCUG00000006616	EFCAB14	0,197	0,271
ENSOCUG00000002867	LYST	0,195	0,203
ENSOCUG00000009010	PI4KA	0,195	0,258
ENSOCUG00000002775	NSD1	0,193	0,164
ENSOCUG00000005111	N4BP2	0,193	0,384
ENSOCUG00000006807	RALGAPA1	0,192	0,297
ENSOCUG00000003520	HLTF	0,189	0,242
ENSOCUG00000000051	DMXL1	0,189	0,167
ENSOCUG00000017163	C2CD5	0,187	0,157
ENSOCUG00000010548	ARHGAP5	0,184	0,207
ENSOCUG00000010203	TLN1	0,182	0,298
ENSOCUG00000002676	PIKFYVE	0,182	0,272
ENSOCUG00000012331	UBR1	0,182	0,242
ENSOCUG00000016257	DYNC2H1	0,182	0,290
ENSOCUG00000013133	NCKAP1	0,176	0,172
ENSOCUG00000005741	MARCH6	0,171	0,232
ENSOCUG00000008928	SMU1	0,170	0,243
ENSOCUG00000017419	PRKAR1A	0,169	0,148
ENSOCUG00000006709	MED13	0,167	0,169
ENSOCUG00000000996	CLASP2	0,163	0,126
ENSOCUG00000022490	PCBP2	0,162	0,234
ENSOCUG00000000264	UBR4	0,158	0,119
ENSOCUG00000017435	RIF1	-0,15	-0,156
ENSOCUG00000010460	NCOR1	-0,15	-0,193
ENSOCUG00000015381	QSER1	-0,16	-0,167
ENSOCUG00000001839	PPP2R3A	-0,16	-0,272
ENSOCUG00000002855	ATXN2	-0,16	-0,207
ENSOCUG00000016401	PHIP	-0,16	-0,146
ENSOCUG00000015080	UTRN	-0,17	-0,238
ENSOCUG00000025405	GDI2	-0,17	-0,231

ENSOCUG00000006754	HERC2	-0,17	-0,195
ENSOCUG00000005196	ARHGEF12	-0,18	-0,260
ENSOCUG00000000677	MACF1	-0,18	-0,182
ENSOCUG00000006443	SECISBP2L	-0,18	-0,253
ENSOCUG00000006190	NCAM1	-0,18	-0,248
ENSOCUG00000003899	SCAF11	-0,18	-0,304
ENSOCUG00000008438	SRRM1	-0,19	-0,207
ENSOCUG00000001215	HIPK3	-0,19	-0,362
ENSOCUG00000005996	THOC2	-0,19	-0,192
ENSOCUG00000007354	HUWE1	-0,19	-0,271
ENSOCUG00000007293	CLPX	-0,19	-0,387
ENSOCUG00000006686	HNRNPR	-0,19	-0,182
ENSOCUG00000014074	DST	-0,20	-0,312
ENSOCUG00000005008	SPTBN1	-0,20	-0,218
ENSOCUG00000021376	PTPRA	-0,20	-0,226
ENSOCUG00000000462	OXR1	-0,20	-0,299
ENSOCUG00000002768	MGEA5	-0,20	-0,224
ENSOCUG00000014615	MSH6	-0,21	-0,253
ENSOCUG00000008760	MTX2	-0,21	-0,236
ENSOCUG00000010288	TMEM106B	-0,21	-0,190
ENSOCUG00000024887	CAPRIN1	-0,21	-0,179
ENSOCUG00000011595	USP14	-0,21	-0,437
ENSOCUG00000017331	NAA15	-0,21	-0,288
ENSOCUG00000016430	SEC63	-0,22	-0,198
ENSOCUG00000009717	SNW1	-0,22	-0,227
ENSOCUG00000012730	TAX1BP1	-0,22	-0,433
ENSOCUG00000013323	RHOA	-0,22	-0,240
ENSOCUG00000012707	ELOA	-0,22	-0,246
ENSOCUG00000003686	USP8	-0,22	-0,268
ENSOCUG00000004996	EIF3A	-0,22	-0,369
ENSOCUG00000009371	RMND5A	-0,22	-0,197
ENSOCUG00000010051	APLP2	-0,22	-0,264
ENSOCUG00000009082	VDAC2	-0,22	-0,566
ENSOCUG00000022489	EIF3C	-0,22	-0,205
ENSOCUG00000009614	PHACTR2	-0,23	-0,292
ENSOCUG00000005461	PPM1B	-0,23	-0,221
ENSOCUG00000029173	RNF103	-0,23	-0,212
ENSOCUG00000016601	SSH2	-0,23	-0,375
ENSOCUG00000006997	GABARAP	-0,23	-0,192
ENSOCUG00000006794	OPTN	-0,23	-0,361
ENSOCUG00000005079	CEP76	-0,23	-0,445
ENSOCUG00000006262	SUB1	-0,23	-0,331
ENSOCUG00000013255	DNAJC7	-0,23	-0,400
ENSOCUG00000015867	ARFGEF1	-0,23	-0,301
ENSOCUG00000009955	PPM1A	-0,23	-0,381

ENSOCUG00000013550	USF3	-0,23	-0,344
ENSOCUG00000005888	NIPBL	-0,23	-0,203
ENSOCUG00000011765	CC2D2A	-0,23	-0,331
ENSOCUG00000021848	NONO	-0,23	-0,176
ENSOCUG00000017366	R3HDM1	-0,23	-0,375
ENSOCUG00000000817	SPAG9	-0,24	-0,244
ENSOCUG00000017880	EMSY	-0,24	-0,254
ENSOCUG00000001971	BMPR2	-0,24	-0,164
ENSOCUG00000016723	SPOP	-0,24	-0,295
ENSOCUG00000005362	CASC4	-0,24	-0,190
ENSOCUG00000002862	RPS15	-0,24	-0,240
ENSOCUG00000002739	PAIP1	-0,24	-0,442
ENSOCUG00000003228	CLK4	-0,24	-0,349
ENSOCUG00000004185	FNDC3B	-0,24	-0,184
ENSOCUG00000017384	NEMF	-0,24	-0,216
ENSOCUG00000012424	CMPK1	-0,24	-0,404
ENSOCUG00000000481	EIF2S2	-0,25	-0,568
ENSOCUG00000007093	IPO7	-0,25	-0,445
ENSOCUG00000010034	CHD7	-0,25	-0,432
ENSOCUG00000000991	HIF1AN	-0,25	-0,456
ENSOCUG00000013831	TMOD1	-0,25	-0,447
ENSOCUG00000013922	PSMD12	-0,25	-0,361
ENSOCUG00000007579	ATRX	-0,25	-0,266
ENSOCUG00000008167	CALU	-0,25	-0,273
ENSOCUG00000025313	PSMD6	-0,25	-0,448
ENSOCUG00000004759	GSPT1	-0,25	-0,237
ENSOCUG00000003627	GPSM2	-0,25	-0,297
ENSOCUG00000010029	GRSF1	-0,25	-0,386
ENSOCUG00000027344	CSF2RB	-0,25	-0,235
ENSOCUG00000013706	KIF16B	-0,25	-0,267
ENSOCUG00000005543	ZNF292	-0,25	-0,145
ENSOCUG00000029303	RAP1A	-0,25	-0,244
ENSOCUG00000029421	SNX6	-0,25	-0,357
ENSOCUG00000004900	PALLD	-0,26	-0,297
ENSOCUG00000016209	CALCOCO2	-0,26	-0,145
ENSOCUG00000013243	SMC5	-0,26	-0,277
ENSOCUG00000027088	PPP2R2A	-0,26	-0,279
ENSOCUG00000005324	MAP1A	-0,26	-0,275
ENSOCUG00000006625	ACADL	-0,26	-0,705
ENSOCUG00000005116	PIK3R1	-0,26	-0,293
ENSOCUG00000010453	MLIP	-0,26	-0,707
ENSOCUG00000015866	BPTF	-0,26	-0,233
ENSOCUG00000002533	KCMF1	-0,26	-0,404
ENSOCUG00000002827	IDE	-0,26	-0,461
ENSOCUG00000011168	TMEM87A	-0,26	-0,193

ENSOCUG00000006039	KAT2B	-0,26	-0,418
ENSOCUG00000010015	RUFY3	-0,26	-0,249
ENSOCUG00000010587	APP	-0,26	-0,365
ENSOCUG00000016503	WAPL	-0,26	-0,243
ENSOCUG00000011034	STAT3	-0,26	-0,264
ENSOCUG00000013563	EHBP1	-0,27	-0,319
ENSOCUG00000016614	NDEL1	-0,27	-0,322
ENSOCUG00000011647	FOXJ3	-0,27	-0,188
ENSOCUG00000025540	NFYC	-0,27	-0,305
ENSOCUG00000005685	TRIP11	-0,27	-0,351
ENSOCUG00000016438	CSDE1	-0,27	-0,368
ENSOCUG00000015650	TBC1D15	-0,27	-0,313
ENSOCUG00000009420	NLN	-0,28	-0,452
ENSOCUG00000002549	WDR1	-0,28	-0,294
ENSOCUG00000004534	MDN1	-0,28	-0,206
ENSOCUG00000010440	MSI2	-0,28	-0,384
ENSOCUG00000007010	DDX1	-0,28	-0,436
ENSOCUG00000013484	EIF4B	-0,28	-0,344
ENSOCUG00000008790	KMT2E	-0,28	-0,261
ENSOCUG00000003373	ATE1	-0,28	-0,356
ENSOCUG00000012226	SRP72	-0,28	-0,250
ENSOCUG00000006227	JAK1	-0,28	-0,265
ENSOCUG00000007752	SIL1	-0,28	-0,392
ENSOCUG00000003962	PHLDB2	-0,28	-0,498
ENSOCUG00000010949	ZCCHC6	-0,28	-0,253
ENSOCUG00000005143	PSMD4	-0,28	-0,415
ENSOCUG00000014895	NUCKS1	-0,29	-0,230
ENSOCUG00000008154	TMF1	-0,29	-0,332
ENSOCUG00000009944	DYNC1I2	-0,29	-0,291
ENSOCUG00000004657	NR2C1	-0,29	-0,517
ENSOCUG00000004434	CHD1	-0,29	-0,324
ENSOCUG00000012152	MCL1	-0,29	-0,236
ENSOCUG00000009841	GNL3	-0,29	-0,394
ENSOCUG00000003980	TMX1	-0,29	-0,473
ENSOCUG00000027423	RPS29	-0,29	-0,282
ENSOCUG00000010324	KDM3A	-0,30	-0,286
ENSOCUG00000002190	MITF	-0,30	-0,701
ENSOCUG00000015520	PHF3	-0,30	-0,333
ENSOCUG00000004325	TNRC6A	-0,30	-0,302
ENSOCUG00000010396	SSB	-0,30	-0,419
ENSOCUG00000006799	COQ6	-0,30	-0,890
ENSOCUG00000014299	TPP2	-0,30	-0,320
ENSOCUG00000001972	TXNDC12	-0,30	-0,328
ENSOCUG00000023433	ITSN2	-0,30	-0,341
ENSOCUG00000014360	VPS45	-0,30	-0,676

ENSOCUG00000009755	JARID2	-0,30	-0,352
ENSOCUG00000004237	NDUFA2	-0,30	-0,619
ENSOCUG00000003617	MTIF2	-0,30	-0,446
ENSOCUG00000024656	SLC8A1	-0,30	-0,568
ENSOCUG00000023878	SYNE1	-0,30	-0,258
ENSOCUG00000013221	EGLN1	-0,30	-0,707
ENSOCUG00000029277	CHMP3	-0,30	-0,384
ENSOCUG00000015651	ATG3	-0,30	-0,265
ENSOCUG00000004316	NRBP1	-0,30	-0,216
ENSOCUG00000012136	TBK1	-0,31	-0,329
ENSOCUG00000001837	SRSF4	-0,31	-0,503
ENSOCUG00000002928	ERC1	-0,31	-0,481
ENSOCUG00000013170	MARCH1	-0,31	-0,326
ENSOCUG00000022307	RAB18	-0,31	-0,317
ENSOCUG00000007139	FAM149B1	-0,31	-0,350
ENSOCUG00000015438	MIER1	-0,31	-0,242
ENSOCUG00000003409	COPS4	-0,31	-0,589
ENSOCUG00000008328	INTS13	-0,31	-0,394
ENSOCUG00000016393	RB1CC1	-0,31	-0,446
ENSOCUG00000003964	TMED4	-0,31	-0,334
ENSOCUG00000013505	CPSF6	-0,31	-0,222
ENSOCUG00000009197	DCAF13	-0,31	-0,247
ENSOCUG00000017370	FRMD4A	-0,31	-0,410
ENSOCUG00000016875	GATB	-0,31	-0,566
ENSOCUG00000015914	PRRC2C	-0,31	-0,349
ENSOCUG00000026707	TRIM23	-0,32	-0,241
ENSOCUG00000015950	ARHGAP12	-0,32	-0,221
ENSOCUG00000002037	PTPN21	-0,32	-0,261
ENSOCUG00000029239	ZNF717	-0,32	-0,373
ENSOCUG00000002756	CHTOP	-0,32	-0,420
ENSOCUG00000012564	EIF3K	-0,32	-0,264
ENSOCUG00000003439	DAG1	-0,32	-0,410
ENSOCUG00000009067	DHX38	-0,32	-0,329
ENSOCUG00000015847	CSPP1	-0,32	-0,309
ENSOCUG00000006979	HDHD2	-0,32	-0,596
ENSOCUG00000025582	LDB1	-0,32	-0,303
ENSOCUG00000016511	PARK7	-0,32	-0,472
ENSOCUG00000004806	PCNT	-0,32	-0,282
ENSOCUG00000013277	TET1	-0,32	-0,287
ENSOCUG00000015425	KPNB1	-0,32	-0,388
ENSOCUG00000008180	MAP4K4	-0,32	-0,269
ENSOCUG00000004000	EIF4ENIF1	-0,32	-0,361
ENSOCUG00000006879	EFTUD2	-0,32	-0,234
ENSOCUG00000013435	SNX2	-0,32	-0,329
ENSOCUG00000006016	SMS	-0,32	-0,413

ENSOCUG00000016382	ZNF106	-0,32	-0,658
ENSOCUG00000006320	SMARCA2	-0,32	-0,407
ENSOCUG00000013237	AASS	-0,32	-0,386
ENSOCUG00000004961	CELF2	-0,32	-0,368
ENSOCUG00000014670	EI24	-0,33	-0,472
ENSOCUG00000015150	BAZ2B	-0,33	-0,191
ENSOCUG00000016125	PTPRM	-0,33	-0,215
ENSOCUG00000015916	PIP4K2B	-0,33	-0,310
ENSOCUG00000011730	RYBP	-0,33	-0,352
ENSOCUG00000002197	ITGB8	-0,33	-0,415
ENSOCUG00000005932	CCDC93	-0,33	-0,266
ENSOCUG00000009309	GALC	-0,33	-0,476
ENSOCUG00000002181	MDC1	-0,33	-0,294
ENSOCUG00000007408	GNL3L	-0,33	-0,252
ENSOCUG00000014786	INO80D	-0,33	-0,318
ENSOCUG00000002160	C6orf136	-0,33	-0,338
ENSOCUG00000011003	DNAJC11	-0,33	-0,487
ENSOCUG00000005508	CCDC6	-0,33	-0,265
ENSOCUG00000009901	WDR60	-0,33	-0,400
ENSOCUG00000004747	ANTXR2	-0,33	-0,344
ENSOCUG00000006074	TLK2	-0,33	-0,308
ENSOCUG00000016534	TMEM126A	-0,34	-0,749
ENSOCUG00000000572	FAM192A	-0,34	-0,306
ENSOCUG00000007656	TEC	-0,34	-0,817
ENSOCUG00000029308	RAB10	-0,34	-0,324
ENSOCUG00000007779	USP16	-0,34	-0,382
ENSOCUG00000000278	RTF1	-0,34	-0,454
ENSOCUG00000001827	TMEM57	-0,34	-0,496
ENSOCUG00000006874	CFAP36	-0,34	-0,315
ENSOCUG00000010580	ELF1	-0,34	-0,355
ENSOCUG00000000466	MIOS	-0,34	-0,448
ENSOCUG00000009041	BOD1L1	-0,34	-0,355
ENSOCUG00000008962	LNPK	-0,34	-0,377
ENSOCUG00000003747	ZNF384	-0,34	-0,342
ENSOCUG00000001357	HNRNPD	-0,34	-0,336
ENSOCUG00000004832	USP47	-0,34	-0,416
ENSOCUG00000025001	RAD50	-0,34	-0,384
ENSOCUG00000005385	ZFP91	-0,34	-0,372
ENSOCUG00000003262	G3BP2	-0,35	-0,396
ENSOCUG00000013244	CCDC66	-0,35	-0,298
ENSOCUG00000008457	NDUFAB1	-0,35	-0,942
ENSOCUG00000016919	CEP83	-0,35	-0,377
ENSOCUG00000001266	TOMM70	-0,35	-0,536
ENSOCUG00000004136	SEPT7	-0,35	-0,419
ENSOCUG00000008074	MAT2B	-0,35	-0,333

ENSOCUG00000010405	METTL5	-0,35	-0,612
ENSOCUG00000010697	TAF5L	-0,35	-0,398
ENSOCUG00000015514	TOR1AIP1	-0,35	-0,315
ENSOCUG00000001052	ARID4A	-0,35	-0,351
ENSOCUG00000008948	CHMP5	-0,35	-0,316
ENSOCUG00000014396	ZNF532	-0,35	-0,192
ENSOCUG00000012972	CRYAB	-0,35	-0,972
ENSOCUG00000017820	PARP1	-0,35	-0,521
ENSOCUG00000013906	CWC25	-0,36	-0,391
ENSOCUG00000010117	SARNP	-0,36	-0,440
ENSOCUG00000000359	CLK2	-0,36	-0,292
ENSOCUG00000013109	PSMB5	-0,36	-0,588
ENSOCUG00000010309	RPL14	-0,36	-0,288
ENSOCUG00000002251	RCSD1	-0,36	-0,425
ENSOCUG00000017849	GCC2	-0,36	-0,255
ENSOCUG00000006079	BAZ2A	-0,36	-0,176
ENSOCUG00000000303	PPP1R3A	-0,36	-0,930
ENSOCUG00000007051	SUDS3	-0,36	-0,346
ENSOCUG00000015462	PRRG1	-0,36	-0,761
ENSOCUG00000008137	MLLT3	-0,36	-0,501
ENSOCUG00000013239	PRPF40A	-0,36	-0,376
ENSOCUG00000009241	RAB11FIP2	-0,36	-0,395
ENSOCUG00000013372	RRM1	-0,36	-0,248
ENSOCUG00000015936	GSAP	-0,36	-0,286
ENSOCUG00000022928	RPL11	-0,36	-0,420
ENSOCUG00000004566	DBI	-0,36	-0,410
ENSOCUG00000007963	C2orf49	-0,36	-0,288
ENSOCUG00000012638	PSMC6	-0,36	-0,353
ENSOCUG00000006589	KIAA1551	-0,36	-0,305
ENSOCUG00000001414	DDX23	-0,37	-0,327
ENSOCUG00000006235	PPP4R2	-0,37	-0,492
ENSOCUG00000014667	DLD	-0,37	-0,687
ENSOCUG00000015494	NIN	-0,37	-0,342
ENSOCUG00000027456	DSTN	-0,37	-0,489
ENSOCUG00000006642	XRCC5	-0,37	-0,293
ENSOCUG00000007640	GNA13	-0,37	-0,418
ENSOCUG00000017344	KAT6B	-0,37	-0,442
ENSOCUG00000013737	RBM25	-0,37	-0,315
ENSOCUG00000017416	FAM136A	-0,37	-0,599
ENSOCUG00000004532	THRB	-0,37	-0,566
ENSOCUG00000009259	AARSD1	-0,37	-0,326
ENSOCUG00000005372	TNIP1	-0,37	-0,218
ENSOCUG00000008342	FGFR1OP2	-0,37	-0,333
ENSOCUG00000012716	SAP18	-0,37	-0,484
ENSOCUG00000012206	DDX46	-0,37	-0,375

ENSOCUG00000000038	CREBRF	-0,37	-0,269
ENSOCUG00000002555	SCP2	-0,37	-0,497
ENSOCUG00000001320	IFT74	-0,37	-0,319
ENSOCUG00000027851	PARG	-0,37	-0,361
ENSOCUG00000016747	PSMF1	-0,37	-0,517
ENSOCUG00000026553	PSMD8	-0,38	-0,361
ENSOCUG00000000586	VTI1B	-0,38	-0,415
ENSOCUG00000013254	IFNGR1	-0,38	-0,390
ENSOCUG00000003035	SLK	-0,38	-0,384
ENSOCUG00000004848	ROCK1	-0,38	-0,526
ENSOCUG00000027940	HSPA4L	-0,38	-0,400
ENSOCUG00000008397	CTSV	-0,38	-0,390
ENSOCUG00000003169	LRRFIP2	-0,38	-0,694
ENSOCUG00000001645	RAB7A	-0,38	-0,479
ENSOCUG00000001165	RANBP2	-0,38	-0,389
ENSOCUG00000014543	CHMP4B	-0,38	-0,524
ENSOCUG00000001758	PTPN2	-0,39	-0,547
ENSOCUG00000015244	PHYH	-0,39	-0,625
ENSOCUG00000000618	PIAS1	-0,39	-0,286
ENSOCUG00000017493	KIF5B	-0,39	-0,633
ENSOCUG00000006541	ZFP62	-0,39	-0,338
ENSOCUG00000017823	M6PR	-0,39	-0,287
ENSOCUG00000016737	ZFR	-0,39	-0,383
ENSOCUG00000004462	PCF11	-0,39	-0,334
ENSOCUG00000006169	TCERG1	-0,39	-0,414
ENSOCUG00000006605	ARID4B	-0,39	-0,337
ENSOCUG00000023965	SSBP1	-0,39	-0,598
ENSOCUG00000015612	NOL11	-0,39	-0,548
ENSOCUG00000000429	MPHOSPH8	-0,39	-0,412
ENSOCUG00000008592	AHCY	-0,39	-0,356
ENSOCUG00000014607	YWHAE	-0,39	-0,454
ENSOCUG00000004338	TJP1	-0,40	-0,475
ENSOCUG00000011248	C6orf203	-0,40	-0,664
ENSOCUG00000001776	REST	-0,40	-0,421
ENSOCUG00000017050	NFS1	-0,40	-0,574
ENSOCUG00000003009	RBM28	-0,40	-0,464
ENSOCUG00000015547	LIMCH1	-0,40	-0,319
ENSOCUG00000009924	CCDC25	-0,40	-0,376
ENSOCUG00000009613	NKAP	-0,40	-0,443
ENSOCUG00000004419	NDUFC1	-0,40	-0,777
ENSOCUG00000005409	GOLIM4	-0,40	-0,490
ENSOCUG00000014580	ZNF330	-0,40	-0,529
ENSOCUG00000011084	PPIG	-0,40	-0,378
ENSOCUG00000024139	TRA2A	-0,40	-0,314
ENSOCUG00000002954	CASC3	-0,40	-0,395

ENSOCUG00000010593	UQCC1	-0,40	-0,678
ENSOCUG00000002353	TRNAU1AP	-0,40	-0,553
ENSOCUG00000014469	HNRNPU	-0,40	-0,364
ENSOCUG00000002132	PPP1R10	-0,41	-0,375
ENSOCUG00000023943	UBAP2L	-0,41	-0,378
ENSOCUG00000017519	MYO10	-0,41	-0,576
ENSOCUG00000003025	NARS	-0,41	-0,520
ENSOCUG00000012291	ZNF326	-0,41	-0,397
ENSOCUG00000029509	C16orf52	-0,41	-0,522
ENSOCUG00000000095	RBBP5	-0,41	-0,371
ENSOCUG00000009934	DYRK2	-0,41	-0,372
ENSOCUG00000003793	ANAPC13	-0,41	-0,748
ENSOCUG00000000023	PKP2	-0,41	-0,715
ENSOCUG00000010114	BLOC1S1	-0,41	-0,447
ENSOCUG00000004082	TUBA1B	-0,41	-0,401
ENSOCUG00000008901	NAGK	-0,41	-0,443
ENSOCUG00000002031	AGA	-0,41	-0,298
ENSOCUG00000000367	DROSHA	-0,41	-0,271
ENSOCUG00000016958	PCMTD1	-0,41	-0,439
ENSOCUG00000009556	SRFBP1	-0,41	-0,493
ENSOCUG00000009231	OXCT1	-0,41	-0,923
ENSOCUG00000005226	ZNF143	-0,42	-0,464
ENSOCUG00000015484	CRNKL1	-0,42	-0,462
ENSOCUG00000004463	SKIV2L2	-0,42	-0,312
ENSOCUG00000014319	PIK3R3	-0,42	-0,656
ENSOCUG00000002913	BCORL1	-0,42	-0,241
ENSOCUG00000012289	MTAP	-0,42	-0,552
ENSOCUG00000017307	SMC6	-0,42	-0,381
ENSOCUG00000001513	VPS26A	-0,42	-0,439
ENSOCUG00000011427	MAPKAPK5	-0,42	-0,422
ENSOCUG00000002053	DNAJB11	-0,42	-0,374
ENSOCUG00000002551	CNTLN	-0,42	-0,598
ENSOCUG00000025605	CYCS	-0,42	-0,872
ENSOCUG00000005294	RRAGD	-0,42	-0,781
ENSOCUG00000008893	EMC7	-0,42	-0,404
ENSOCUG00000005695	PIGK	-0,42	-0,508
ENSOCUG00000027528	PHACTR4	-0,42	-0,483
ENSOCUG00000006124	LNP1	-0,42	-1,227
ENSOCUG00000011923	RSF1	-0,43	-0,406
ENSOCUG00000007802	CAST	-0,43	-0,525
ENSOCUG00000015417	CSTF3	-0,43	-0,417
ENSOCUG00000010197	PHAX	-0,43	-0,525
ENSOCUG00000003399	HMGN3	-0,43	-0,508
ENSOCUG00000002805	AMPD3	-0,43	-0,520
ENSOCUG00000000249	SMARCD3	-0,43	-0,349

ENSOCUG00000010172	RALBP1	-0,43	-0,325
ENSOCUG00000025756	CXXC1	-0,43	-0,353
ENSOCUG00000014983	PPFIBP2	-0,43	-0,455
ENSOCUG00000026649	SMN1	-0,43	-0,509
ENSOCUG00000013924	OTUD7B	-0,43	-0,572
ENSOCUG00000008720	SEC62	-0,43	-0,316
ENSOCUG00000021080	MBD2	-0,43	-0,312
ENSOCUG00000007692	JPH1	-0,43	-0,729
ENSOCUG00000004524	AKAP9	-0,43	-0,443
ENSOCUG00000025617	CDH13	-0,43	-0,716
ENSOCUG00000004613	LRRC59	-0,43	-0,515
ENSOCUG00000013054	UBE2H	-0,43	-0,474
ENSOCUG00000024862	C6orf106	-0,43	-0,667
ENSOCUG00000006171	MINDY2	-0,43	-0,404
ENSOCUG00000017871	ACTN2	-0,44	-1,099
ENSOCUG00000005420	BMI1	-0,44	-0,440
ENSOCUG00000029298	CIR1	-0,44	-0,407
ENSOCUG00000017804	ZNF131	-0,44	-0,321
ENSOCUG00000009618	SLAIN2	-0,44	-0,473
ENSOCUG00000007468	CCPG1	-0,44	-0,464
ENSOCUG00000000562	PABPC4	-0,44	-0,501
ENSOCUG00000014796	HERC1	-0,44	-0,387
ENSOCUG00000008444	UBFD1	-0,44	-0,533
ENSOCUG00000017662	TERF2	-0,44	-0,360
ENSOCUG00000008215	CHD4	-0,44	-0,478
ENSOCUG00000002214	GLOD4	-0,44	-0,445
ENSOCUG00000016112	METTL16	-0,45	-0,381
ENSOCUG00000012019	RNF20	-0,45	-0,329
ENSOCUG00000006110	EIF4G1	-0,45	-0,518
ENSOCUG00000001349	RBFOX2	-0,45	-0,399
ENSOCUG00000000776	LEMD3	-0,45	-0,400
ENSOCUG00000000408	PDK1	-0,45	-0,813
ENSOCUG00000000848	SUPT6H	-0,45	-0,457
ENSOCUG00000016332	CDC73	-0,45	-0,407
ENSOCUG00000023518	FXR1	-0,45	-0,706
ENSOCUG00000008361	HSPA4	-0,45	-0,555
ENSOCUG00000003818	TRIM44	-0,45	-0,404
ENSOCUG00000016697	MRPS10	-0,45	-0,495
ENSOCUG00000001949	MRPL15	-0,45	-0,671
ENSOCUG00000017534	FNBP4	-0,45	-0,388
ENSOCUG00000024211	JRKL	-0,46	-0,375
ENSOCUG00000004930	RC3H1	-0,46	-0,443
ENSOCUG00000016822	PDCD4	-0,46	-0,412
ENSOCUG00000008913	RPS6KA5	-0,46	-0,603
ENSOCUG00000002965	ZNF652	-0,46	-0,325

ENSOCUG00000009081	SS18	-0,46	-0,391
ENSOCUG00000003420	SAMD4A	-0,46	-0,378
ENSOCUG00000003327	SLTM	-0,46	-0,439
ENSOCUG00000000668	KAT7	-0,46	-0,430
ENSOCUG00000017375	SMARCC2	-0,46	-0,321
ENSOCUG00000015521	CACUL1	-0,46	-0,430
ENSOCUG00000001963	TNS1	-0,46	-0,422
ENSOCUG00000011375	WDR33	-0,46	-0,424
ENSOCUG00000011175	ZNF445	-0,47	-0,460
ENSOCUG00000001119	MRPS22	-0,47	-0,701
ENSOCUG00000010650	KTN1	-0,47	-0,516
ENSOCUG00000015680	PPHLN1	-0,47	-0,433
ENSOCUG00000001407	MAGI2	-0,47	-0,398
ENSOCUG00000001002	ZCCHC7	-0,47	-0,484
ENSOCUG00000008247	TPD52	-0,47	-0,440
ENSOCUG00000004712	GABPB2	-0,47	-0,540
ENSOCUG00000010866	HSD17B4	-0,47	-0,461
ENSOCUG00000002316	RPAP3	-0,47	-0,637
ENSOCUG00000017124	ASB15	-0,47	-0,954
ENSOCUG00000012441	PCGF6	-0,47	-0,742
ENSOCUG00000001751	RND3	-0,47	-0,483
ENSOCUG00000002099	MADD	-0,47	-0,611
ENSOCUG00000023231	MRPS16	-0,47	-0,729
ENSOCUG00000015797	TSPYL1	-0,48	-0,679
ENSOCUG00000012593	BAZ1A	-0,48	-0,630
ENSOCUG00000012366	SLC25A46	-0,48	-0,652
ENSOCUG00000017230	PCCA	-0,48	-0,776
ENSOCUG00000009645	SAT1	-0,48	-0,550
ENSOCUG00000007031	GRHPR	-0,48	-0,484
ENSOCUG00000000582	ARL2BP	-0,48	-0,360
ENSOCUG00000016784	BCAS2	-0,48	-0,706
ENSOCUG00000005425	SNAPIN	-0,48	-0,679
ENSOCUG00000002577	LRIF1	-0,48	-0,490
ENSOCUG00000008045	NCBP3	-0,48	-0,566
ENSOCUG00000000386	RCAN3	-0,48	-0,432
ENSOCUG00000005026	CD2AP	-0,48	-0,433
ENSOCUG00000014256	TSR1	-0,48	-0,547
ENSOCUG00000008316	STRIP2	-0,48	-0,754
ENSOCUG00000029448	UBE2D2	-0,48	-0,660
ENSOCUG00000009681	GSTMYb-3	-0,48	-0,376
ENSOCUG00000005263	TOX	-0,48	-0,838
ENSOCUG00000013643	PDLIM1	-0,48	-0,343
ENSOCUG00000012092	PSMA2	-0,48	-0,723
ENSOCUG00000004117	QKI	-0,48	-0,607
ENSOCUG00000011283	NUDT4	-0,48	-0,528

ENSOCUG00000012324	ALPK2	-0,49	-0,977
ENSOCUG00000002296	GCOM1	-0,49	-0,794
ENSOCUG00000026893	C1orf115	-0,49	-0,769
ENSOCUG00000017403	TATDN1	-0,49	-0,398
ENSOCUG00000015767	OTUD5	-0,49	-0,304
ENSOCUG00000001078	AMOTL1	-0,49	-0,253
ENSOCUG00000011418	STRN3	-0,49	-0,557
ENSOCUG00000000186	RBM27	-0,49	-0,523
ENSOCUG00000002782	LMAN2	-0,49	-0,483
ENSOCUG00000012745	NSFL1C	-0,49	-0,617
ENSOCUG00000014425	FILIP1L	-0,49	-0,515
ENSOCUG00000015730	TAOK1	-0,49	-0,530
ENSOCUG00000001217	MARCH7	-0,49	-0,433
ENSOCUG00000003124	ARHGAP10	-0,49	-0,496
ENSOCUG00000005657	C1orf131	-0,50	-0,518
ENSOCUG00000000852	CCDC181	-0,50	-0,742
ENSOCUG00000015563	TERF2IP	-0,50	-0,420
ENSOCUG00000023887	CD55	-0,50	-0,665
ENSOCUG00000014232	CLINT1	-0,50	-0,518
ENSOCUG00000014998	IL36A	-0,50	-0,459
ENSOCUG00000011743	SLU7	-0,50	-0,370
ENSOCUG00000004244	KLF12	-0,50	-0,389
ENSOCUG00000015489	ARPP19	-0,50	-0,659
ENSOCUG00000011526	AADAT	-0,50	-0,553
ENSOCUG00000000601	SYNCRIP	-0,50	-0,605
ENSOCUG00000001636	UTP18	-0,50	-0,544
ENSOCUG00000016912	RMDN3	-0,50	-0,805
ENSOCUG00000000290	PCDH7	-0,50	-0,721
ENSOCUG00000002217	ARL1	-0,50	-0,756
ENSOCUG00000011629	ANKRD17	-0,50	-0,542
ENSOCUG00000022457	MSANTD4	-0,50	-0,528
ENSOCUG00000010042	MAGI1	-0,50	-0,455
ENSOCUG00000004726	EBAG9	-0,50	-0,760
ENSOCUG00000000897	PRKAA1	-0,51	-0,405
ENSOCUG00000011120	PURA	-0,51	-0,531
ENSOCUG00000004111	MRPL30	-0,51	-0,537
ENSOCUG00000007090	YWHAH	-0,51	-0,758
ENSOCUG00000000987	PNISR	-0,51	-0,439
ENSOCUG00000015051	PPP1R3C	-0,51	-0,944
ENSOCUG00000002121	ABCF1	-0,51	-0,594
ENSOCUG00000016969	AMMECR1	-0,51	-0,455
ENSOCUG00000016820	RIOK3	-0,51	-0,567
ENSOCUG00000006556	OGFRL1	-0,51	-0,471
ENSOCUG00000029397	ANP32B	-0,51	-0,602
ENSOCUG00000012048	OTULIN	-0,51	-0,567

ENSOCUG00000006249	ZNF280B	-0,52	-0,539
ENSOCUG00000013662	ACADM	-0,52	-0,902
ENSOCUG00000011396	XIRP1	-0,52	-0,867
ENSOCUG00000013330	LARP1B	-0,52	-0,692
ENSOCUG00000011993	RNF214	-0,52	-0,562
ENSOCUG00000011792	RPS27	-0,52	-0,559
ENSOCUG00000009038	FRG1	-0,52	-0,425
ENSOCUG00000007073	LARP7	-0,52	-0,581
ENSOCUG00000005927	ACSM5	-0,52	-0,494
ENSOCUG00000022267	DDAH2	-0,52	-0,354
ENSOCUG00000012261	TPR	-0,52	-0,595
ENSOCUG00000017354	C11orf57	-0,52	-0,402
ENSOCUG00000003969	ABHD10	-0,52	-0,826
ENSOCUG00000016225	ATP5G1	-0,52	-0,956
ENSOCUG00000008735	MAK16	-0,52	-0,645
ENSOCUG00000015631	NPHP1	-0,52	-0,485
ENSOCUG00000004653	METAP2	-0,52	-0,636
ENSOCUG00000013317	HDAC8	-0,52	-0,535
ENSOCUG00000028047	YBX1	-0,52	-1,33
ENSOCUG00000003291	GPATCH8	-0,52	-0,498
ENSOCUG00000015886	YTHDC1	-0,52	-0,534
ENSOCUG00000004301	CCDC47	-0,52	-0,597
ENSOCUG00000017817	AHSA2	-0,52	-0,525
ENSOCUG00000000821	TACC2	-0,52	-0,471
ENSOCUG00000016235	CYB5A	-0,52	-0,555
ENSOCUG00000001875	TSPAN12	-0,53	-0,734
ENSOCUG00000005522	TRIM55	-0,53	-1,290
ENSOCUG00000004778	RABEP1	-0,53	-0,714
ENSOCUG00000000906	ANGPTL3	-0,53	-0,633
ENSOCUG00000007585	ARGLU1	-0,53	-0,581
ENSOCUG00000013071	TMCC1	-0,53	-0,453
ENSOCUG00000002581	RAD21	-0,53	-0,739
ENSOCUG00000005046	PRPF4	-0,53	-0,512
ENSOCUG00000020899	ZBTB47	-0,53	-0,399
ENSOCUG00000011142	SMC3	-0,53	-0,476
ENSOCUG00000023806	CHCHD4	-0,53	-0,502
ENSOCUG00000003754	RUNX1T1	-0,53	-0,612
ENSOCUG00000010012	AK4	-0,53	-0,968
ENSOCUG00000002425	RYR-2	-0,53	-1,205
ENSOCUG00000000270	CAPZA1	-0,53	-0,420
ENSOCUG00000013930	NOC3L	-0,53	-0,426
ENSOCUG00000004816	RAB23	-0,53	-0,474
ENSOCUG00000025231	NASP	-0,53	-0,484
ENSOCUG00000009950	ADK	-0,53	-0,850
ENSOCUG00000022967	SNRNP27	-0,53	-0,758

ENSOCUG00000008078	SPTLC1	-0,53	-0,426
ENSOCUG00000028046	C18orf21	-0,53	-0,682
ENSOCUG00000013664	ZCRB1	-0,53	-0,604
ENSOCUG00000017640	Vps4a	-0,54	-0,560
ENSOCUG00000010003	UTP3	-0,54	-0,847
ENSOCUG00000022926	DNAJC1	-0,54	-0,710
ENSOCUG00000014000	TAF7	-0,54	-0,465
ENSOCUG00000013059	BHLHB9	-0,54	-0,539
ENSOCUG00000013066	LARP1	-0,54	-0,501
ENSOCUG00000008933	ATP5G2	-0,54	-0,758
ENSOCUG00000008083	YLPM1	-0,54	-0,497
ENSOCUG00000011903	ZC3H15	-0,54	-0,532
ENSOCUG00000002746	TWNK	-0,54	-0,446
ENSOCUG00000029160	PUS7	-0,54	-0,682
ENSOCUG00000000707	FAM161A	-0,54	-0,684
ENSOCUG00000009319	PSIP1	-0,54	-0,499
ENSOCUG00000013043	MYEF2	-0,55	-0,476
ENSOCUG00000009724	FAIM	-0,55	-0,543
ENSOCUG00000015173	ZC3H13	-0,55	-0,583
ENSOCUG00000006316	PLEKHO1	-0,55	-0,748
ENSOCUG00000014890	PAH	-0,55	-0,830
ENSOCUG00000007968	ZBTB18	-0,55	-0,575
ENSOCUG00000002264	ADCY10	-0,55	-0,498
ENSOCUG00000002949	CISD1	-0,55	-0,839
ENSOCUG00000015635	PSMA6	-0,55	-0,769
ENSOCUG00000012005	ME3	-0,55	-1,140
ENSOCUG00000014267	RDX	-0,55	-0,396
ENSOCUG00000012518	DSC2	-0,55	-0,862
ENSOCUG00000000958	NDUFC2	-0,56	-0,948
ENSOCUG00000016189	MRPL13	-0,56	-0,491
ENSOCUG00000002888	DDX21	-0,56	-0,561
ENSOCUG00000006153	PSPC1	-0,56	-0,499
ENSOCUG00000029655	RNF8	-0,56	-0,679
ENSOCUG00000009083	SSR1	-0,56	-0,572
ENSOCUG00000004312	NCL	-0,56	-0,615
ENSOCUG00000015515	EML5	-0,56	-0,722
ENSOCUG00000002573	UBAP2	-0,56	-0,699
ENSOCUG00000012679	DNAJB4	-0,56	-0,960
ENSOCUG00000001390	GOLGA4	-0,56	-0,762
ENSOCUG00000029674	RPL37	-0,56	-0,652
ENSOCUG00000006571	OTUD3	-0,56	-0,508
ENSOCUG00000006340	MRPL50	-0,56	-0,845
ENSOCUG00000016230	RABGGTB	-0,57	-0,649
ENSOCUG00000015141	ADCY5	-0,57	-0,823
ENSOCUG00000007272	CCDC186	-0,57	-0,517

ENSOCUG00000006471	BEND5	-0,57	-0,609
ENSOCUG00000004255	ZMAT2	-0,57	-0,490
ENSOCUG00000006570	ABRA	-0,57	-1,391
ENSOCUG00000021320	RRBP1	-0,57	-0,480
ENSOCUG00000011788	JTB	-0,57	-0,659
ENSOCUG00000007842	KYAT3	-0,57	-0,759
ENSOCUG00000000963	PRPF38A	-0,57	-0,535
ENSOCUG00000015526	EML4	-0,57	-0,478
ENSOCUG00000015379	SMYD2	-0,57	-0,816
ENSOCUG00000014748	PDHA1	-0,57	-1,013
ENSOCUG00000016114	AFAP1L1	-0,57	-0,608
ENSOCUG00000014034	C3orf38	-0,57	-0,712
ENSOCUG00000007550	MIS18BP1	-0,57	-0,585
ENSOCUG00000016259	DDIT4L	-0,58	-0,937
ENSOCUG00000025387	PALM2-AKAP2	-0,58	-0,258
ENSOCUG00000003796	CEP63	-0,58	-0,528
ENSOCUG00000000733	CBWD1	-0,58	-0,578
ENSOCUG00000007463	CCDC149	-0,58	-0,778
ENSOCUG00000009432	PFDN1	-0,58	-0,951
ENSOCUG00000014391	ACBD3	-0,58	-0,571
ENSOCUG00000010807	SUPT16H	-0,58	-0,541
ENSOCUG00000012181	LEO1	-0,58	-0,533
ENSOCUG00000005414	ESF1	-0,58	-0,642
ENSOCUG00000016811	RBM20	-0,58	-0,784
ENSOCUG00000010791	SRP9	-0,58	-0,847
ENSOCUG00000007174	SATB1	-0,58	-0,797
ENSOCUG00000023315	CTTNBP2NL	-0,58	-0,567
ENSOCUG00000008621	BRPF3	-0,58	-0,297
ENSOCUG00000021671	TBCA	-0,58	-0,769
ENSOCUG00000010331	FAM92A	-0,58	-0,780
ENSOCUG00000004700	CTAGE5	-0,59	-0,721
ENSOCUG00000008064	TRDN	-0,59	-1,027
ENSOCUG00000017863	RPP15	-0,59	-0,764
ENSOCUG00000017104	PPM1G	-0,59	-0,683
ENSOCUG00000008441	TMCO1	-0,59	-0,571
ENSOCUG00000014252	MAPKAPK2	-0,59	-0,834
ENSOCUG00000015722	SF3B2	-0,59	-0,288
ENSOCUG00000001337	PARM1	-0,59	-0,901
ENSOCUG00000029504	SSRP1	-0,59	-0,482
ENSOCUG00000004319	FTSJ3	-0,59	-0,710
ENSOCUG00000008212	DNTTIP2	-0,59	-0,623
ENSOCUG00000013941	CEP250	-0,60	-0,577
ENSOCUG00000009550	AAK1	-0,60	-0,580
ENSOCUG00000017931	DNAJC8	-0,60	-0,704

ENSOCUG00000015290	MYO18A	-0,60	-0,605
ENSOCUG00000017824	CCDC59	-0,60	-0,595
ENSOCUG00000000681	TXLNG	-0,60	-0,734
ENSOCUG00000014599	ZCCHC17	-0,60	-0,562
ENSOCUG00000013417	NAMPT	-0,60	-0,694
ENSOCUG00000005896	INPP4B	-0,60	-0,916
ENSOCUG00000012570	HTATSF1	-0,60	-0,649
ENSOCUG00000006960	C12orf45	-0,60	-0,705
ENSOCUG00000003656	CDH2	-0,61	-0,977
ENSOCUG00000008750	PSMD7	-0,61	-0,798
ENSOCUG00000006121	APLF	-0,61	-0,404
ENSOCUG00000005044	CDC26	-0,61	-0,595
ENSOCUG00000008336	SCAF4	-0,61	-0,673
ENSOCUG00000017723	CAVIN2	-0,61	-0,420
ENSOCUG00000023568	TTC4	-0,61	-0,744
ENSOCUG00000011982	PHACTR1	-0,61	-0,634
ENSOCUG00000007428	SNX27	-0,61	-0,410
ENSOCUG00000004206	NDUFAF1	-0,61	-1,080
ENSOCUG00000002176	PNN	-0,61	-0,611
ENSOCUG00000007161	CTNNBL1	-0,61	-0,371
ENSOCUG00000001940	SMARCC1	-0,62	-0,769
ENSOCUG00000012012	PPIB	-0,62	-0,734
ENSOCUG00000025693	RBM8A	-0,62	-0,726
ENSOCUG00000010198	TPM2	-0,62	-0,399
ENSOCUG00000005957	RNF34	-0,62	-0,836
ENSOCUG00000005376	EIF3J	-0,62	-0,920
ENSOCUG00000004598	TNIK	-0,62	-0,560
ENSOCUG00000010208	DNAJC2	-0,62	-0,676
ENSOCUG00000006446	CBR4	-0,62	-0,436
ENSOCUG00000003576	TENM2	-0,62	-1,424
ENSOCUG00000004413	AP1AR	-0,62	-0,670
ENSOCUG00000015403	HTRA2	-0,62	-0,429
ENSOCUG00000015992	BCAR1	-0,62	-0,512
ENSOCUG00000005507	GNA14	-0,62	-0,571
ENSOCUG00000013058	RNF149	-0,62	-0,528
ENSOCUG00000012690	HSPB1	-0,62	-0,533
ENSOCUG00000012427	TMED5	-0,62	-0,619
ENSOCUG00000013307	RAB5C	-0,62	-0,476
ENSOCUG00000014521	C10orf71	-0,62	-0,599
ENSOCUG00000007217	GPATCH11	-0,63	-0,562
ENSOCUG00000001286	HSBP1	-0,63	-0,678
ENSOCUG00000013857	NAP1L1	-0,63	-0,647
ENSOCUG00000004640	ZRANB2	-0,63	-0,539
ENSOCUG00000006681	ATP5J	-0,63	-1,121
ENSOCUG00000006785	PTGR2	-0,63	-0,880

ENSOCUG00000013858	MRPL18	-0,63	-0,982
ENSOCUG00000003475	EIF4EBP2	-0,63	-0,318
ENSOCUG00000005257	DNAJC21	-0,63	-0,798
ENSOCUG00000017094	SPAG6	-0,63	-0,593
ENSOCUG00000024611	HNRNPM	-0,63	-0,604
ENSOCUG00000009268	RUNDC1	-0,64	-0,706
ENSOCUG00000000218	OTUD6B	-0,64	-0,617
ENSOCUG00000017857	BICD1	-0,64	-0,564
ENSOCUG00000015961	PPP2CA	-0,64	-0,782
ENSOCUG00000021720	RPGR	-0,64	-0,494
ENSOCUG00000002952	UBE2D1	-0,64	-0,827
ENSOCUG00000025115	MPHOSPH10	-0,64	-0,595
ENSOCUG00000000179	RPS15A	-0,64	-0,650
ENSOCUG00000029520	UBE2I	-0,64	-0,532
ENSOCUG00000027678	TSR2	-0,64	-1,044
ENSOCUG00000016363	BCLAF1	-0,65	-0,605
ENSOCUG00000003351	FAM3C	-0,65	-0,735
ENSOCUG00000000588	FAM96B	-0,65	-0,729
ENSOCUG00000002299	ENAH	-0,65	-0,351
ENSOCUG00000007299	FAM172A	-0,65	-0,521
ENSOCUG00000025567	STBD1	-0,65	-0,532
ENSOCUG00000014755	RAB11A	-0,65	-0,642
ENSOCUG00000006173	NOP10	-0,65	-0,938
ENSOCUG00000000009	CLTA	-0,65	-0,664
ENSOCUG00000017351	BLOC1S6	-0,65	-0,520
ENSOCUG00000010810	NENF	-0,65	-0,759
ENSOCUG00000023698	ASB12	-0,66	-0,773
ENSOCUG00000014134	HIRIP3	-0,66	-0,808
ENSOCUG00000002086	PRNP	-0,66	-0,595
ENSOCUG00000021154	EIF5B	-0,66	-0,765
ENSOCUG00000014723	ATG14	-0,66	-0,611
ENSOCUG00000000075	PITPNA	-0,66	-0,584
ENSOCUG00000010545	FRA10AC1	-0,66	-0,839
ENSOCUG00000001999	GOLM1	-0,66	-0,550
ENSOCUG00000012854	FILIP1	-0,66	-0,934
ENSOCUG00000001985	PTER	-0,66	-0,715
ENSOCUG00000025398	CWF19L2	-0,66	-0,767
ENSOCUG00000009122	RAI14	-0,67	-0,563
ENSOCUG00000011253	ZC3H8	-0,67	-0,939
ENSOCUG00000003712	TCEAL9	-0,67	-0,450
ENSOCUG00000026668	SNRPC	-0,67	-1,46
ENSOCUG00000006689	CFDP1	-0,67	-0,672
ENSOCUG00000011554	KIAA2026	-0,67	-0,739
ENSOCUG00000013987	FGF1	-0,67	-0,881
ENSOCUG00000003237	TRIM41	-0,67	-0,518

ENSOCUG00000000473	ECHS1	-0,67	-1,040
ENSOCUG00000001466	MRPL24	-0,67	-0,953
ENSOCUG00000013833	PPARGC1B	-0,67	-0,937
ENSOCUG00000006268	SH3KBP1	-0,68	-1,041
ENSOCUG00000012444	FBXO40	-0,68	-1,234
ENSOCUG00000027324	TAF3	-0,68	-0,703
ENSOCUG00000014043	DNAJC19	-0,68	-0,929
ENSOCUG00000023899	YPEL3	-0,68	-0,601
ENSOCUG00000026261	FAM222B	-0,68	-0,596
ENSOCUG00000012142	RBM33	-0,68	-0,563
ENSOCUG00000029499	CWC27	-0,68	-0,649
ENSOCUG00000010574	DAXX	-0,68	-0,518
ENSOCUG00000029753	FUNDC1	-0,68	-0,711
ENSOCUG00000001919	SDE2	-0,68	-0,950
ENSOCUG00000029154	IFIT3	-0,69	-0,848
ENSOCUG00000014439	MAX	-0,69	-0,674
ENSOCUG00000008755	COX6C	-0,69	-0,985
ENSOCUG00000015457	FAM98A	-0,69	-0,907
ENSOCUG00000029248	TUBG1	-0,69	-0,840
ENSOCUG00000005089	NDUFB6	-0,69	-1,099
ENSOCUG00000016439	PBX2	-0,69	-0,470
ENSOCUG00000001500	NSMCE1	-0,69	-0,534
ENSOCUG00000001571	LAP3	-0,69	-0,728
ENSOCUG00000006172	RPL26L1	-0,69	-0,561
ENSOCUG00000002765	LUC7L3	-0,69	-0,700
ENSOCUG00000012476	RPS8	-0,70	-0,687
ENSOCUG00000013848	KIZ	-0,70	-0,676
ENSOCUG00000000461	C21orf59	-0,70	-0,683
ENSOCUG00000002358	ARHGAP30	-0,70	-0,715
ENSOCUG00000013150	DSP	-0,70	-1,092
ENSOCUG00000007144	DNAJC9	-0,70	-0,578
ENSOCUG00000004854	IWS1	-0,70	-0,809
ENSOCUG00000016834	GABARAPL1	-0,70	-0,574
ENSOCUG00000016255	CREBL2	-0,70	-0,824
ENSOCUG00000008571	MYOM1	-0,70	-1,260
ENSOCUG00000022370	PAIP2B	-0,70	-0,850
ENSOCUG00000016774	ECHDC2	-0,70	-0,701
ENSOCUG00000000205	COX7B	-0,70	-1,162
ENSOCUG00000008274	PROX1	-0,70	-0,662
ENSOCUG00000000358	DCTN3	-0,70	-0,830
ENSOCUG00000006209	HFE2	-0,70	-1,440
ENSOCUG00000024384	SRP14	-0,70	-0,863
ENSOCUG00000000585	RRAD	-0,70	-1,184
ENSOCUG00000010363	RPS23	-0,70	-0,624
ENSOCUG00000001108	SPAG7	-0,71	-0,908

ENSOCUG00000027756	Prpf18	-0,71	-0,633
ENSOCUG00000002110	GNL1	-0,71	-0,504
ENSOCUG00000021027	DYNLRB1	-0,71	-0,989
ENSOCUG00000004332	MYRIP	-0,71	-0,772
ENSOCUG00000001096	TAF1D	-0,71	-1,19
ENSOCUG00000008946	SERBP1	-0,71	-0,872
ENSOCUG00000007912	NSMCE3	-0,71	-0,765
ENSOCUG00000027347	RING1	-0,71	-0,535
ENSOCUG00000013865	PLN	-0,71	-1,288
ENSOCUG00000004223	IK	-0,71	-0,691
ENSOCUG00000004026	CEP128	-0,71	-0,533
ENSOCUG00000004003	MAPK1IP1L	-0,72	-0,465
ENSOCUG00000015788	GRIPAP1	-0,72	-0,687
ENSOCUG00000023276	MORF4L1	-0,72	-0,630
ENSOCUG00000004276	MTERF3	-0,72	-1,305
ENSOCUG00000022509	DMD	-0,72	-1,120
ENSOCUG00000017017	CARNMT1	-0,72	-1,030
ENSOCUG00000000155	NSRP1	-0,72	-0,825
ENSOCUG00000029161	CCDC91	-0,72	-0,624
ENSOCUG00000001496	STARD7	-0,73	-0,736
ENSOCUG00000002001	Srsf11	-0,73	-0,57
ENSOCUG00000000795	ZFAND5	-0,73	-0,619
ENSOCUG00000017886	MRPL47	-0,73	-0,966
ENSOCUG00000017538	DNAJA3	-0,73	-0,990
ENSOCUG00000001469	PDCL3	-0,73	-0,745
ENSOCUG00000008160	FAM71F1	-0,74	-0,664
ENSOCUG00000009848	EEF1B2	-0,74	-0,662
ENSOCUG00000008198	IFFO1	-0,74	-0,531
ENSOCUG00000027067	SMIM4	-0,74	-1,357
ENSOCUG00000027715	PACSIN3	-0,74	-0,849
ENSOCUG00000001678	ACIN1	-0,74	-0,693
ENSOCUG00000013050	SYF2	-0,74	-0,617
ENSOCUG00000005544	MRPS18A	-0,74	-0,624
ENSOCUG00000004298	NT5C1A	-0,74	-0,727
ENSOCUG00000008451	CCDC174	-0,74	-0,630
ENSOCUG00000001319	SREK1	-0,74	-0,719
ENSOCUG00000011696	BAG3	-0,74	-0,766
ENSOCUG00000001454	NES	-0,74	-0,620
ENSOCUG00000017413	COA6	-0,74	-0,837
ENSOCUG00000004256	WBP4	-0,75	-0,557
ENSOCUG00000002112	MYBPC3	-0,75	-1,090
ENSOCUG00000010418	SAP30	-0,75	-0,697
ENSOCUG00000011638	RBM6	-0,75	-0,638
ENSOCUG00000004005	POLD4	-0,75	-0,622
ENSOCUG00000023773	TPT1	-0,75	-0,661

ENSOCUG00000024073	DUSP26	-0,75	-0,652
ENSOCUG00000027275	TM4SF1	-0,76	-0,756
ENSOCUG00000014861	MAPRE3	-0,76	-0,495
ENSOCUG00000024677	ACTR3	-0,76	-1,183
ENSOCUG00000005002	RNF207	-0,76	-0,747
ENSOCUG00000003191	SUGCT	-0,76	-1,192
ENSOCUG00000013805	HNRNPDL	-0,76	-0,728
ENSOCUG00000003043	TYW3	-0,76	-0,730
ENSOCUG00000011824	RIOK1	-0,76	-0,710
ENSOCUG00000023014	AAMP	-0,77	-0,838
ENSOCUG00000006655	HSPA9	-0,77	-1,108
ENSOCUG00000003390	PAF1	-0,77	-0,835
ENSOCUG00000011752	PYGO1	-0,77	-0,482
ENSOCUG00000006212	POLR3GL	-0,78	-0,719
ENSOCUG00000009193	RRP9	-0,78	-1,051
ENSOCUG00000005763	FAM177A1	-0,78	-0,668
ENSOCUG00000013592	RWDD1	-0,78	-0,807
ENSOCUG00000002378	PFDN2	-0,78	-1,035
ENSOCUG00000006219	NUDT2	-0,78	-0,693
ENSOCUG00000004615	BUD13	-0,78	-0,412
ENSOCUG00000021270	PARP3	-0,78	-0,641
ENSOCUG00000014434	AATF	-0,78	-0,702
ENSOCUG00000028153	GON7	-0,79	-1,130
ENSOCUG00000011624	FIBIN	-0,79	-0,798
ENSOCUG00000023754	PNKD	-0,79	-0,825
ENSOCUG00000008113	UQCRB	-0,79	-1,256
ENSOCUG00000002743	HNRNPA3	-0,79	-0,898
ENSOCUG00000010559	PFDN6	-0,79	-1,200
ENSOCUG00000005208	MTHFD2	-0,79	-1,094
ENSOCUG00000027218	SLC22A22	-0,79	-0,656
ENSOCUG00000016712	DNER	-0,80	-0,835
ENSOCUG00000006203	DUSP27	-0,80	-0,979
ENSOCUG00000010919	PABPC5	-0,80	-0,790
ENSOCUG00000010523	DPH5	-0,80	-0,501
ENSOCUG00000021102	SNURF	-0,81	-0,866
ENSOCUG00000005711	CETN3	-0,81	-0,689
ENSOCUG00000005790	FAM81A	-0,81	-0,856
ENSOCUG00000006549	CD1D	-0,81	-0,886
ENSOCUG00000006984	MRPS25	-0,81	-0,960
ENSOCUG00000010925	SNRPF	-0,82	-0,783
ENSOCUG00000003547	MRPS5	-0,82	-0,872
ENSOCUG00000011069	NDUFB3	-0,82	-1,434
ENSOCUG00000015017	MRPS9	-0,82	-0,989
ENSOCUG00000001264	CMYA5	-0,83	-1,355
ENSOCUG00000027424	PRPF38B	-0,83	-0,864

ENSOCUG00000022010	C5orf30	-0,83	-1,090
ENSOCUG00000002920	AIFM1	-0,83	-1,206
ENSOCUG00000026246	SEM1	-0,83	-1,152
ENSOCUG00000002904	CHMP4A	-0,84	-0,657
ENSOCUG00000009933	GCSAM	-0,84	-0,621
ENSOCUG00000007933	C6orf47	-0,84	-0,826
ENSOCUG00000023406	PPP1R14A	-0,84	-0,554
ENSOCUG00000015835	MYO1E	-0,84	-0,386
ENSOCUG00000025074	NDUFB9	-0,84	-1,398
ENSOCUG00000002023	TAF11	-0,84	-0,828
ENSOCUG00000014072	ISCA2	-0,84	-1,274
ENSOCUG00000003923	LSY1	-0,84	-0,848
ENSOCUG00000029108	ND4	-0,84	-1,369
ENSOCUG00000026424	HNRNPAB	-0,84	-0,895
ENSOCUG00000029102	ATP6	-0,85	-1,395
ENSOCUG00000001161	HYPK	-0,85	-1,038
ENSOCUG00000017035	TRIM54	-0,85	-1,459
ENSOCUG00000029086	ND1	-0,85	-1,306
ENSOCUG00000029105	ND3	-0,85	-1,124
ENSOCUG00000011785	KLHL38	-0,85	-0,777
ENSOCUG00000002542	SLC22A2	-0,85	-0,856
ENSOCUG00000010664	HDGF	-0,85	-0,774
ENSOCUG00000003981	ANAPC15	-0,86	-0,94
ENSOCUG00000011821	TPM3	-0,86	-0,686
ENSOCUG00000004031	NUB1	-0,86	-0,911
ENSOCUG00000012659	TUFT1	-0,86	-0,916
ENSOCUG00000006323	INO80E	-0,86	-0,562
ENSOCUG00000008001	SLC38A5	-0,87	-0,740
ENSOCUG00000027476	TSPYL2	-0,87	-0,709
ENSOCUG00000007279	F11	-0,87	-0,992
ENSOCUG00000016425	CCDC112	-0,87	-0,883
ENSOCUG00000016222	TXLNB	-0,87	-1,423
ENSOCUG00000006634	FAM107B	-0,87	-0,774
ENSOCUG00000024972	SNORD15	-0,87	-0,852
ENSOCUG00000011848	PSMB6	-0,87	-0,822
ENSOCUG00000002107	CALM3	-0,88	-0,773
ENSOCUG00000021228	DHRS11	-0,88	-1,327
ENSOCUG00000015401	MXI1	-0,88	-0,741
ENSOCUG00000012173	MEF2D	-0,89	-0,740
ENSOCUG00000016630	KIF21A	-0,89	-0,937
ENSOCUG00000003744	FAM104A	-0,89	-0,575
ENSOCUG00000015719	SCO1	-0,89	-1,196
ENSOCUG00000026892	FKBP3	-0,89	-1,471
ENSOCUG00000012618	DDRGK1	-0,89	-0,695
ENSOCUG00000008959	MYOZ2	-0,90	-1,563

ENSOCUG00000011037	MYH7	-0,90	-1,557
ENSOCUG00000024040	PLSCR1	-0,90	-0,787
ENSOCUG00000011023	NRAP	-0,90	-1,263
ENSOCUG00000007447	RSL24D1	-0,90	-0,757
ENSOCUG00000015700	TRIM26	-0,90	-0,630
ENSOCUG00000016049	COA7	-0,90	-1,166
ENSOCUG00000009658	PES1	-0,90	-0,417
ENSOCUG00000017768	PCP4L1	-0,90	-1,609
ENSOCUG00000005736	FAM184A	-0,91	-0,570
ENSOCUG00000014516	CCDC68	-0,91	-0,654
ENSOCUG00000025551	RPS24	-0,91	-1,171
ENSOCUG00000023038	PTMA	-0,91	-0,771
ENSOCUG00000019837	SNORD89	-0,92	-0,894
ENSOCUG00000009187	TMEM243	-0,92	-0,748
ENSOCUG00000000664	FRMD5	-0,92	-1,282
ENSOCUG00000006760	LAS1L	-0,92	-0,826
ENSOCUG00000023102	C15orf52	-0,92	-1
ENSOCUG00000010712	MFAP1	-0,93	-0,942
ENSOCUG00000017323	THAP12	-0,93	-1,319
ENSOCUG00000029062	CDR2	-0,93	-1,175
ENSOCUG00000006932	PAIP2	-0,93	-0,996
ENSOCUG00000025842	C19H17orf97	-0,94	-0,838
ENSOCUG00000026942	RPS10	-0,94	-0,585
ENSOCUG00000023077	HABP4	-0,94	-1,163
ENSOCUG00000029333	RP9	-0,94	-1,178
ENSOCUG00000004236	TMCO6	-0,94	-0,894
ENSOCUG00000012099	ETFRF1	-0,95	-0,827
ENSOCUG00000005830	HIGD1A	-0,96	-1,316
ENSOCUG00000008707	RTL5	-0,96	-0,578
ENSOCUG00000023130	EWSR1	-0,96	-0,73
ENSOCUG00000025781	GPATCH4	-0,96	-1,179
ENSOCUG00000028175	QRSL1	-0,97	-0,963
ENSOCUG00000027653	LSM3	-0,97	-0,729
ENSOCUG00000014678	KLHL34	-0,97	-1,779
ENSOCUG00000021332	SNORD107	-0,98	-1,190
ENSOCUG00000000569	TERB1	-0,99	-0,843
ENSOCUG00000025062	SDHAF4	-0,99	-1,346
ENSOCUG00000000553	TCEAL1	-0,99	-1,308
ENSOCUG00000026859	HNRNPA2B1	-1,00	-0,894
ENSOCUG00000013961	DNAH11	-1,00	-0,987
ENSOCUG00000024272	NEXN	-1,00	-1,371
ENSOCUG00000016826	SPRYD3	-1,00	-0,774
ENSOCUG00000016107	EFNA5	-1,00	-0,993
ENSOCUG00000007414	NAP1L2	-1,00	-0,943
ENSOCUG00000017131	LMOD2	-1,00	-1,771

ENSOCUG00000021209	MT2D	-1,00	-0,835
ENSOCUG00000019604	snoZ17	-1,01	-1,103
ENSOCUG00000005141	CHST11	-1,01	-1,138
ENSOCUG00000028131	BEX3	-1,01	-1,050
ENSOCUG00000004295	ZRSR2	-1,02	-1,011
ENSOCUG00000029090	ND2	-1,02	-1,476
ENSOCUG00000007983	TNNC1	-1,03	-1,818
ENSOCUG00000004443	HNRNPH3	-1,04	-0,986
ENSOCUG00000016740	DPF3	-1,06	-1,333
ENSOCUG00000026776	TMA7	-1,06	-1,298
ENSOCUG00000027112	RPL28	-1,06	-0,930
ENSOCUG00000017585	FAM105A	-1,06	-1,404
ENSOCUG00000000965	LY96	-1,06	-1,448
ENSOCUG00000019313	SNORD8	-1,07	-0,825
ENSOCUG00000003901	GAR1	-1,07	-0,929
ENSOCUG00000011839	SOX4	-1,08	-0,938
ENSOCUG00000029281	CCL9	-1,08	-1,013
ENSOCUG00000029216	C15orf48	-1,08	-1,003
ENSOCUG00000026622	ZC3H14	-1,09	-1,404
ENSOCUG00000015058	SET	-1,09	-1,121
ENSOCUG00000010213	UPF3B	-1,09	-0,892
ENSOCUG00000012934	GNB3	-1,09	-1,106
ENSOCUG00000010733	CFTR	-1,10	-1,667
ENSOCUG00000014758	MAP3K15	-1,11	-2,794
ENSOCUG00000002838	TAF15	-1,11	-1,087
ENSOCUG00000008065	MINOS1	-1,11	-1,408
ENSOCUG00000028039	CHGB	-1,11	-1,266
ENSOCUG00000007985	STC2	-1,12	-0,921
ENSOCUG00000008954	GADD45A	-1,12	-0,876
ENSOCUG00000025669	THRAP3	-1,13	-1,272
ENSOCUG00000013513	LBH	-1,13	-1,478
ENSOCUG00000029099	COX2	-1,14	-1,558
ENSOCUG00000021449	POLR2D	-1,15	-1,115
ENSOCUG00000017964	TBCC	-1,16	-1,057
ENSOCUG00000002093	COQ7	-1,17	-1,401
ENSOCUG00000003910	ZNHIT6	-1,17	-1,033
ENSOCUG00000006418	NUDC	-1,17	-1,351
ENSOCUG00000020085	SNORD94	-1,18	-0,860
ENSOCUG00000009767	DTNBP1	-1,18	-1,221
ENSOCUG00000007576	FGF16	-1,21	-0,989
ENSOCUG00000009100	ANKRD23	-1,22	-1,431
ENSOCUG00000019747	SNORD24	-1,22	-1,352
ENSOCUG00000024367	RPP38	-1,22	-1,678
ENSOCUG00000017932	ATPIF1	-1,23	-1,759
ENSOCUG00000011026	BCL2L2	-1,23	-1,550

ENSOCUG00000008791	SNAPC5	-1,24	-1,226
ENSOCUG00000026126	MB	-1,24	-1,721
ENSOCUG00000000545	CH25H	-1,25	-1,266
ENSOCUG00000017358	TIMM8B	-1,25	-1,524
ENSOCUG00000024935	PDZK1	-1,26	-2,249
ENSOCUG00000027844	SLC24A1	-1,26	-1,383
ENSOCUG00000000032	NUGGC	-1,28	-1,584
ENSOCUG00000023188	Nr1h5	-1,29	-0,918
ENSOCUG00000004647	CSTF2T	-1,34	-1,300
ENSOCUG00000017531	RPL36	-1,35	-1,297
ENSOCUG00000014931	GBP7	-1,35	-1,415
ENSOCUG00000021507	GNPNAT1	-1,35	-1,049
ENSOCUG00000005829	MOGAT1	-1,37	-1,870
ENSOCUG00000029393	L1TD1	-1,38	-0,975
ENSOCUG00000029107	ND4L	-1,39	-1,909
ENSOCUG00000002210	tceal4	-1,43	-1,447
ENSOCUG00000008715	PIN4	-1,43	-1,409
ENSOCUG00000020966	SNORD77	-1,44	-1,453
ENSOCUG00000011454	HRASLS	-1,45	-1,577
ENSOCUG00000021723	SNORD45	-1,45	-1,72
ENSOCUG00000013904	CISH	-1,46	-1,106
ENSOCUG00000019028	SNORD38	-1,48	-1,657
ENSOCUG00000006091	OSR1	-1,51	-1,551
ENSOCUG00000022097	SNORD81	-1,52	-1,314
ENSOCUG00000018667	SNORD118	-1,53	-0,905
ENSOCUG00000000491	TDRD5	-1,54	-1,038
ENSOCUG00000012431	CCDC12	-1,57	-1,378
ENSOCUG00000013786	ACOT13	-1,57	-1,688
ENSOCUG00000019623	SNORD66	-1,58	-1,469
ENSOCUG00000018137	mir107	-1,62	-3,129
ENSOCUG00000019714	SNORD14	-1,62	-1,86
ENSOCUG00000024064	SNORD83	-1,65	-1,586
ENSOCUG00000021661	5S_rRNA	-1,71	-1,656
ENSOCUG00000015658	LINC00116	-1,71	-1,690
ENSOCUG00000006035	Ccdc162	-1,84	-1,539
ENSOCUG00000022805	ANP32E	-1,87	-1,811
ENSOCUG00000020032	SNORD99	-1,99	-1,941
ENSOCUG00000019567	SNORND104	-2,00	-2,292
ENSOCUG00000003240	IRX5	-2,06	-2,192
ENSOCUG00000019264	mir-30c-2	-2,08	-2,629
ENSOCUG00000018179	SNORD58	-2,12	-1,849
ENSOCUG00000015634	TNNT2	-2,17	-2,862
ENSOCUG00000008100	FAM151A	-2,38	-2,493
ENSOCUG00000009056	PDILT	-3,13	-2,450

DEGS only serotonin		
ID	GENE ID	LogFC
ENSOCUG17894	NFKBIE	2,496
ENSOCUG1645	NTF3	1,819
ENSOCUG1869	SNORA21	1,664
ENSOCUG2143	NEFM	1,631
ENSOCUG25132	RPL23A	1,515
ENSOCUG276	TAF1D	1,460
ENSOCUG4291	IGSF6	1,268
ENSOCUG21581	CETN2	1,242
ENSOCUG16448	CCL21	1,239
ENSOCUG4133	HMGB1	1,217
ENSOCUG23836	NEFL	1,167
ENSOCUG22945	ASNS	1,132
ENSOCUG11792	RPS27	1,135
ENSOCUG17531	RPL36al	1,112
ENSOCUG24462	RPL27	1,641
ENSOCUG27291	DENR	1,521
ENSOCUG2549	snoU2_19	1,381
ENSOCUG26152	DBN1	0,945
ENSOCUG1161	HYPK	0,895
ENSOCUG17359	LSM8	0,845
ENSOCUG155	NSRP1	0,816
ENSOCUG9848	EEF1B2	0,893
ENSOCUG1534	DLG2	0,862
ENSOCUG2829	TBCA	0,798
ENSOCUG4614	SF3B4	0,792
ENSOCUG179	RPS15A	0,779
ENSOCUG24611	HNRNPM	0,776
ENSOCUG4236	TMCO6	0,775
ENSOCUG821	CBX1	0,744
ENSOCUG27112	RPL28	0,742
ENSOCUG994	FKBP1A	0,729
ENSOCUG29397	ANP32B	0,722
ENSOCUG1614	ATAD2	0,720
ENSOCUG25918	HIST2H2AA4	0,713
ENSOCUG977	PRELP	0,683
ENSOCUG732	HMGN2	0,678
ENSOCUG1925	SNRPF	0,669
ENSOCUG21825	HMGN1	0,666
ENSOCUG13753	SYTL2	0,665
ENSOCUG7222	TPM3-rs7	0,664

ENSOCUG221	C5orf3	0,663
ENSOCUG2198	RPS23	0,659
ENSOCUG2915	ND3	0,656
ENSOCUG1712	MFAP1	0,640
ENSOCUG16362	ADAMTS1	0,631
ENSOCUG871	FAT4	0,625
ENSOCUG7286	COL4A4	0,599
ENSOCUG368	RPS2	0,593
ENSOCUG15783	CDKN2AIPNL	0,582
ENSOCUG15894	PYURF	0,571
ENSOCUG12472	RPS24	0,569
ENSOCUG924	SEC11C	0,566
ENSOCUG1469	PDCL3	0,563
ENSOCUG12181	LEO1	0,562
ENSOCUG22967	SNRNP27	0,559
ENSOCUG6641	EPB41	0,552
ENSOCUG14425	FILIP1L	0,552
ENSOCUG1799	EPB41L2	0,549
ENSOCUG13664	ZCRB1	0,548
ENSOCUG2971	ZNF81	0,545
ENSOCUG11373	RPS1	0,544
ENSOCUG1197	PHAX	0,543
ENSOCUG8288	ECM2	0,542
ENSOCUG2542	SLC22A2	0,540
ENSOCUG11854	IKBIP	0,530
ENSOCUG16624	MYH1	0,522
ENSOCUG17932	ATPIF1	0,582
ENSOCUG5414	ESF1	0,518
ENSOCUG11743	SLU7	0,490
ENSOCUG23276	MORF4L1	0,486
ENSOCUG1184	PPIG	0,485
ENSOCUG4223	IK	0,483
ENSOCUG2748	S1A13	0,483
ENSOCUG11788	JTB	0,480
ENSOCUG1257	HTATSF1	0,478
ENSOCUG74	HMGB2	0,478
ENSOCUG7447	RSL24D1	0,477
ENSOCUG2765	LUC7L3	0,476
ENSOCUG1117	SARNP	0,474
ENSOCUG22928	RPL11	0,473
ENSOCUG9299	GNPTAB	0,477
ENSOCUG9924	CCDC25	0,478

ENSOCUG25115	MPHOSPH1	0,462
ENSOCUG4312	NCL	0,452
ENSOCUG5711	CETN3	0,452
ENSOCUG1335	HNRNPA1	0,448
ENSOCUG14267	RDX	0,445
ENSOCUG13737	RBM25	0,435
ENSOCUG2176	PNN	0,432
ENSOCUG632	SMARCA2	0,432
ENSOCUG22295	COX17	0,431
ENSOCUG15438	MIER1	0,430
ENSOCUG13857	NAP1L1	0,429
ENSOCUG2551	CNTLN	0,425
ENSOCUG941	BOD1L1	0,425
ENSOCUG23764	CCDC8	0,425
ENSOCUG9295	EEA1	0,422
ENSOCUG9623	LTV1	0,422
ENSOCUG5336	ABI3BP	0,426
ENSOCUG1175	HACD2	0,419
ENSOCUG2986	ND1	0,416
ENSOCUG21154	EIF5B	0,416
ENSOCUG8127	CALD1	0,412
ENSOCUG463	GTF3C3	0,412
ENSOCUG9645	SAT1	0,413
ENSOCUG8367	MLEC	0,498
ENSOCUG166	RPS12	0,486
ENSOCUG5685	TRIP11	0,471
ENSOCUG6874	CFAP36	0,454
ENSOCUG5553	GNAI3	0,429
ENSOCUG2285	ANP32E	0,418
ENSOCUG27424	PRPF38B	0,399
ENSOCUG347	KRTCAP2	0,398
ENSOCUG917	OGN	0,398
ENSOCUG5724	TTC1	0,397
ENSOCUG2132	RRBP1	0,397
ENSOCUG2225	RPL19	0,395
ENSOCUG665	ARID4B	0,395
ENSOCUG29674	RPL37	0,395
ENSOCUG27851	PARG	0,394
ENSOCUG872	SEC62	0,392
ENSOCUG2217	ARL1	0,392
ENSOCUG938	FRG1	0,390
ENSOCUG29233	ASPH	0,389

ENSOCUG1837	SRSF4	0,388
ENSOCUG26829	CCDC9B	0,388
ENSOCUG29299	CHD3	0,387
ENSOCUG14895	NUCKS1	0,382
ENSOCUG853	NOL8	0,387
ENSOCUG11646	ZFP9	0,380
ENSOCUG6689	CFDP1	0,380
ENSOCUG5247	FSTL1	0,379
ENSOCUG549	GOLIM4	0,379
ENSOCUG9319	PSIP1	0,372
ENSOCUG17849	GCC2	0,372
ENSOCUG2788	ZEB2	0,376
ENSOCUG1855	RPL36AL	0,368
ENSOCUG1277	FYTTD1	0,368
ENSOCUG681	TXLNG	0,366
ENSOCUG11923	RSF1	0,366
ENSOCUG13128	NKTR	0,365
ENSOCUG9	CLTA	0,362
ENSOCUG1643	SEC63	0,362
ENSOCUG987	PNISR	0,361
ENSOCUG1319	SREK1	0,360
ENSOCUG14412	ERLEC1	0,358
ENSOCUG12947	SPCS2	0,356
ENSOCUG139	RPL14	0,347
ENSOCUG4142	MYH9	0,346
ENSOCUG4739	RFC1	0,345
ENSOCUG12	ZCCHC7	0,341
ENSOCUG1843	MSL2	0,349
ENSOCUG15665	NTN4	0,349
ENSOCUG4864	CASK	0,338
ENSOCUG22926	DNAJC1	0,337
ENSOCUG97	SATB2	0,336
ENSOCUG26424	HNRNPAB	0,327
ENSOCUG845	NCBP3	0,327
ENSOCUG139	GOLGA4	0,325
ENSOCUG11554	KIAA226	0,324
ENSOCUG3637	CGNL1	0,324
ENSOCUG6153	PSPC1	0,320
ENSOCUG12546	ZNF462	0,320
ENSOCUG24529	SLC7A12	0,319
ENSOCUG1357	HNRNPD	0,319
ENSOCUG3399	HMGN3	0,316

ENSOCUG7579	ATRX	0,316
ENSOCUG12	ARF1	0,316
ENSOCUG2328	UBTF	0,315
ENSOCUG29654	ZNF568	0,313
ENSOCUG27344	CSF2RB	0,311
ENSOCUG1881	ING3	0,318
ENSOCUG132	IFT74	0,353
ENSOCUG182	MAML2	0,337
ENSOCUG15914	PRRC2C	0,346
ENSOCUG7844	SOS1	0,344
ENSOCUG4346	RBBP6	0,299
ENSOCUG11142	SMC3	0,297
ENSOCUG1226	DDX46	0,291
ENSOCUG5362	CASC4	0,286
ENSOCUG17534	FNBP4	0,283
ENSOCUG1737	SMC6	0,283
ENSOCUG27562	PPP3R1	0,289
ENSOCUG13277	TET1	0,275
ENSOCUG429	CEP29	0,276
ENSOCUG145	ZNF638	0,273
ENSOCUG15484	CRNKL1	0,271
ENSOCUG1288	TMEM16B	0,267
ENSOCUG434	DDX42	0,266
ENSOCUG4778	RABEP1	0,265
ENSOCUG1742	CASP8AP2	0,264
ENSOCUG12142	RBM33	0,262
ENSOCUG6227	JAK1	0,261
ENSOCUG4848	ROCK1	0,267
ENSOCUG4653	METAP2	0,259
ENSOCUG21376	PTPRA	0,259
ENSOCUG1385	HNRNPDL	0,256
ENSOCUG75	PITPNA	0,255
ENSOCUG11638	RBM6	0,245
ENSOCUG29392	HNRNPA3	0,244
ENSOCUG11765	CC2D2A	0,245
ENSOCUG1741	ITPR2	0,243
ENSOCUG4386	NFAT5	0,236
ENSOCUG2	SMCHD1	0,233
ENSOCUG1629	CALCOCO2	0,232
ENSOCUG1312	PHC3	0,236
ENSOCUG1552	PHF3	0,230
ENSOCUG15381	QSER1	0,229

ENSOCUG116	CHD9	0,229
ENSOCUG6443	SECISBP2L	0,225
ENSOCUG2451	EIF4G3	0,224
ENSOCUG464	ZRANB2	0,222
ENSOCUG879	KMT2E	0,220
ENSOCUG431	CCDC47	0,216
ENSOCUG848	SUPT6H	0,200
ENSOCUG1474	DST	0,199
ENSOCUG1587	APP	0,183
ENSOCUG4462	PCF11	0,179
ENSOCUG1651	LNPEP	-0,214
ENSOCUG1546	LRPPRC	-0,242
ENSOCUG653	NDRG2	-0,268
ENSOCUG1365	KLHL24	-0,277
ENSOCUG412	ENTPD4	-0,282
ENSOCUG186	ENO1	-0,282
ENSOCUG919	ADGRA3	-0,284
ENSOCUG21231	CCT5	-0,287
ENSOCUG6592	EIF2S3	-0,293
ENSOCUG21839	ATP2C1	-0,298
ENSOCUG1445	TFAM	-0,347
ENSOCUG25429	RAB12	-0,339
ENSOCUG4268	KIAA368	-0,351
ENSOCUG284	Dcaf11	-0,353
ENSOCUG547	PDPR	-0,355
ENSOCUG69	ATP5B	-0,378
ENSOCUG1455	GPI	-0,382
ENSOCUG25719	Nomo1	-0,389
ENSOCUG749	C6orf89	-0,398
ENSOCUG1553	MAP3K2	-0,427
ENSOCUG9289	CHPT1	-0,495
ENSOCUG7781	POLDIP2	-0,419
ENSOCUG17877	DPY3	-0,424
ENSOCUG1688	HSDL2	-0,442
ENSOCUG27435	RPS11	-0,442
ENSOCUG743	AUH	-0,454
ENSOCUG1328	ARHGEF3	-0,468
ENSOCUG1582	ATP2A2	-0,483
ENSOCUG12	HADHB	-0,485
ENSOCUG6119	PKM	-0,490
ENSOCUG9369	VDAC1	-0,529
ENSOCUG13512	NDUFS2	-0,538

ENSOCUG11782	MIPEP	-0,545
ENSOCUG11847	SLC25A3	-0,542
ENSOCUG3891	UQRC2	-0,543
ENSOCUG1314	CAMTA1	-0,573
ENSOCUG13369	ITGA5	-0,585
ENSOCUG8785	IGF2R	-0,594
ENSOCUG11623	PRMT7	-0,625
ENSOCUG12491	OSGEPL1	-0,636
ENSOCUG11689	PDE4D	-0,642
ENSOCUG7487	SLCO1C1	-0,686
ENSOCUG29279	SZRD1	-0,733
ENSOCUG2556	ECH1	-0,737
ENSOCUG1265	RGP1	-0,785
ENSOCUG146	APOO	-0,883
ENSOCUG9644	TUBE1	-0,919
ENSOCUG8325	ALDH2	-1,214
ENSOCUG12213	DYNC1I1	-1,635
ENSOCUG26758	SNORA68	-1,182
ENSOCUG13999	ZNF593	-1,359
ENSOCUG1388	RBPMS2	-1,574
ENSOCUG27222	FRMPD4	-1,859
ENSOCUG29681	NRXN3	-1,911
ENSOCUG11686	SLC38A3	-2,485
**DEGS only pergolide**		
**ID**	**GENE ID**	**LogFC**
ENSOCUG00000017185	CYP3E1	3,552
ENSOCUG00000001864	AQP8	3,380
ENSOCUG00000007172	SCD	3,356
ENSOCUG00000011380	CIDEA	3,188
ENSOCUG00000009086	KLB	3,150
ENSOCUG00000010189	LEP	2,848
ENSOCUG00000019393	SNORA74	2,832
ENSOCUG00000009443	SLC7A10	2,720
ENSOCUG00000016168	ABCD2	2,659
ENSOCUG00000000957	THRSP	2,636
ENSOCUG00000023401	ADH2-2	2,573
ENSOCUG00000022985	CEBPA	2,389
ENSOCUG00000004222	SLC22A3	2,376
ENSOCUG00000017851	AQP7	2,337
ENSOCUG00000004633	PTGER3	2,264
ENSOCUG00000021038	RPL23	2,217

ENSOCUG00000013069	ELOVL6	2,192
ENSOCUG00000013514	MGST1	2,140
ENSOCUG00000013194	PPARG	2,110
ENSOCUG00000024925	MIS12	1,770
ENSOCUG00000026717	Hist1h3b	1,767
ENSOCUG00000009833	SLC24A3	1,762
ENSOCUG00000021306	HIST1H2AK	1,719
ENSOCUG00000012271	TNFSF13B	1,627
ENSOCUG00000007847	GLYCTK	1,608
ENSOCUG00000024115	ASGR2	1,579
ENSOCUG00000026268	ADIPOQ	1,564
ENSOCUG00000011308	MASTL	1,556
ENSOCUG00000025069	HIST1H2AE	1,516
ENSOCUG00000019693	SNORA55	1,508
ENSOCUG00000011007	LRP2	1,499
ENSOCUG00000027274	ABTB1	1,492
ENSOCUG00000023396	CST6	1,477
ENSOCUG00000013802	ALDOC	1,461
ENSOCUG00000010020	DNAJC6	1,451
ENSOCUG00000027626	CD247	1,419
ENSOCUG00000029367	C1QA	1,405
ENSOCUG00000005192	TMEM136	1,323
ENSOCUG00000015728	GPD1	1,305
ENSOCUG00000017233	HIST1H1D	1,296
ENSOCUG00000006245	FKBPL	1,277
ENSOCUG00000008833	AQP4	1,273
ENSOCUG00000024481	CAPN3	1,270
ENSOCUG00000025743	MAL2	1,270
ENSOCUG00000015308	SLCO1B2	1,269
ENSOCUG00000014223	DGAT2	1,251
ENSOCUG00000006916	BLVRA	1,233
ENSOCUG00000017587	LRRC4C	1,223
ENSOCUG00000009662	BCO2	1,210
ENSOCUG00000009044	CIART	1,201
ENSOCUG00000022840	RAD17	1,186
ENSOCUG00000007438	C3AR1	1,183
ENSOCUG00000004730	SYBU	1,177
ENSOCUG00000011612	RGS18	1,156
ENSOCUG00000017716	CPS1	1,155
ENSOCUG00000000972	SORCS1	1,154
ENSOCUG00000024729	HSPB3	1,136
ENSOCUG00000001824	TLR1	1,127

ENSOCUG00000009657	SDR16C5	1,107
ENSOCUG00000000437	DTL	1,091
ENSOCUG00000008690	TMCO4	1,085
ENSOCUG00000002974	KAZALD1	1,076
ENSOCUG00000009952	NDST3	1,074
ENSOCUG00000011674	BMP2	1,047
ENSOCUG00000003842	CDC42SE2	1,038
ENSOCUG00000023599	APOD	1,033
ENSOCUG00000001130	PIGF	1,009
ENSOCUG00000014710	USH2A	1,004
ENSOCUG00000001910	ADGRL3	0,997
ENSOCUG00000005490	AAR2	0,981
ENSOCUG00000009473	PANX1	0,964
ENSOCUG00000005990	FAM198A	0,959
ENSOCUG00000014832	STEAP2	0,946
ENSOCUG00000012810	C11orf54	0,936
ENSOCUG00000002428	PRADC1	0,935
ENSOCUG00000014800	PLP1	0,923
ENSOCUG00000005457	SLC27A3	0,920
ENSOCUG00000021947	SLC1A3	0,918
ENSOCUG00000000426	RAB29	0,917
ENSOCUG00000002932	RAB33A	0,910
ENSOCUG00000014622	AKR7A2	0,904
ENSOCUG00000000578	PDP2	0,897
ENSOCUG00000006810	ELMOD2	0,892
ENSOCUG00000006003	ERP27	0,890
ENSOCUG00000013253	CNP	0,883
ENSOCUG00000015092	C4orf33	0,882
ENSOCUG00000004058	PEX12	0,875
ENSOCUG00000011287	UBE2NL	0,868
ENSOCUG00000001543	FAM13C	0,858
ENSOCUG00000001144	SLC6A6	0,844
ENSOCUG00000021288	TXNDC9	0,840
ENSOCUG00000017423	PCSK6	0,832
ENSOCUG00000015239	RPS27A	0,832
ENSOCUG00000012531	NEU3	0,830
ENSOCUG00000015622	METTL7A	0,825
ENSOCUG00000024437	TMOD3	0,817
ENSOCUG00000017101	PDZD11	0,815
ENSOCUG00000003683	GPR155	0,815
ENSOCUG00000027426	GXYLT1	0,813
ENSOCUG00000011385	GPR22	0,809

ENSOCUG00000004490	GUCY1A2	0,791
ENSOCUG00000004895	ADAMTSL1	0,789
ENSOCUG00000011475	TOR3A	0,784
ENSOCUG00000008061	4930595M18Rik	0,780
ENSOCUG00000004174	PSD4	0,775
ENSOCUG00000008330	CSRP2	0,773
ENSOCUG00000008651	ITM2A	0,762
ENSOCUG00000015076	FXN	0,750
ENSOCUG00000002795	FMO4	0,747
ENSOCUG00000013790	CMC2	0,747
ENSOCUG00000008546	SLC25A16	0,741
ENSOCUG00000006667	MOCS1	0,730
ENSOCUG00000015755	DENND2D	0,728
ENSOCUG00000017729	SCN7A	0,727
ENSOCUG00000002492	CD36	0,719
ENSOCUG00000000388	LYPLAL1	0,717
ENSOCUG00000003350	MRPS23	0,708
ENSOCUG00000011890	MED30	0,703
ENSOCUG00000006357	ADAMTS12	0,698
ENSOCUG00000014373	C4BPA	0,687
ENSOCUG00000016911	TDRD12	0,685
ENSOCUG00000016368	TMEM14C	0,685
ENSOCUG00000009154	SKP2	0,685
ENSOCUG00000024578	C9orf85	0,680
ENSOCUG00000022231	EMP1	0,679
ENSOCUG00000022997	DNAJB6	0,677
ENSOCUG00000017402	ARSG	0,676
ENSOCUG00000005592	LUM	0,672
ENSOCUG00000003144	BPGM	0,667
ENSOCUG00000022022	ATP5J2	0,667
ENSOCUG00000016548	ROR2	0,662
ENSOCUG00000002522	PTGFR	0,661
ENSOCUG00000000526	PRCP	0,657
ENSOCUG00000026691	SLC31A2	0,656
ENSOCUG00000012719	HSPA12B	0,653
ENSOCUG00000000093	SMYD4	0,648
ENSOCUG00000014585	ZDHHC2	0,646
ENSOCUG00000016211	SNTB1	0,639
ENSOCUG00000015948	COA3	0,636
ENSOCUG00000017549	TWSG1	0,628
ENSOCUG00000013390	MRPL16	0,628
ENSOCUG00000015736	PHLPP1	0,627

ENSOCUG00000024555	KCTD12	0,626
ENSOCUG00000011209	SPATA5L1	0,626
ENSOCUG00000002085	SLC25A36	0,623
ENSOCUG00000003689	NID2	0,623
ENSOCUG00000015110	WDR17	0,620
ENSOCUG00000007507	SLC25A26	0,612
ENSOCUG00000010565	TPCN3	0,606
ENSOCUG00000006500	SLC4A4	0,602
ENSOCUG00000005274	PRLR	0,602
ENSOCUG00000012504	ORMDL1	0,601
ENSOCUG00000010524	RBP4	0,595
ENSOCUG00000011995	TBC1D7	0,595
ENSOCUG00000007182	GAS2L3	0,594
ENSOCUG00000006896	RPP30	0,593
ENSOCUG00000005347	PIF1	0,584
ENSOCUG00000004480	CSRP3	0,579
ENSOCUG00000016457	SIKE1	0,573
ENSOCUG00000015315	ALDH4A1	0,571
ENSOCUG00000015732	FAM198B	0,568
ENSOCUG00000013108	HIKESHI	0,566
ENSOCUG00000000356	GBA	0,562
ENSOCUG00000008513	MYO1D	0,560
ENSOCUG00000001622	RFK	0,553
ENSOCUG00000026291	ARRB1	0,552
ENSOCUG00000016663	KIAA0391	0,548
ENSOCUG00000013383	RTN4IP1	0,545
ENSOCUG00000025632	SUCLA2	0,544
ENSOCUG00000015126	FRMD6	0,543
ENSOCUG00000010780	LRRC8B	0,543
ENSOCUG00000027070	AMPD2	0,537
ENSOCUG00000014051	IL17RD	0,537
ENSOCUG00000005895	ATP6V0B	0,528
ENSOCUG00000013690	FAM49B	0,522
ENSOCUG00000009279	ABCA1	0,522
ENSOCUG00000012046	SNX24	0,521
ENSOCUG00000003516	GYG1	0,518
ENSOCUG00000011711	CHIC2	0,515
ENSOCUG00000013534	ENPP2	0,514
ENSOCUG00000024644	H3F3B	0,512
ENSOCUG00000014367	SGK1	0,510
ENSOCUG00000004184	SLC39A8	0,507
ENSOCUG00000016535	RAP1GDS1	0,507

ENSOCUG00000016313	TDG	0,505
ENSOCUG00000011564	RFX5	0,504
ENSOCUG00000007498	TIMM17A	0,500
ENSOCUG00000012016	SEMA3D	0,498
ENSOCUG00000014454	PARP8	0,496
ENSOCUG00000006034	FHL1	0,494
ENSOCUG00000016164	CYP1B1	0,492
ENSOCUG00000013099	SLC35F5	0,492
ENSOCUG00000025029	CSF1	0,491
ENSOCUG00000002260	ACSS2	0,490
ENSOCUG00000014125	PGM5	0,490
ENSOCUG00000015049	DTX4	0,483
ENSOCUG00000021633	LIMS2	0,479
ENSOCUG00000015970	DENND1B	0,477
ENSOCUG00000007631	GPM6A	0,476
ENSOCUG00000005203	PHTF2	0,471
ENSOCUG00000015544	CLMP	0,469
ENSOCUG00000023110	BC026585	0,469
ENSOCUG00000007922	ST8SIA4	0,469
ENSOCUG00000008667	TYMS	0,469
ENSOCUG00000000697	ELP2	0,468
ENSOCUG00000011737	AMN1	0,467
ENSOCUG00000017118	ADIPOR2	0,467
ENSOCUG00000012850	MIEF1	0,467
ENSOCUG00000002674	KCNG3	0,466
ENSOCUG00000009057	PCDH18	0,466
ENSOCUG00000017858	CLDND1	0,465
ENSOCUG00000001994	LRRC40	0,464
ENSOCUG00000005952	BTBD10	0,462
ENSOCUG00000005022	APEX1	0,458
ENSOCUG00000015257	PSMB4	0,457
ENSOCUG00000003383	MGAT4A	0,450
ENSOCUG00000007520	INTS14	0,450
ENSOCUG00000014269	DAD1	0,446
ENSOCUG00000011469	ELOVL5	0,446
ENSOCUG00000014201	WDYHV1	0,441
ENSOCUG00000008471	RANBP6	0,436
ENSOCUG00000021965	TMEM150C	0,435
ENSOCUG00000007422	TBX20	0,431
ENSOCUG00000029440	PCNX4	0,431
ENSOCUG00000006537	POP5	0,430
ENSOCUG00000017062	ROMO1	0,427

ENSOCUG00000002671	IDH1	0,427
ENSOCUG00000011782	MIPEP	0,422
ENSOCUG00000005779	ACTR1A	0,418
ENSOCUG00000006090	ATP5B	0,418
ENSOCUG00000017609	ERMP1	0,417
ENSOCUG00000004511	HSPB8	0,411
ENSOCUG00000016236	COL15A1	0,411
ENSOCUG00000007781	POLDIP2	0,409
ENSOCUG00000000187	CKAP4	0,407
ENSOCUG00000022653	PISD	0,403
ENSOCUG00000008223	BTG1	0,397
ENSOCUG00000004472	PLPP1	0,395
ENSOCUG00000016992	MTMR9	0,394
ENSOCUG00000028052	AMOTL2	0,393
ENSOCUG00000009065	HNMT	0,392
ENSOCUG00000006192	FOCAD	0,392
ENSOCUG00000015348	DAZAP2	0,391
ENSOCUG00000003163	VCL	0,382
ENSOCUG00000013512	NDUFS2	0,382
ENSOCUG00000009383	CFL2	0,370
ENSOCUG00000009263	NAPB	0,370
ENSOCUG00000005809	FKTN	0,369
ENSOCUG00000006969	DCN	0,367
ENSOCUG00000017242	PDGFC	0,364
ENSOCUG00000027511	BCAP29	0,360
ENSOCUG00000014042	SNRPB	0,359
ENSOCUG00000001464	UBA2	0,357
ENSOCUG00000014879	FEZ2	0,356
ENSOCUG00000009586	TTC27	0,355
ENSOCUG00000012053	PPIC	0,353
ENSOCUG00000012549	MEX3C	0,350
ENSOCUG00000017454	NMD3	0,349
ENSOCUG00000001456	EIF4E	0,348
ENSOCUG00000029317	C1orf123	0,343
ENSOCUG00000005284	IGSF3	0,341
ENSOCUG00000008796	FAM46A	0,340
ENSOCUG00000012735	COQ10B	0,334
ENSOCUG00000012900	RORA	0,331
ENSOCUG00000006637	SLC23A2	0,326
ENSOCUG00000002129	CCNG1	0,318
ENSOCUG00000008664	RABGAP1L	0,314
ENSOCUG00000005664	GNPAT	0,313

ENSOCUG00000001081	WWP1	0,310
ENSOCUG00000015714	TCP11L2	0,309
ENSOCUG00000000898	GPHN	0,306
ENSOCUG00000012609	FBXL17	0,304
ENSOCUG00000015579	UAP1	0,304
ENSOCUG00000005474	SGCB	0,303
ENSOCUG00000002094	LANCL2	0,303
ENSOCUG00000000012	HADHB	0,303
ENSOCUG00000001651	LNPEP	0,301
ENSOCUG00000017572	PPP2R5A	0,300
ENSOCUG00000015960	ACO1	0,299
ENSOCUG00000004353	WDFY1	0,298
ENSOCUG00000008140	TTLL7	0,298
ENSOCUG00000008514	TBCK	0,297
ENSOCUG00000015259	ATP6AP2	0,297
ENSOCUG00000004139	SF3B3	0,295
ENSOCUG00000014563	PRKCI	0,294
ENSOCUG00000005951	PRDX1	0,293
ENSOCUG00000014644	PLA2G4A	0,292
ENSOCUG00000001179	ARHGEF26	0,285
ENSOCUG00000007394	ADCY6	0,276
ENSOCUG00000014244	DBT	0,275
ENSOCUG00000014124	SENP5	0,272
ENSOCUG00000001384	RTTN	0,267
ENSOCUG00000011847	SLC25A3	0,267
ENSOCUG00000009878	XIAP	0,265
ENSOCUG00000021896	EIF5	0,264
ENSOCUG00000013843	STAU2	0,264
ENSOCUG00000001595	CRIM1	0,261
ENSOCUG00000009275	PPP1CC	0,260
ENSOCUG00000013642	DOCK11	0,260
ENSOCUG00000005970	COPG1	0,258
ENSOCUG00000010907	CUL5	0,257
ENSOCUG00000017741	HIF1A	0,256
ENSOCUG00000026586	FEM1B	0,255
ENSOCUG00000003468	CDC37L1	0,253
ENSOCUG00000002236	KIAA0355	0,251
ENSOCUG00000023650	CAMK2D	0,247
ENSOCUG00000010349	HIBADH	0,246
ENSOCUG00000012358	ATP11B	0,246
ENSOCUG00000005590	CBLB	0,241
ENSOCUG00000017199	CCT2	0,241

ENSOCUG00000014519	SYNRG	0,239
ENSOCUG00000008496	GHR	0,237
ENSOCUG00000009943	SLC38A2	0,236
ENSOCUG00000002984	DOCK9	0,234
ENSOCUG00000021959	HECTD4	0,229
ENSOCUG00000007785	MCFD2	0,228
ENSOCUG00000006220	CLCN3	0,228
ENSOCUG00000001043	FERMT2	0,224
ENSOCUG00000012926	ANAPC1	0,217
ENSOCUG00000015832	OPA1	0,213
ENSOCUG00000011290	SMAD2	0,211
ENSOCUG00000001328	VPS13A	0,205
ENSOCUG00000006592	EIF2S3	0,200
ENSOCUG00000008568	ANKRD28	0,196
ENSOCUG00000013326	EIF4G2	0,192
ENSOCUG00000010932	GPBP1	0,192
ENSOCUG00000000097	DSTYK	0,190
ENSOCUG00000008549	UBR5	0,186
ENSOCUG00000008544	ADAR	0,183
ENSOCUG00000015046	LRPPRC	0,181
ENSOCUG00000017135	SLC30A9	0,177
ENSOCUG00000002801	VPS13C	0,176
ENSOCUG00000008986	SBF2	0,175
ENSOCUG00000017465	UBE3A	0,174
ENSOCUG00000010536	RALGAPB	0,166
ENSOCUG00000010660	CUL3	0,163
ENSOCUG00000004696	PSME4	0,163
ENSOCUG00000003125	TRPM7	0,154
ENSOCUG00000014986	ASH1L	-0,12
ENSOCUG00000014261	TDRD3	-0,17
ENSOCUG00000003956	MAP4	-0,17
ENSOCUG00000016984	WWC2	-0,17
ENSOCUG00000025154	MIA3	-0,18
ENSOCUG00000006922	JMJD1C	-0,18
ENSOCUG00000011652	RBM5	-0,19
ENSOCUG00000017910	ZFHX3	-0,19
ENSOCUG00000010089	RPL5	-0,19
ENSOCUG00000022799	WASHC2	-0,19
ENSOCUG00000004220	ERP44	-0,20
ENSOCUG00000010714	SENP6	-0,20
ENSOCUG00000005000	TMX3	-0,20
ENSOCUG00000004678	PHF14	-0,21

ENSOCUG00000013018	SMG6	-0,21
ENSOCUG00000006975	NR2C2	-0,22
ENSOCUG00000005930	ZFPM2	-0,23
ENSOCUG00000008794	RPL4	-0,23
ENSOCUG00000000789	EPC1	-0,23
ENSOCUG00000011937	KIF3A	-0,23
ENSOCUG00000013120	PHC3	-0,23
ENSOCUG00000008879	ARID1B	-0,23
ENSOCUG00000007964	TBC1D4	-0,23
ENSOCUG00000017317	CDC42BPA	-0,24
ENSOCUG00000011770	RPRD2	-0,24
ENSOCUG00000008127	CALD1	-0,24
ENSOCUG00000007152	PAXBP1	-0,24
ENSOCUG00000004290	CEP290	-0,24
ENSOCUG00000023028	UBTF	-0,24
ENSOCUG00000002159	PTPN12	-0,24
ENSOCUG00000014482	PLXDC2	-0,24
ENSOCUG00000011761	SEPT8	-0,24
ENSOCUG00000004545	STK3	-0,25
ENSOCUG00000013128	NKTR	-0,25
ENSOCUG00000014284	TMEM131	-0,25
ENSOCUG00000013042	SMARCA5	-0,25
ENSOCUG00000014822	FAF2	-0,25
ENSOCUG00000009229	IRF2	-0,25
ENSOCUG00000010618	ANK2	-0,26
ENSOCUG00000010191	GON4l	-0,26
ENSOCUG00000014557	CEP57	-0,26
ENSOCUG00000024336	SCARNA7	-0,26
ENSOCUG00000000463	GTF3C3	-0,26
ENSOCUG00000003928	SEPT6	-0,27
ENSOCUG00000003336	RPS6	-0,27
ENSOCUG00000001742	CASP8AP2	-0,27
ENSOCUG00000003637	CGNL1	-0,27
ENSOCUG00000004386	NFAT5	-0,27
ENSOCUG00000010886	NUP107	-0,27
ENSOCUG00000007909	BAG6	-0,27
ENSOCUG00000029606	ZNF345	-0,27
ENSOCUG00000012546	ZNF462	-0,29
ENSOCUG00000012886	SMG5	-0,29
ENSOCUG00000011681	GNAI2	-0,29
ENSOCUG00000002220	P2RX4	-0,30
ENSOCUG00000002325	ZNF609	-0,30

ENSOCUG00000011208	PCYOX1	-0,30
ENSOCUG00000005724	TTC1	-0,30
ENSOCUG00000023611	WDR45	-0,31
ENSOCUG00000017108	ASXL2	-0,31
ENSOCUG00000007320	HMGN2	-0,31
ENSOCUG00000005045	SYNE2	-0,32
ENSOCUG00000005057	IGF2BP2	-0,32
ENSOCUG00000006678	JAM2	-0,33
ENSOCUG00000010353	RBM12B	-0,33
ENSOCUG00000016020	GTF2E2	-0,33
ENSOCUG00000002363	CWC22	-0,34
ENSOCUG00000013733	USP53	-0,34
ENSOCUG00000010670	ZNF436	-0,34
ENSOCUG00000010492	ETS1	-0,35
ENSOCUG00000029299	CHD3	-0,35
ENSOCUG00000007723	NCK1	-0,35
ENSOCUG00000009173	RBM34	-0,35
ENSOCUG00000015776	GOLGA5	-0,36
ENSOCUG00000009691	PSMD3	-0,36
ENSOCUG00000015881	SDCCAG8	-0,36
ENSOCUG00000013378	PAK1IP1	-0,36
ENSOCUG00000005242	QDPR	-0,37
ENSOCUG00000002155	SUPT5H	-0,37
ENSOCUG00000010501	SH3BP5	-0,37
ENSOCUG00000007043	TAOK3	-0,38
ENSOCUG00000025492	EID1	-0,38
ENSOCUG00000029141	PCNP	-0,38
ENSOCUG00000014477	ELK4	-0,38
ENSOCUG00000014859	RERE	-0,38
ENSOCUG00000008142	TCF7L2	-0,39
ENSOCUG00000002398	TBC1D8	-0,39
ENSOCUG00000017019	CDH11	-0,39
ENSOCUG00000008067	EEF2K	-0,39
ENSOCUG00000013425	HEBP2	-0,40
ENSOCUG00000011096	MGAT5	-0,40
ENSOCUG00000015875	RFTN1	-0,40
ENSOCUG00000015569	ANKS1A	-0,41
ENSOCUG00000000510	PPRC1	-0,42
ENSOCUG00000008550	ZHX2	-0,42
ENSOCUG00000013619	WDR44	-0,43
ENSOCUG00000017463	GAB2	-0,43
ENSOCUG00000005020	OSGEP	-0,43

ENSOCUG00000010820	MAML2	-0,43
ENSOCUG00000006424	CEP152	-0,43
ENSOCUG00000010937	SOCS2	-0,44
ENSOCUG00000006961	FGD5	-0,44
ENSOCUG00000008454	CEP112	-0,44
ENSOCUG00000008072	STX18	-0,44
ENSOCUG00000029476	Gm4924	-0,44
ENSOCUG00000002105	AFF1	-0,44
ENSOCUG00000020986	RNF25	-0,44
ENSOCUG00000002673	AKAP12	-0,44
ENSOCUG00000002677	ATXN2L	-0,45
ENSOCUG00000000380	WNT5A	-0,45
ENSOCUG00000010022	E2F4	-0,45
ENSOCUG00000011590	RXRB	-0,45
ENSOCUG00000012510	HOMEZ	-0,45
ENSOCUG00000008416	ZNF398	-0,45
ENSOCUG00000007362	PRKCA	-0,46
ENSOCUG00000012045	PTPRB	-0,46
ENSOCUG00000015984	CASC1	-0,46
ENSOCUG00000008718	TMEM231	-0,47
ENSOCUG00000016598	PROCR	-0,47
ENSOCUG00000001489	ADGRF5	-0,48
ENSOCUG00000004565	ABCF3	-0,48
ENSOCUG00000011338	TRIM25	-0,49
ENSOCUG00000008374	UNC119B	-0,49
ENSOCUG00000002631	MSANTD2	-0,50
ENSOCUG00000002468	KIF20B	-0,50
ENSOCUG00000012279	C14orf37	-0,51
ENSOCUG00000008572	RBPMS	-0,51
ENSOCUG00000002310	TNFAIP3	-0,51
ENSOCUG00000026517	RAI2	-0,51
ENSOCUG00000003698	KLF3	-0,52
ENSOCUG00000015077	SCLT1	-0,52
ENSOCUG00000025214	GZF1	-0,52
ENSOCUG00000004762	SYNJ2	-0,52
ENSOCUG00000015469	CTSO	-0,53
ENSOCUG00000003495	MKNK1	-0,54
ENSOCUG00000010633	HMOX1	-0,54
ENSOCUG00000008461	PALB2	-0,54
ENSOCUG00000000633	DLGAP4	-0,56
ENSOCUG00000022117	RREB1	-0,56
ENSOCUG00000000574	PLEKHH1	-0,56

ENSOCUG00000007231	EMCN	-0,56
ENSOCUG00000002395	USP21	-0,56
ENSOCUG00000016464	TYW5	-0,57
ENSOCUG00000008277	FNBP1L	-0,57
ENSOCUG00000010454	CPEB3	-0,58
ENSOCUG00000014141	TRIM39-RPP21	-0,58
ENSOCUG00000026766	SCNM1	-0,59
ENSOCUG00000016557	TXNDC17	-0,60
ENSOCUG00000014601	CD59	-0,60
ENSOCUG00000013467	RNF150	-0,61
ENSOCUG00000014289	MYC	-0,61
ENSOCUG00000014657	EFEMP1	-0,62
ENSOCUG00000012502	GBP4	-0,62
ENSOCUG00000002117	PRR3	-0,63
ENSOCUG00000016078	METTL6	-0,63
ENSOCUG00000029649	HEY2	-0,63
ENSOCUG00000001879	SPAG1	-0,63
ENSOCUG00000014876	ARL5B	-0,64
ENSOCUG00000010972	RLA-A1	-0,64
ENSOCUG00000014919	SNX12	-0,64
ENSOCUG00000010544	CROT	-0,64
ENSOCUG00000001039	SYNGAP1	-0,65
ENSOCUG00000027759	NOS1AP	-0,65
ENSOCUG00000015293	PORCN	-0,65
ENSOCUG00000016938	UBXN11	-0,65
ENSOCUG00000016744	SPOCK2	-0,65
ENSOCUG00000015802	FBXW10	-0,66
ENSOCUG00000017288	CYB5D1	-0,66
ENSOCUG00000024926	NMRAL1	-0,66
ENSOCUG00000017039	RPUSD4	-0,66
ENSOCUG00000008192	SERTAD2	-0,68
ENSOCUG00000013722	PAWR	-0,69
ENSOCUG00000004283	ZNF483	-0,70
ENSOCUG00000007443	RAB11FIP4	-0,71
ENSOCUG00000015220	PECAM1	-0,71
ENSOCUG00000027415	SP2	-0,71
ENSOCUG00000006791	FAM161B	-0,71
ENSOCUG00000000812	CCDC39	-0,72
ENSOCUG00000001306	SPTLC3	-0,72
ENSOCUG00000024218	NFKBIB	-0,73
ENSOCUG00000014753	TMEM218	-0,73
ENSOCUG00000004231	BACH2	-0,73

ENSOCUG00000028355	SCARNA1	-0,74
ENSOCUG00000010935	CLEC4A	-0,75
ENSOCUG00000007489	SHISA4	-0,76
ENSOCUG00000024015	RAD9A	-0,76
ENSOCUG00000012640	CGN	-0,78
ENSOCUG00000001685	SLC7A8	-0,79
ENSOCUG00000015859	PRKCH	-0,81
ENSOCUG00000015295	WDR54	-0,81
ENSOCUG00000004261	SLC35G1	-0,81
ENSOCUG00000013552	KCNN3	-0,83
ENSOCUG00000027066	RGS2	-0,85
ENSOCUG00000008922	FGR	-0,89
ENSOCUG00000029595	AABR07053179.1	-0,89
ENSOCUG00000009553	IQUB	-0,90
ENSOCUG00000001473	S100B	-0,92
ENSOCUG00000002478	TCEAL3	-0,92
ENSOCUG00000025132	RPL23A	-0,93
ENSOCUG00000011541	ILDR1	-0,93
ENSOCUG00000015667	SH3RF2	-0,94
ENSOCUG00000021292	RPL38	-0,94
ENSOCUG00000000779	RSPO2	-0,97
ENSOCUG00000029564	WIPF2	-0,99
ENSOCUG00000017120	PDK4	-1,00
ENSOCUG00000019175	SNORA7	-1,03
ENSOCUG00000000191	YPEL4	-1,03
ENSOCUG00000018491	let-7d	-1,22
ENSOCUG00000026507	Hist1h3g	-1,23
ENSOCUG00000010624	POLR3G	-1,27
ENSOCUG00000001162	SERINC4	-1,28
ENSOCUG00000019702	SNORD59	-1,29
ENSOCUG00000014718	NOS1	-1,30
ENSOCUG00000016145	IL17B	-1,33
ENSOCUG00000024432	IFGGA1	-1,34
ENSOCUG00000017251	GBP1	-1,36
ENSOCUG00000000621	INHBA	-1,37
ENSOCUG00000018323	let-7a-1	-1,37
ENSOCUG00000022434	CDCA3	-1,52
ENSOCUG00000029211	ZSCAN12	-1,56
ENSOCUG00000018518	SNORD49	-1,57
ENSOCUG00000018370	SNORD56	-1,57
ENSOCUG00000022445	MYH2	-1,59
ENSOCUG00000022248	RUFY4	-1,63

ENSOCUG00000006847	CHMP4C	-1,65
ENSOCUG00000019315	SNORD61	-1,71
ENSOCUG00000025588	FAM173B	-1,73
ENSOCUG00000008034	TTC30A	-1,75
ENSOCUG00000020068	SNORD110	-1,86
ENSOCUG00000020076	SNORD75	-1,92
ENSOCUG00000014915	STAC2	-1,94
ENSOCUG00000028024	IRGM	-2,16
ENSOCUG00000003049	COL17A1	-2,24
ENSOCUG00000017082	IGSF11	-2,63
**DEGS only dexfenfluramine**		
**ID**	**GENE ID**	**logFC**
ENSOCUG00000028463	SCARNA20	2,187704
ENSOCUG00000011440	GRID2	2,176072
ENSOCUG00000013625	PRSS12	2,133391
ENSOCUG00000021631	SNORA53	2,043845
ENSOCUG00000001739	CADM2	1,910987
ENSOCUG00000022661	RPSA	1,709552
ENSOCUG00000000724	SLC7A7	1,687704
ENSOCUG00000010041	FAM189B	1,567685
ENSOCUG00000006388	ANKRD13D	1,544701
ENSOCUG00000010077	KCTD19	1,529498
ENSOCUG00000002994	CCDC3	1,511807
ENSOCUG00000012865	GRID1	1,502851
ENSOCUG00000007289	PNMA1	1,437319
ENSOCUG00000000247	CHPF2	1,425365
ENSOCUG00000011294	TDH	1,389964
ENSOCUG00000013333	HS3ST3A1	1,376829
ENSOCUG00000016772	DDIT4	1,360496
ENSOCUG00000011799	FAM221A	1,323697
ENSOCUG00000000355	CNTFR	1,32075
ENSOCUG00000025012	ANGPT4	1,308579
ENSOCUG00000016289	HPGD	1,301636
ENSOCUG00000003500	FAM84B	1,287007
ENSOCUG00000023919	TAGLN	1,286055
ENSOCUG00000005539	NLGN2	1,283886
ENSOCUG00000019359	SNORA61	1,279636
ENSOCUG00000021036	NCKIPSD	1,2154
ENSOCUG00000002250	GGT7	1,213969
ENSOCUG00000009025	PARD6A	1,20978
ENSOCUG00000005593	FXYD6	1,208708
ENSOCUG00000002733	CACNA1G	1,206613

ENSOCUG00000007620	CYP26B1	1,206403
ENSOCUG00000013880	SORCS3	1,19494
ENSOCUG00000017309	HTR4	1,194316
ENSOCUG00000000657	KCNH1	1,194228
ENSOCUG00000024804	BTBD19	1,190608
ENSOCUG00000002851	SH2B3	1,189519
ENSOCUG00000016039	ZNF396	1,173906
ENSOCUG00000011507	RNF185	1,167419
ENSOCUG00000026428	GHDC	1,165074
ENSOCUG00000003688	CPPED1	1,160335
ENSOCUG00000026933	NPTXR	1,150533
ENSOCUG00000010661	PPIL3	1,109929
ENSOCUG00000004487	DRP2	1,098822
ENSOCUG00000011212	TP53I3	1,087849
ENSOCUG00000014979	MBNL3	1,085538
ENSOCUG00000006895	GFAP	1,085483
ENSOCUG00000028344	SCARNA11	1,06768
ENSOCUG00000022769	ZFHX3	1,058024
ENSOCUG00000017484	PNPO	1,046557
ENSOCUG00000026592	RPL13A	1,044945
ENSOCUG00000001302	MICALCL	1,03799
ENSOCUG00000009360	MST1R	1,031201
ENSOCUG00000026354	PGF	1,029031
ENSOCUG00000015745	RPUSD2	1,017141
ENSOCUG00000013688	TNFAIP6	1,012661
ENSOCUG00000002630	HAPLN1	1,00422
ENSOCUG00000001923	CXCR4	1,003341
ENSOCUG00000015723	SLC15A3	0,995412
ENSOCUG00000008824	LRGUK	0,982778
ENSOCUG00000013686	WNT5B	0,971545
ENSOCUG00000025460	RASSF1	0,971083
ENSOCUG00000006326	ALG14	0,95756
ENSOCUG00000026184	VSNL1	0,942036
ENSOCUG00000005204	RGS4	0,940263
ENSOCUG00000010643	KCNMB3	0,937813
ENSOCUG00000016970	DNHD1	0,937166
ENSOCUG00000006393	SSH3	0,932521
ENSOCUG00000003347	SEMA4C	0,932129
ENSOCUG00000010111	P4HA3	0,927842
ENSOCUG00000004385	KCNV2	0,927347
ENSOCUG00000007084	CHRD	0,914794
ENSOCUG00000011348	CHPF	0,895211

ENSOCUG00000017628	CFAP54	0,890077
ENSOCUG00000003523	SYT6	0,886176
ENSOCUG00000016354	IL6R	0,88171
ENSOCUG00000015143	C1QB	0,876799
ENSOCUG00000022879	NME7	0,870249
ENSOCUG00000023498	TBX2	0,85213
ENSOCUG00000010390	TMEM67	0,845033
ENSOCUG00000013309	TRHDE	0,840908
ENSOCUG00000005913	CHCHD7	0,840828
ENSOCUG00000007360	PTS	0,839117
ENSOCUG00000014422	HIST1H1C	0,834149
ENSOCUG00000021324	NDP	0,831953
ENSOCUG00000000638	GLI2	0,826072
ENSOCUG00000007767	SLC11A2	0,816802
ENSOCUG00000012166	FMNL1	0,813633
ENSOCUG00000003633	GFRA1	0,810722
ENSOCUG00000009729	RNF43	0,810089
ENSOCUG00000027266	KCTD21	0,80679
ENSOCUG00000017593	CCDC71	0,804068
ENSOCUG00000007583	TTC39A	0,802692
ENSOCUG00000013700	CTSF	0,793458
ENSOCUG00000029716	ARHGEF17	0,791158
ENSOCUG00000010845	COL4A6	0,789933
ENSOCUG00000002486	COL6A6	0,788636
ENSOCUG00000011031	CDAN1	0,785076
ENSOCUG00000000655	HHAT	0,780063
ENSOCUG00000009517	SLIT2	0,775276
ENSOCUG00000004555	PCDHB4	0,773562
ENSOCUG00000009531	ANGEL1	0,76958
ENSOCUG00000002945	MEOX1	0,764491
ENSOCUG00000001599	FLRT3	0,762414
ENSOCUG00000005498	AGPAT1	0,761506
ENSOCUG00000017214	RIC3	0,761151
ENSOCUG00000007827	SASS6	0,748456
ENSOCUG00000003023	B4GALT7	0,742999
ENSOCUG00000012702	SIGLEC1	0,741874
ENSOCUG00000009266	CAND2	0,740735
ENSOCUG00000003323	ARHGAP28	0,740342
ENSOCUG00000011758	ARHGAP23	0,738018
ENSOCUG00000000227	ASAP3	0,735665
ENSOCUG00000010032	THBS3	0,726285
ENSOCUG00000000128	C21orf91	0,726081

ENSOCUG00000008686	TNK2	0,720308
ENSOCUG00000001278	INTU	0,710513
ENSOCUG00000001086	RMDN1	0,710062
ENSOCUG00000016570	SLCO1A2	0,708425
ENSOCUG00000005524	TMBIM1	0,707062
ENSOCUG00000026758	SNORA68	0,70141
ENSOCUG00000012614	TXNDC16	0,69923
ENSOCUG00000015328	MOGS	0,695901
ENSOCUG00000006557	H2-T24	0,694526
ENSOCUG00000002563	SAT2	0,692537
ENSOCUG00000025261	WRAP53	0,692004
ENSOCUG00000012868	CHST10	0,69164
ENSOCUG00000017934	ACVR1B	0,691018
ENSOCUG00000026610	TMEM100	0,689379
ENSOCUG00000010053	SLC9A5	0,686704
ENSOCUG00000012928	ENPP4	0,686096
ENSOCUG00000015440	BCAR3	0,683642
ENSOCUG00000000312	IGDCC4	0,681519
ENSOCUG00000014499	CLSTN3	0,676194
ENSOCUG00000015928	Wnk4	0,670286
ENSOCUG00000017400	POP1	0,667632
ENSOCUG00000015924	MCM2	0,665836
ENSOCUG00000000960	SDK2	0,662814
ENSOCUG00000004030	GLIS3	0,662539
ENSOCUG00000002341	CAPG	0,660999
ENSOCUG00000008558	PPM1M	0,65884
ENSOCUG00000015807	TFE3	0,656783
ENSOCUG00000008740	TTI2	0,655636
ENSOCUG00000010874	COL4A5	0,655518
ENSOCUG00000002958	ADCY4	0,654483
ENSOCUG00000005579	ITGB3	0,650815
ENSOCUG00000004035	IRF6	0,650495
ENSOCUG00000000927	ADAMTSL4	0,646351
ENSOCUG00000011537	TTC30B	0,646325
ENSOCUG00000007046	PPIL1	0,642667
ENSOCUG00000028187	SLC41A1	0,64086
ENSOCUG00000022299	PI15	0,639318
ENSOCUG00000013427	DPEP2	0,635616
ENSOCUG00000012217	APCDD1	0,633825
ENSOCUG00000026764	CAVIN1	0,633082
ENSOCUG00000017263	RFL	0,632366
ENSOCUG00000009145	IQCB1	0,629998

ENSOCUG00000000611	FKBP10	0,629411
ENSOCUG00000001915	PYCR2	0,627461
ENSOCUG00000010343	TRIM27	0,626218
ENSOCUG00000003031	GGH	0,62585
ENSOCUG00000027852	IQSEC2	0,625749
ENSOCUG00000005759	PHGDH	0,62387
ENSOCUG00000016160	ARHGAP1	0,623387
ENSOCUG00000013452	PRTFDC1	0,622631
ENSOCUG00000011100	RANBP17	0,620277
ENSOCUG00000005766	ITPR3	0,617908
ENSOCUG00000013464	MYSM10	0,617272
ENSOCUG00000008821	YIPF1	0,616523
ENSOCUG00000010123	CD9	0,61284
ENSOCUG00000009342	THAP11	0,60598
ENSOCUG00000029380	ZNF311	0,605541
ENSOCUG00000029449	NUP37	0,605147
ENSOCUG00000003015	IMPDH1	0,604133
ENSOCUG00000016536	PARP11	0,602368
ENSOCUG00000023006	RAB34	0,600104
ENSOCUG00000007851	PLBD2	0,597521
ENSOCUG00000017352	ESYT1	0,59698
ENSOCUG00000001279	TSPAN2	0,595806
ENSOCUG00000008578	DYNLRB2	0,59079
ENSOCUG00000023782	IL11RA	0,590695
ENSOCUG00000009622	PDLIM7	0,590283
ENSOCUG00000023236	TTLL4	0,587257
ENSOCUG00000002350	SMG8	0,585428
ENSOCUG00000026532	SCARA5	0,584636
ENSOCUG00000010095	ZNF18	0,582159
ENSOCUG00000015630	ZNF76	0,579108
ENSOCUG00000013982	ADSS	0,578548
ENSOCUG00000012438	NBEAL2	0,577229
ENSOCUG00000014527	CTSC	0,575122
ENSOCUG00000011883	COG1	0,569802
ENSOCUG00000012374	FRMPD3	0,562932
ENSOCUG00000014077	LTBP2	0,561393
ENSOCUG00000026693	TNS2	0,560735
ENSOCUG00000000826	DENND6A	0,558993
ENSOCUG00000007609	PBX1	0,557903
ENSOCUG00000003679	GSTA1	0,55615
ENSOCUG00000005190	DHCR24	0,555441
ENSOCUG00000012115	AXIN2	0,552092

ENSOCUG00000012232	KIRREL1	0,552028
ENSOCUG00000012661	PTPRU	0,549824
ENSOCUG00000013993	PCDHGC5	0,549132
ENSOCUG00000012063	JUP	0,548108
ENSOCUG00000003594	OLFML2B	0,546848
ENSOCUG00000003174	FARP1	0,546592
ENSOCUG00000013176	SMO	0,544871
ENSOCUG00000005109	MMP19	0,542669
ENSOCUG00000029415	RNF38	0,54117
ENSOCUG00000010372	MAP3K21	0,53866
ENSOCUG00000014489	TMBIM4	0,538212
ENSOCUG00000004708	DCTD	0,537842
ENSOCUG00000000832	ABHD6	0,536907
ENSOCUG00000010087	SPATA13	0,535241
ENSOCUG00000007510	LRIG1	0,535061
ENSOCUG00000002730	LRRC1	0,532145
ENSOCUG00000014984	POLR2C	0,529403
ENSOCUG00000012244	PLIN2	0,526587
ENSOCUG00000015575	GABBR1	0,525154
ENSOCUG00000013581	GGPS1	0,524517
ENSOCUG00000001446	ZDHHC20	0,521623
ENSOCUG00000002267	GSS	0,521384
ENSOCUG00000012916	P3H3	0,520427
ENSOCUG00000011095	SAP30L	0,519317
ENSOCUG00000016385	CADM3	0,518951
ENSOCUG00000026930	RGL2	0,517995
ENSOCUG00000012317	RNF121	0,517465
ENSOCUG00000004406	FBXL7	0,513778
ENSOCUG00000002323	PATZ1	0,511985
ENSOCUG00000011080	GALNT7	0,510451
ENSOCUG00000010218	CMTR2	0,508259
ENSOCUG00000010672	HS2ST1	0,507955
ENSOCUG00000007788	TTC7A	0,507012
ENSOCUG00000000416	ARMCX2	0,502038
ENSOCUG00000015039	FNDC1	0,501947
ENSOCUG00000008730	LRRK1	0,501312
ENSOCUG00000002407	MFAP5	0,50089
ENSOCUG00000017793	CD38	0,500579
ENSOCUG00000012400	PTH1R	0,499459
ENSOCUG00000022838	RBM14	0,498461
ENSOCUG00000017672	TMEM184B	0,49636
ENSOCUG00000014468	C1S	0,495449

ENSOCUG00000011000	GAS7	0,490935
ENSOCUG00000006461	GALK2	0,486389
ENSOCUG00000014193	TRPM3	0,48488
ENSOCUG00000008054	CYP20A1	0,484404
ENSOCUG00000015752	ACSS3	0,48342
ENSOCUG00000012769	TSC22D1	0,483294
ENSOCUG00000017706	ATL1	0,482435
ENSOCUG00000003211	PHYKPL	0,479969
ENSOCUG00000009914	ARHGEF40	0,478647
ENSOCUG00000001754	FMNL3	0,476502
ENSOCUG00000002942	DHRS1	0,4756
ENSOCUG00000010779	FBXO34	0,475271
ENSOCUG00000013015	IFT122	0,47339
ENSOCUG00000011945	TRMT2B	0,472391
ENSOCUG00000009866	ANKRD29	0,471673
ENSOCUG00000011350	SPON1	0,470616
ENSOCUG00000004479	SLC38A9	0,468226
ENSOCUG00000025823	SUPT4H1	0,467436
ENSOCUG00000014539	KDELC2	0,467424
ENSOCUG00000013285	KAT2A	0,467385
ENSOCUG00000004378	ARRDC3	0,467303
ENSOCUG00000015163	NYNRIN	0,467083
ENSOCUG00000011969	MAPKBP1	0,466681
ENSOCUG00000001816	PLCE1	0,463643
ENSOCUG00000024538	C17orf80	0,461145
ENSOCUG00000005271	PIGN	0,458919
ENSOCUG00000000765	HACL1	0,45728
ENSOCUG00000014914	AEBP1	0,457098
ENSOCUG00000012340	TBC1D8B	0,452588
ENSOCUG00000010546	RPS18	0,451895
ENSOCUG00000013363	STIM1	0,451204
ENSOCUG00000024644	HHT3	0,450639
ENSOCUG00000017517	TMEM68	0,450133
ENSOCUG00000004469	ZDHHC13	0,447622
ENSOCUG00000009782	AP5M1	0,447069
ENSOCUG00000006700	MAN1A1	0,446807
ENSOCUG00000008980	BAHD1	0,446297
ENSOCUG00000009709	MED24	0,445637
ENSOCUG00000013453	HMCN1	0,444291
ENSOCUG00000005277	NOD1	0,441568
ENSOCUG00000008284	CAMLG	0,440626
ENSOCUG00000010436	LEF1	0,439436

ENSOCUG00000012547	USP54	0,438764
ENSOCUG00000005975	ACTR1B	0,437728
ENSOCUG00000010011	LMNA	0,437414
ENSOCUG00000015796	ANXA4	0,436404
ENSOCUG00000005781	SUFU	0,435635
ENSOCUG00000001124	ZC2HC1A	0,435113
ENSOCUG00000023629	TPCN1	0,434526
ENSOCUG00000000476	FGF2	0,431436
ENSOCUG00000026842	LPAR1	0,430614
ENSOCUG00000009340	ARAP3	0,430141
ENSOCUG00000009772	C8orf76	0,429534
ENSOCUG00000011389	ABCG2	0,429516
ENSOCUG00000006022	MINDY4	0,427524
ENSOCUG00000006957	IL1R1	0,427192
ENSOCUG00000001299	MICAL2	0,42603
ENSOCUG00000003993	RFTN2	0,424892
ENSOCUG00000011626	CAMK1D	0,424686
ENSOCUG00000000614	KLHL11	0,421282
ENSOCUG00000002259	MPZL1	0,417902
ENSOCUG00000005447	ST5	0,410446
ENSOCUG00000009891	SMARCAL1	0,409476
ENSOCUG00000020935	MTCH1	0,40827
ENSOCUG00000001429	MAOA	0,407627
ENSOCUG00000002613	VCAN	0,407298
ENSOCUG00000003379	NUP54	0,40598
ENSOCUG00000003187	REM1	0,403482
ENSOCUG00000009694	SRGAP1	0,402915
ENSOCUG00000008171	GATA2	0,400431
ENSOCUG00000017052	BCOR	0,400315
ENSOCUG00000002090	RASSF2	0,397296
ENSOCUG00000010140	LZTFL1	0,396536
ENSOCUG00000016184	ATL2	0,392829
ENSOCUG00000012809	SLC25A24	0,391749
ENSOCUG00000017763	TLR3	0,391284
ENSOCUG00000009284	KCTD6	0,391196
ENSOCUG00000014339	COMMD7	0,385752
ENSOCUG00000027924	SIRT2	0,385651
ENSOCUG00000009222	VIM	0,3853
ENSOCUG00000028186	IRF9	0,384645
ENSOCUG00000001220	TUBGCP2	0,384645
ENSOCUG00000021890	WBP1L	0,384428
ENSOCUG00000001616	PCSK5	0,384291

ENSOCUG00000010843	PRKCD	0,383542
ENSOCUG00000004686	TENM4	0,383225
ENSOCUG00000008872	AARS	0,382308
ENSOCUG00000009508	ADPRH	0,38199
ENSOCUG00000002180	DLG5	0,379795
ENSOCUG00000001062	C8orf37	0,378868
ENSOCUG00000011893	GNB5	0,378461
ENSOCUG00000001272	MFSD1	0,378024
ENSOCUG00000012337	COL21A1	0,377769
ENSOCUG00000012790	CAP1	0,377753
ENSOCUG00000005078	STAT5B	0,376905
ENSOCUG00000015400	PTPN14	0,376722
ENSOCUG00000010245	AC022826.2	0,375939
ENSOCUG00000005893	NFIA	0,37325
ENSOCUG00000004519	ERCC8	0,372528
ENSOCUG00000017326	TUBGCP4	0,370683
ENSOCUG00000008047	VWA3A	0,370127
ENSOCUG00000004169	ASAH1	0,369248
ENSOCUG00000013024	NAA16	0,366603
ENSOCUG00000029614	PPM1D	0,36522
ENSOCUG00000016320	ATP8B2	0,365074
ENSOCUG00000001386	CTTNBP2	0,364181
ENSOCUG00000017667	ATP1A2	0,36331
ENSOCUG00000029231	ERGIC1	0,362909
ENSOCUG00000006656	DAAM2	0,362701
ENSOCUG00000012639	LMO4	0,360298
ENSOCUG00000008555	PLEKHM1	0,358631
ENSOCUG00000021918	DGKA	0,358117
ENSOCUG00000006096	ANKRD50	0,357316
ENSOCUG00000016720	SZT2	0,357014
ENSOCUG00000017791	SGPL1	0,354548
ENSOCUG00000013175	FBLN5	0,354314
ENSOCUG00000008574	HDAC5	0,347864
ENSOCUG00000007483	TBC1D12	0,347806
ENSOCUG00000006927	ARHGAP42	0,347211
ENSOCUG00000016386	MBTPS2	0,346884
ENSOCUG00000004878	ORC3	0,346357
ENSOCUG00000012643	OSMR	0,344381
ENSOCUG00000012677	ENTPD1	0,340657
ENSOCUG00000005905	STXBP5	0,339064
ENSOCUG00000002396	PKD2	0,337711
ENSOCUG00000005170	BRWD3	0,337622

ENSOCUG00000017929	Polr2a	0,337177
ENSOCUG00000010430	KIAA0907	0,33665
ENSOCUG00000015035	HECTD2	0,336199
ENSOCUG00000009330	FMO5	0,334799
ENSOCUG00000004135	GPATCH2L	0,334648
ENSOCUG00000026715	ERLIN1	0,334631
ENSOCUG00000012156	ATF2	0,328285
ENSOCUG00000002593	PI4KB	0,328002
ENSOCUG00000000810	PRICKLE2	0,327973
ENSOCUG00000005808	PDLIM3	0,325466
ENSOCUG00000016849	GABPB1	0,325285
ENSOCUG00000011193	SYTL4	0,324195
ENSOCUG00000003636	TGM2	0,321104
ENSOCUG00000011064	CLSTN1	0,320646
ENSOCUG00000007201	RNF138	0,317277
ENSOCUG00000007589	TRAFD1	0,316336
ENSOCUG00000011398	PPP3CA	0,313303
ENSOCUG00000005197	ABR	0,309311
ENSOCUG00000015226	RETREG3	0,307644
ENSOCUG00000010217	PSMC2	0,307325
ENSOCUG00000011947	YEATS2	0,306676
ENSOCUG00000002394	WASF2	0,306108
ENSOCUG00000008661	ZNF706	0,305953
ENSOCUG00000003479	CD34	0,304555
ENSOCUG00000010961	RPL15	0,304399
ENSOCUG00000029165	DIS3L2	0,30365
ENSOCUG00000006539	ATP2B4	0,301742
ENSOCUG00000022666	AQP1	0,301256
ENSOCUG00000004146	ANXA2	0,300142
ENSOCUG00000021712	NFKB1	0,299349
ENSOCUG00000015431	DOCK10	0,298705
ENSOCUG00000027213	RHOC	0,298022
ENSOCUG00000027712	RPL3	0,296205
ENSOCUG00000014568	MTMR2	0,291549
ENSOCUG00000004973	DIAPH1	0,290722
ENSOCUG00000007839	IL6ST	0,286543
ENSOCUG00000006695	RBM4B	0,285995
ENSOCUG00000017847	TMTC2	0,285278
ENSOCUG00000005101	LAMB2	0,285132
ENSOCUG00000017383	MIGA1	0,284712
ENSOCUG00000012354	SNX25	0,282563
ENSOCUG00000004012	FBXO28	0,280275

ENSOCUG00000005010	ACSL4	0,279031
ENSOCUG00000026099	PMP22	0,278521
ENSOCUG00000014503	NCSTN	0,277348
ENSOCUG00000012946	KDM5A	0,275678
ENSOCUG00000001711	TRO	0,273894
ENSOCUG00000006327	EDC4	0,273794
ENSOCUG00000011535	GOLPH3	0,271188
ENSOCUG00000005108	UBTD2	0,270193
ENSOCUG00000002897	CUL9	0,269817
ENSOCUG00000009155	MAP3K1	0,269708
ENSOCUG00000003285	ELMO1	0,269407
ENSOCUG00000002562	PODN	0,269186
ENSOCUG00000002495	PIK3R4	0,268365
ENSOCUG00000001620	SND1	0,267043
ENSOCUG00000004429	TRIM38	0,265519
ENSOCUG00000015698	RARS	0,26246
ENSOCUG00000004784	MCM3AP	0,262193
ENSOCUG00000001834	ODF2L	0,261992
ENSOCUG00000024739	ZBED6	0,260548
ENSOCUG00000007336	TMX4	0,25641
ENSOCUG00000008376	UBA1	0,256034
ENSOCUG00000006032	KANSL3	0,255094
ENSOCUG00000012396	ZMYM3	0,252852
ENSOCUG00000000919	ADGRA3	0,251026
ENSOCUG00000006967	PPP1R21	0,249504
ENSOCUG00000015963	TGFBR2	0,249128
ENSOCUG00000013582	ARNT	0,24881
ENSOCUG00000002809	PTPRG	0,24863
ENSOCUG00000013364	COPZ1	0,248304
ENSOCUG00000010472	SLC30A5	0,246163
ENSOCUG00000017525	MED13L	0,24548
ENSOCUG00000011357	ITGA1	0,243546
ENSOCUG00000012477	DNPEP	0,240855
ENSOCUG00000008062	ABI2	0,24013
ENSOCUG00000006510	C9orf72	0,239951
ENSOCUG00000009879	STAG2	0,238021
ENSOCUG00000008787	RECQL	0,237881
ENSOCUG00000012996	MAP4K5	0,232453
ENSOCUG00000006797	MED12	0,229833
ENSOCUG00000003781	MAPRE2	0,22944
ENSOCUG00000011300	CYP4V2	0,229326
ENSOCUG00000023881	SEC24C	0,227569

ENSOCUG00000013162	MARC2	0,225246
ENSOCUG00000001741	FTO	0,225068
ENSOCUG00000011678	EXOC4	0,215885
ENSOCUG00000017704	TBC1D23	0,215763
ENSOCUG00000004646	RASA1	0,215403
ENSOCUG00000004087	SRSF7	0,214817
ENSOCUG00000005516	ARPC2	0,210868
ENSOCUG00000000005	MDM4	0,210408
ENSOCUG00000000828	FLNB	0,209278
ENSOCUG00000017148	RYK	0,204441
ENSOCUG00000015460	NBAS	0,201272
ENSOCUG00000012765	CEP192	0,19068
ENSOCUG00000001943	KCTD3	0,189263
ENSOCUG00000012013	DIP2B	0,188916
ENSOCUG00000008757	MYOF	0,183899
ENSOCUG00000008940	TRA2B	0,179067
ENSOCUG00000012121	U2SURP	0,174109
ENSOCUG00000003815	ZFX	0,16089
ENSOCUG00000001155	PDIA3	0,157678
ENSOCUG00000008856	HELZ	0,157542
ENSOCUG00000008725	VPS13B	0,138422
ENSOCUG00000017819	USP34	0,113389
ENSOCUG00000003816	HECTD1	-0,11883
ENSOCUG00000015231	DCTN1	-0,15232
ENSOCUG00000013468	XRN2	-0,15689
ENSOCUG00000015531	CEP350	-0,16047
ENSOCUG00000002022	RAB3GAP1	-0,17314
ENSOCUG00000008957	NFX1	-0,18342
ENSOCUG00000007844	SOS1	-0,19111
ENSOCUG00000008521	YWHAG	-0,19619
ENSOCUG00000017692	AL139011.2	-0,19853
ENSOCUG00000017619	DNAJC3	-0,19887
ENSOCUG00000008495	PSMD11	-0,19927
ENSOCUG00000003925	KRR1	-0,20187
ENSOCUG00000008096	RPRD1A	-0,20255
ENSOCUG00000004304	DDX42	-0,20339
ENSOCUG00000025121	ALDH6A1	-0,2044
ENSOCUG00000009935	NBN	-0,20531
ENSOCUG00000014722	CSNK2A1	-0,20872
ENSOCUG00000008145	SMNDC1	-0,20953
ENSOCUG00000004346	RBBP6	-0,21658
ENSOCUG00000004401	MPDZ	-0,21773

ENSOCUG00000007258	STRN	-0,2231
ENSOCUG00000006823	APPL1	-0,22487
ENSOCUG00000006858	SEH1L	-0,22592
ENSOCUG00000010995	SNAP23	-0,22642
ENSOCUG00000007312	CCDC82	-0,22643
ENSOCUG00000009297	RAB6C	-0,2285
ENSOCUG00000001821	ABCE1	-0,22943
ENSOCUG00000013847	TCP1	-0,23065
ENSOCUG00000016756	NEK1	-0,23183
ENSOCUG00000009541	NFU1	-0,23252
ENSOCUG00000011751	DPP8	-0,23518
ENSOCUG00000001753	ZFYVE16	-0,23692
ENSOCUG00000002372	PGRMC2	-0,24056
ENSOCUG00000015413	PDCD10	-0,24383
ENSOCUG00000017240	CDC5L	-0,24481
ENSOCUG00000008135	GATAD2B	-0,24736
ENSOCUG00000006143	DYNC1LI2	-0,24911
ENSOCUG00000016531	AL512506.3	-0,25
ENSOCUG00000010945	ISCA1	-0,25251
ENSOCUG00000001127	RHOQ	-0,25303
ENSOCUG00000027628	ARL14EP	-0,25654
ENSOCUG00000012457	GOLGB1	-0,25718
ENSOCUG00000023995	CUEDC2	-0,2575
ENSOCUG00000008134	CHN1	-0,25848
ENSOCUG00000008565	PSMD1	-0,259
ENSOCUG00000004330	NRDC	-0,25962
ENSOCUG00000001047	SMARCA1	-0,26024
ENSOCUG00000013302	CTR9	-0,26092
ENSOCUG00000013470	NDFIP2	-0,2618
ENSOCUG00000011619	HBS1L	-0,26204
ENSOCUG00000001818	KPNA4	-0,26276
ENSOCUG00000008615	VAPA	-0,26607
ENSOCUG00000014783	NSF	-0,26667
ENSOCUG00000014347	AC090527.2	-0,26848
ENSOCUG00000029502	MICU2	-0,27086
ENSOCUG00000000925	GGNBP2	-0,27213
ENSOCUG00000029016	IPO8	-0,27254
ENSOCUG00000003961	TXNRD1	-0,27266
ENSOCUG00000006516	ZEB1	-0,27267
ENSOCUG00000006735	NMT1	-0,273
ENSOCUG00000002429	CCT7	-0,27542
ENSOCUG00000017091	MBIP	-0,27564

ENSOCUG00000015766	PATJ	-0,2763
ENSOCUG00000017004	TMEM182	-0,28026
ENSOCUG00000009768	PHF20L1	-0,28131
ENSOCUG00000012875	HSPA14	-0,28417
ENSOCUG00000017724	IMPDH2	-0,28764
ENSOCUG00000014056	DOCK5	-0,28768
ENSOCUG00000010755	PLCB4	-0,28825
ENSOCUG00000004358	CACYBP	-0,29153
ENSOCUG00000004392	FOXN3	-0,29467
ENSOCUG00000008341	ADHFE1	-0,29499
ENSOCUG00000003199	LMAN1	-0,29933
ENSOCUG00000004533	PTPN11	-0,29942
ENSOCUG00000000778	DENND5B	-0,3072
ENSOCUG00000022470	DRG1	-0,30914
ENSOCUG00000001696	DNAJC15	-0,30966
ENSOCUG00000003198	MIPOL1	-0,31105
ENSOCUG00000008440	FBXW7	-0,31169
ENSOCUG00000002637	FAM122A	-0,31172
ENSOCUG00000004815	BAG2	-0,31998
ENSOCUG00000008020	EYA3	-0,32118
ENSOCUG00000003055	RNF115	-0,32124
ENSOCUG00000009762	MTR	-0,32507
ENSOCUG00000000792	EPS8	-0,32605
ENSOCUG00000024670	ZNF770	-0,33035
ENSOCUG00000022143	G3BP1	-0,33131
ENSOCUG00000015654	TRAK1	-0,33232
ENSOCUG00000007950	CPEB2	-0,33259
ENSOCUG00000016946	KRCC1	-0,33283
ENSOCUG00000009575	FAM96A	-0,33658
ENSOCUG00000001565	MEA1	-0,33874
ENSOCUG00000004742	USP25	-0,34072
ENSOCUG00000017915	GUF1	-0,34321
ENSOCUG00000005077	TOPORS	-0,34383
ENSOCUG00000012201	PPAT	-0,34924
ENSOCUG00000017367	KLHDC1	-0,35246
ENSOCUG00000017234	GSR	-0,35296
ENSOCUG00000004281	PTDSS1	-0,35326
ENSOCUG00000002748	S100A13	-0,3547
ENSOCUG00000012582	RAB21	-0,35489
ENSOCUG00000015750	UBA5	-0,35688
ENSOCUG00000005872	UBE2K	-0,35851
ENSOCUG00000007930	ATF6	-0,36081

ENSOCUG00000002334	SKA2	-0,36098
ENSOCUG00000015261	SEPHS1	-0,36186
ENSOCUG00000006467	NAA50	-0,36302
ENSOCUG00000000884	POLR3B	-0,36424
ENSOCUG00000004757	ZNF35	-0,36437
ENSOCUG00000016581	RALA	-0,36447
ENSOCUG00000005288	UBE2J1	-0,36624
ENSOCUG00000011635	ACAD9	-0,36724
ENSOCUG00000026538	PHB2	-0,37072
ENSOCUG00000012736	HSPD1	-0,37453
ENSOCUG00000021196	EPB41L3	-0,37566
ENSOCUG00000000004	FGD1	-0,37703
ENSOCUG00000011189	MRPL22	-0,37785
ENSOCUG00000013919	FGD4	-0,37911
ENSOCUG00000008203	NOP2	-0,38257
ENSOCUG00000011809	RBMX2	-0,38485
ENSOCUG00000008393	OSBPL1A	-0,38664
ENSOCUG00000004942	PRKAA2	-0,38687
ENSOCUG00000000626	AS3MT	-0,38799
ENSOCUG00000003639	CREB5	-0,3882
ENSOCUG00000012693	LDB3	-0,38996
ENSOCUG00000004551	BEND7	-0,39035
ENSOCUG00000013637	GPN1	-0,39066
ENSOCUG00000009953	SLC25A13	-0,39163
ENSOCUG00000010928	MRPL42	-0,39227
ENSOCUG00000006548	ECHDC1	-0,39459
ENSOCUG00000011720	MTHFD1	-0,39747
ENSOCUG00000012980	AC093899.2	-0,40232
ENSOCUG00000014849	CACNB2	-0,40591
ENSOCUG00000002694	KPNA1	-0,40694
ENSOCUG00000004785	DAP3	-0,41033
ENSOCUG00000016776	SLC16A1	-0,41104
ENSOCUG00000006086	LCLAT1	-0,41159
ENSOCUG00000012433	ME2	-0,41183
ENSOCUG00000015156	TWISTNB	-0,41209
ENSOCUG00000001598	PAM	-0,41276
ENSOCUG00000010185	ALDH7A1	-0,41335
ENSOCUG00000000968	DES	-0,41374
ENSOCUG00000000285	LBR	-0,41628
ENSOCUG00000006359	NDUFS3	-0,4163
ENSOCUG00000015685	SSX2IP	-0,41763
ENSOCUG00000013139	BCKDHB	-0,42375

ENSOCUG00000011265	ZSCAN29	-0,42501
ENSOCUG00000000482	NUDT6	-0,42735
ENSOCUG00000012393	MCCC1	-0,4274
ENSOCUG00000001250	EDNRA	-0,42821
ENSOCUG00000013943	CISD2	-0,42852
ENSOCUG00000001981	CNOT9	-0,42861
ENSOCUG00000006463	PRKAG2	-0,42954
ENSOCUG00000016540	ACAA2	-0,42984
ENSOCUG00000015820	MXD1	-0,43136
ENSOCUG00000013720	CLYBL	-0,43138
ENSOCUG00000007355	DSG2	-0,43163
ENSOCUG00000008429	CTNNA3	-0,43183
ENSOCUG00000022291	GYPC	-0,43258
ENSOCUG00000015583	RNF6	-0,4347
ENSOCUG00000017887	NDUFB5	-0,43534
ENSOCUG00000025068	KIF1C	-0,43667
ENSOCUG00000008250	SAR1B	-0,43685
ENSOCUG00000025332	RDH14	-0,43687
ENSOCUG00000004685	ENY2	-0,44053
ENSOCUG00000009680	HOMER1	-0,4449
ENSOCUG00000010554	WDR46	-0,44542
ENSOCUG00000022791	ART1	-0,44559
ENSOCUG00000012328	RNF128	-0,44846
ENSOCUG00000012792	THNSL1	-0,45022
ENSOCUG00000003038	CRYZ	-0,45132
ENSOCUG00000001101	SLC25A11	-0,45157
ENSOCUG00000004573	TGIF2-C20orf24	-0,45266
ENSOCUG00000007239	NDUFAF7	-0,45298
ENSOCUG00000016191	PPFIBP1	-0,45308
ENSOCUG00000026462	LUZP2	-0,45419
ENSOCUG00000014274	MNAT1	-0,45484
ENSOCUG00000014535	IMPA1	-0,45797
ENSOCUG00000007277	GPALPP1	-0,46096
ENSOCUG00000003071	LPIN1	-0,46264
ENSOCUG00000009899	NDUFV2	-0,46278
ENSOCUG00000010438	KLHL5	-0,46282
ENSOCUG00000006751	LYPLA1	-0,46327
ENSOCUG00000010138	POLR1A	-0,46423
ENSOCUG00000000790	NDUFS4	-0,46933
ENSOCUG00000029140	NDUFB4	-0,47068
ENSOCUG00000009450	ARHGAP18	-0,4768
ENSOCUG00000000984	ESRRG	-0,47732

ENSOCUG00000016980	UTP6	-0,47858
ENSOCUG00000029590	GSTM4	-0,4799
ENSOCUG00000026497	MRPS14	-0,48015
ENSOCUG00000012376	SUCLG2	-0,48079
ENSOCUG00000002231	VLDLR	-0,48296
ENSOCUG00000006485	MRPL39	-0,48365
ENSOCUG00000016362	ADAMTS1	-0,48636
ENSOCUG00000003153	TRIB2	-0,48691
ENSOCUG00000000827	SLMAP	-0,48759
ENSOCUG00000009623	AL049844.3	-0,48777
ENSOCUG00000010654	ARHGAP24	-0,48886
ENSOCUG00000002249	DHRS7	-0,49006
ENSOCUG00000006950	CECR2	-0,49185
ENSOCUG00000014070	ZNF697	-0,49317
ENSOCUG00000024341	Mphosph6	-0,49532
ENSOCUG00000025658	AAMDC	-0,49548
ENSOCUG00000012467	NANS	-0,49567
ENSOCUG00000002882	ACAT1	-0,50057
ENSOCUG00000006217	CLEC16A	-0,50135
ENSOCUG00000015648	PPP1R11	-0,50452
ENSOCUG00000014062	TIMM21	-0,50774
ENSOCUG00000004067	CNST	-0,51094
ENSOCUG00000003372	ART3	-0,51187
ENSOCUG00000005016	NT5C3A	-0,51424
ENSOCUG00000003001	TNNI3K	-0,51519
ENSOCUG00000002780	PANK1	-0,51731
ENSOCUG00000024043	ATP5I	-0,51733
ENSOCUG00000006990	PHF23	-0,51805
ENSOCUG00000003267	ENSA	-0,51896
ENSOCUG00000009270	SYNPO	-0,52047
ENSOCUG00000023095	TRMT13	-0,52075
ENSOCUG00000024417	NDUFS5	-0,5228
ENSOCUG00000021424	VPS72	-0,52382
ENSOCUG00000025314	GABRA4	-0,52582
ENSOCUG00000000989	AL133352.1	-0,52728
ENSOCUG00000004834	F3	-0,52903
ENSOCUG00000022295	COX17	-0,5296
ENSOCUG00000015894	PYURF	-0,53378
ENSOCUG00000007216	CS	-0,53556
ENSOCUG00000009191	SLC25A32	-0,53999
ENSOCUG00000008225	LACTB2	-0,54015
ENSOCUG00000026215	TMEM208	-0,54767

ENSOCUG00000023516	KNOP1	-0,54792
ENSOCUG00000006998	C11orf74	-0,55288
ENSOCUG00000009204	SEC11C	-0,55391
ENSOCUG00000009581	TACR3	-0,55429
ENSOCUG00000021735	ITGA7	-0,56014
ENSOCUG00000000516	NOLC1	-0,56016
ENSOCUG00000000141	ACTR6	-0,56086
ENSOCUG00000010594	CASQ2	-0,56355
ENSOCUG00000000164	LAMTOR1	-0,56392
ENSOCUG00000016823	COBL	-0,5688
ENSOCUG00000021716	DHRS4L2	-0,56942
ENSOCUG00000029354	MEF2C	-0,57622
ENSOCUG00000001030	GEM	-0,5799
ENSOCUG00000014444	HDAC9	-0,57998
ENSOCUG00000021458	FAHD2A	-0,58018
ENSOCUG00000007642	DEPTOR	-0,58089
ENSOCUG00000004163	SLC25A33	-0,58228
ENSOCUG00000017713	NUBPL	-0,58664
ENSOCUG00000015408	ACYP2	-0,59139
ENSOCUG00000016954	CCDC141	-0,59415
ENSOCUG00000014322	MDH2	-0,59596
ENSOCUG00000002683	TUFM	-0,59638
ENSOCUG00000026731	HELLS	-0,60278
ENSOCUG00000004936	NAV3	-0,6037
ENSOCUG00000006903	SETD6	-0,60442
ENSOCUG00000001053	THG1L	-0,60756
ENSOCUG00000009532	AFF3	-0,60922
ENSOCUG00000002449	LAMTOR5	-0,61127
ENSOCUG00000013506	TCAIM	-0,61585
ENSOCUG00000013296	GADD45G	-0,61717
ENSOCUG00000001383	MRPL9	-0,61793
ENSOCUG00000013881	XPA	-0,61899
ENSOCUG00000008900	UBE2D4	-0,61938
ENSOCUG00000014894	AC005833.3	-0,62087
ENSOCUG00000001952	TIGAR	-0,62149
ENSOCUG00000007562	BOLA2	-0,62318
ENSOCUG00000009230	ATPAF1	-0,62873
ENSOCUG00000029598	PINX1	-0,6303
ENSOCUG00000005617	ACSL1	-0,63162
ENSOCUG00000026348	KCNIP2	-0,63202
ENSOCUG00000012368	NDUFS1	-0,63871
ENSOCUG00000027206	HINT2	-0,64056

ENSOCUG00000006597	CLGN	-0,64638
ENSOCUG00000001498	CIAO1	-0,6536
ENSOCUG00000011557	AC092718.8	-0,66011
ENSOCUG00000025335	SYNPO2L	-0,66059
ENSOCUG00000021141	APIP	-0,66095
ENSOCUG00000027853	PGAM2	-0,66125
ENSOCUG00000017334	PRDX3	-0,66382
ENSOCUG00000027846	DNAJB5	-0,67399
ENSOCUG00000016473	C2orf69	-0,67511
ENSOCUG00000005816	SORBS2	-0,67796
ENSOCUG00000014369	GCNT2	-0,67995
ENSOCUG00000013141	BLOC1S2	-0,68511
ENSOCUG00000014618	RGS10	-0,68597
ENSOCUG00000011552	YEATS4	-0,69153
ENSOCUG00000017243	FAR2	-0,69365
ENSOCUG00000002803	C1orf52	-0,69429
ENSOCUG00000007427	FAT3	-0,6994
ENSOCUG00000006748	HEXIM1	-0,70373
ENSOCUG00000001676	EZH2	-0,71118
ENSOCUG00000002682	CCDC58	-0,71881
ENSOCUG00000017601	CDH19	-0,73237
ENSOCUG00000002826	CADPS	-0,73596
ENSOCUG00000017721	NDUFAF3	-0,73669
ENSOCUG00000006901	ANKRD1	-0,73822
ENSOCUG00000004074	ACTC1	-0,7387
ENSOCUG00000001970	AKAP3	-0,75616
ENSOCUG00000022444	DECR1	-0,75673
ENSOCUG00000014414	MYOT	-0,76132
ENSOCUG00000016658	BOLA3	-0,76238
ENSOCUG00000003806	KY	-0,76427
ENSOCUG00000013763	DLGAP1	-0,76512
ENSOCUG00000007600	UQCRH	-0,76742
ENSOCUG00000008892	RAPSN	-0,77091
ENSOCUG00000017085	COX5B	-0,77169
ENSOCUG00000002909	GRB14	-0,77188
ENSOCUG00000001880	MAGOH	-0,77301
ENSOCUG00000017178	C8A	-0,7756
ENSOCUG00000004621	SYT17	-0,7776
ENSOCUG00000025973	Popdc3	-0,77826
ENSOCUG00000029345	HMGN5	-0,77976
ENSOCUG00000009113	PDE1C	-0,80678
ENSOCUG00000014063	TCEA3	-0,81321

ENSOCUG00000012207	NEBL	-0,81872
ENSOCUG00000012422	COX10	-0,8221
ENSOCUG00000013727	TFB2M	-0,82262
ENSOCUG00000017002	MDGA2	-0,83825
ENSOCUG00000001082	CLIP4	-0,84663
ENSOCUG00000005261	MRPS36	-0,85959
ENSOCUG00000013627	UQCRQ	-0,87711
ENSOCUG00000013113	GDPD1	-0,88447
ENSOCUG00000013155	HHATL	-0,88616
ENSOCUG00000005512	RXRG	-0,88642
ENSOCUG00000006322	NUTF2	-0,90422
ENSOCUG00000009324	GJA1	-0,91815
ENSOCUG00000020895	VGLL3	-0,92629
ENSOCUG00000022531	CLIC5	-0,93527
ENSOCUG00000008858	CORIN	-0,95966
ENSOCUG00000013780	METTL18	-0,97617
ENSOCUG00000023345	PTRH2	-0,97666
ENSOCUG00000015216	LRRC3B	-0,97739
ENSOCUG00000022262	ST8SIA5	-0,98827
ENSOCUG00000006264	NMNAT3	-1,00201
ENSOCUG00000025456	MAP7	-1,00258
ENSOCUG00000016138	GRPEL2	-1,00778
ENSOCUG00000006103	CENPQ	-1,01045
ENSOCUG00000026304	C2orf40	-1,027
ENSOCUG00000011459	RYR3	-1,03775
ENSOCUG00000019739	SNORD87	-1,05895
ENSOCUG00000024444	CRHR2	-1,06724
ENSOCUG00000006020	PHEX	-1,06894
ENSOCUG00000002174	TEX12	-1,07013
ENSOCUG00000014052	LSAMP	-1,0745
ENSOCUG00000025915	MRPL48	-1,08565
ENSOCUG00000002752	S100A1	-1,10533
ENSOCUG00000016858	NKAPL	-1,13185
ENSOCUG00000006775	ANO2	-1,13342
ENSOCUG00000017458	NEB	-1,16658
ENSOCUG00000016105	COLQ	-1,16697
ENSOCUG00000019290	mir-30a	-1,16709
ENSOCUG00000022110	TCEAL7	-1,168
ENSOCUG00000021581	CETN2	-1,17284
ENSOCUG00000011793	C1QTNF9	-1,17521
ENSOCUG00000021989	NDUFA3	-1,19557
ENSOCUG00000006498	DSG1	-1,21142

ENSOCUG00000023908	4930503B20Rik	-1,22686
ENSOCUG00000017236	LRRC2	-1,22743
ENSOCUG00000009032	CA14	-1,22881
ENSOCUG00000006752	MEST	-1,22923
ENSOCUG00000027251	C1orf54	-1,24745
ENSOCUG00000024952	SNORD71	-1,29528
ENSOCUG00000025586	MYL2	-1,32108
ENSOCUG00000018756	SNORA4	-1,33339
ENSOCUG00000009843	SPOCK1	-1,35872
ENSOCUG00000013450	MYH11	-1,36065
ENSOCUG00000016280	CXCL10	-1,40318
ENSOCUG00000027477	TIMM8A	-1,43643
ENSOCUG00000001788	RASGRP1	-1,44729
ENSOCUG00000008188	LMOD3	-1,53238
ENSOCUG00000019311	SNORD65	-1,55584
ENSOCUG00000007663	MRO	-1,55673
ENSOCUG00000001849	SMPX	-1,58397
ENSOCUG00000027484	PPP1R3E	-1,62163
ENSOCUG00000017551	XIRP2	-1,70959
ENSOCUG00000014210	HNRNPA1L2	-1,75053
ENSOCUG00000021483	SNORD121A	-1,79976
ENSOCUG00000008469	MOXD1	-1,81034
ENSOCUG00000008305	CELA1	-1,83856
ENSOCUG00000018248	snRNA	-1,87263
ENSOCUG00000000737	NR4A3	-1,88179
ENSOCUG00000023253	SNORD47	-2,14709
ENSOCUG00000017742	FHL5	-2,16602
ENSOCUG00000016623	FGFBP1	-2,18782
ENSOCUG00000024035	SNORD74	-2,28694
ENSOCUG00000019647	SNORA26	-2,33283
ENSOCUG00000022519	MXD3	-2,59024
ENSOCUG00000017645	GALNT13	-2,74425
ENSOCUG00000027750	RPL21	-3,78025
ENSOCUG00000024282	PLEKHB2	-4,41459

Table S2: Sequences of primers used for qPCR.

Gene	Forward (5′-3′)	Reverse (5′-3′)
SNORA63	CCTGCTCAAAGTAAGGTTGTTTC	AGGGATAGAGCAGACAGGTATAG
COL1A2	ATGGTGGCACCCAGTTTGAA	GTGCAGCCATCGACAAGAAC
MGP	GCGAACACCTTTATGTCGCC	ACCATGGCATAGCGTTCACA
RPL11	GTCTGAAGGTGCGGGAGTAT	TAGATGCCGATGCTTGGGTC
HAPLN1	CCCGTGAAGTTTGCACGAAGT	CCAGGAATCTTCCTCACTGGTC
MIR107	TTACAGTGTTGCCTTGTGGC	TAGCCCTGTAAATGCTGCT
